# Genome-wide adenine N6-methylation map reveals epigenomic regulation of lipid accumulation in *Nannochloropsis*

**DOI:** 10.1016/j.xplc.2023.100773

**Published:** 2023-11-24

**Authors:** Yanhai Gong, Qintao Wang, Li Wei, Wensi Liang, Lianhong Wang, Nana Lv, Xuefeng Du, Jiashun Zhang, Chen Shen, Yi Xin, Luyang Sun, Jian Xu

**Affiliations:** 1 Single-Cell Center, CAS Key Laboratory of Biofuels, Shandong Key Laboratory of Energy Genetics, Qingdao Institute of Bioenergy and Bioprocess Technology, Chinese Academy of Sciences, Qingdao, China; 2 Shandong Energy Institute, Qingdao, China; 3 Qingdao New Energy Shandong Laboratory, Qingdao, China; 4 University of Chinese Academy of Sciences, Beijing 100049, China; 5 Laboratory for Marine Biology and Biotechnology, Qingdao National Laboratory for Marine Science and Technology, Qingdao 266237, China

**Keywords:** adenine N6-methylation, industrial oleaginous microalgae, *Nannochloropsis oceanica*, transcriptional regulation, epigenomics

## Abstract

Epigenetic marks on histones and DNA, such as DNA methylation at N6-adenine (6mA), play crucial roles in gene expression and genome maintenance, but their deposition and function in microalgae remain largely uncharacterized. Here, we report a genome-wide 6mA map for the model industrial oleaginous microalga *Nannochloropsis oceanica* produced by single-molecule real-time sequencing. Found in 0.1% of adenines, 6mA sites are mostly enriched at the AGGYV motif, more abundant in transposons and 3′ untranslated regions, and associated with active transcription. Moreover, 6mA gradually increases in abundance along the direction of gene transcription and shows special positional enrichment near splicing donor and transcription termination sites. Highly expressed genes tend to show greater 6mA abundance in the gene body than do poorly expressed genes, indicating a positive interaction between 6mA and general transcription factors. Furthermore, knockout of the putative 6mA methylase NO08G00280 by genome editing leads to changes in methylation patterns that are correlated with changes in the expression of molybdenum cofactor, sulfate transporter, glycosyl transferase, and lipase genes that underlie reductions in biomass and oil productivity. By contrast, knockout of the candidate demethylase NO06G02500 results in increased 6mA levels and reduced growth. Unraveling the epigenomic players and their roles in biomass productivity and lipid metabolism lays a foundation for epigenetic engineering of industrial microalgae.

## Teaser

This study reports a genome-wide map of 6mA modifications for *Nannochloropsis oceanica,* revealing the structural and functional features of this epigenomic modification in an industrial oleaginous microalga. Gene knockout of a methylase and a demethylase revealed correlations between 6mA levels, gene expression, and production of oil and biomass. Unraveling the epigenomic players and their roles in biomass productivity and lipid metabolism lays a foundation for epigenetic engineering of industrial microalgae.

## Introduction

DNA N6-methyladenine (6mA), a non-canonical DNA modification present at low levels in eukaryotes, has emerged as an important epigenetic marker (Liang et al., 2018a). In prokaryotes, 6mA regulates DNA replication and repair, virulence, and gene expression (Wright et al., 1997; Julio et al., 2001; Kahng and Shapiro, 2001; Reisenauer and Shapiro, 2002). In eukaryotes, the genome-wide distribution and function of 6mA were largely unknown until recent reports on unicellular green algae (Fu et al., 2015), fungi (Mondo et al., 2017), animals (Greer et al., 2015; Zhang et al., 2015; Wu et al., 2016; Wang et al., 2017; Yao et al., 2017; Ma et al., 2018), and plants (Zhang et al., 2018; Zhe et al., 2018; Zhou et al., 2018). The functions of 6mA appear to be quite divergent among eukaryotes. For example, 6mA is enriched around transcription start sites (TSSs) and its association with active transcription is conserved among *Chlamydomonas*, early-diverging fungi, and *Arabidopsis* (Fu et al., 2015; Mondo et al., 2017; Zhe et al., 2018). However, in rice, N6-methylated As in gene bodies can activate gene transcription, but those in promoter regions are involved in gene silencing (Zhang et al., 2018; Zhou et al., 2018); in *Tetrahymena*, 6mA is associated with genes transcribed by RNA polymerase II but is not correlated with active transcription (Wang et al., 2017). Another key role of 6mA is activation or repression of transposable elements (TEs), e.g., in *Drosophila* (Zhang et al., 2015) and mouse (Wu et al., 2016; Yao et al., 2017). Notably, green algae (*Chlamydomonas*), ciliates (*Tetrahymena*), and early-diverging fungi, which contain the AMT1 clade of MT-A70 methyltransferases (Wang et al., 2019), are the only species that show symmetrical distribution of 6mA at ApT dinucleotides (Fu et al., 2015; Mondo et al., 2017), suggesting the functional divergence of 6mA in unicellular eukaryotes.

Microalgae are diverse, unicellular, photosynthetic organisms that are responsible for one-half of global photosynthetic activity and primary production (Singh and Saxena, 2015). 6mA and 5mC have been found in the green algae *Chlamydomonas reinhardtii* (Hattman et al., 1978) and *Volvox carteri* (Babinger et al., 2001), but a whole-genome 6mA map is available only for *C*. *reinhardtii*, in which 6mA marks active TSSs (Fu et al., 2015). It is not clear whether patterns of 6mA are conserved in microalgal genomes, how such patterns are linked to gene regulation, or to what degree organismal phenotypes are altered by 6mA. Efforts to tackle these questions have been hindered by the genomic coexistence of 5mC, whose TE-silencing activities in animals, plants, fungi, and algae (such as diatoms) can mask or obscure the roles of 6mA (Deniz et al., 2019).

*Nannochloropsis* spp. are industrial feedstocks and leading research models for microalgal oil production (Dong et al., 2013; Li et al., 2014; Xin et al., 2017, 2019). Intriguingly, their genomes lack 5mC owing to the absence of DNA (cytosine-5) methyltransferases (Fan et al., 2020), suggesting that they may serve as exceptional models for the study of 6mA function. Here, we used the industrial photosynthetic oleaginous microalga *N*. *oceanica* as a model and produced a genome-wide map that reveals the distribution of 6mA on each chromosome and suggests its mode of transcriptional regulation. Two key genes controlling 6mA epigenomic modifications were identified and their functions in biomass productivity and lipid metabolism were demonstrated through knockout experiments. These findings lay a foundation for epigenetic engineering of industrial microalgae.

## Results

### Creating a genome-wide 6mA map for *N. oceanica*

**Figure 1:**
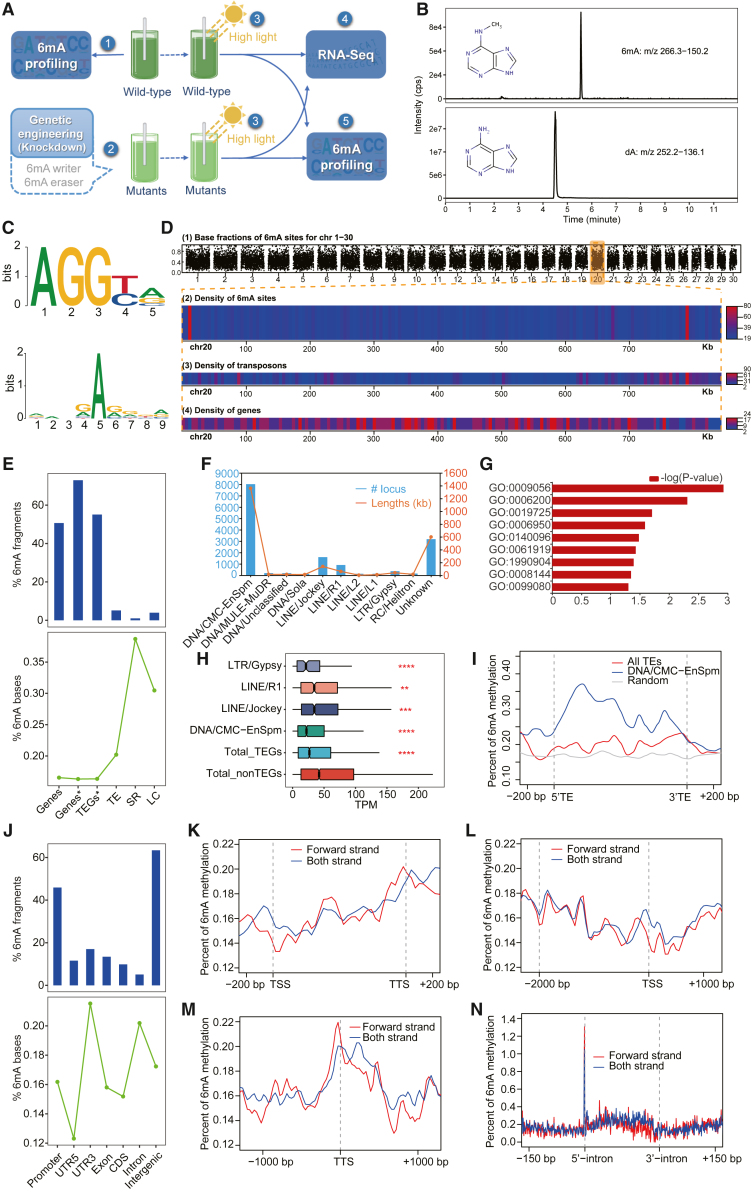
Distribution and global features of 6mA events in the *N*. *oceanica* genome **(A)** Experimental design of this study. **(B)** Mass spectra of 6mA and dA in isolated *N*. *oceanica* genomic DNA as detected by UPLC–MS/MS. **(C)** The motifs and sequence logo (bottom) that are enriched around 6mA sites (9mers). 6mA sites occur most frequently at GAGG motifs (top). **(D)** The frequencies of 6mA sites, TEs, and genes along the chromosomes suggest 6mA site preferences. Details for chromosome 20 are shown as an example. The heatmaps were drawn in 5-kb bins. **(E)** Proportions and base proportions of 6mA events for different genomic regions (strand specific). Genes, gene body; Genes^∗^, gene body and 2-kb promoter region; LC, low-complexity fragments; TEGs^∗^, Genes^∗^ that overlap with TEs. **(F)** Counts and lengths of different TE superfamilies in the *N*. *oceanica* genome. **(G)** Significant GO terms for CMC-EnSpm-associated genes (EnSpm-TEGs). **(H)** Association between TEGs and gene transcription implies that TEs such as DNA/CMC-EnSpm and LTR/Gypsy have a repressive effect. Asterisks indicate significance based on the Wilcoxon test (∗∗*p* < 0.01, ∗∗∗*p* < 0.001, ∗∗∗∗*p* < 0.0001). **(I)** 6mA occupancy around transposons. **(J–N)** Percentage of 6mA fragments (top) and percentage of 6mA bases (bottom) for various types of genomic regions (strand specific). Also shown are the 6mA occupancy around the gene body **(K)**, TSS **(L)**, TTS **(M)**, and introns **(N)**. For each gene, the gene body was consolidated into 1000 bp, and the intron was consolidated into 200 bp. The intron strand was defined as the strand of the same gene.

To examine the function of DNA methylation in *N*. *oceanica*, we employed a strategy that integrated genome-wide 6mA profiling and genetic perturbation of the 6mA machinery (Figure 1A). We started by improving the *N*. *oceanica* IMET1 genome assembly using ∼340× genome coverage of PacBio single-molecule real-time (SMRT) sequencing data and further scaffolding with additional Hi-C data (Gong et al., 2020) (Supplemental methods). This produced a high-quality reference genome with chromosome-scale assembly and superior base completeness (IMET1v2; details in the Supplemental results, Supplemental Table 1–4, and Supplemental Figure 1).

We next produced a genome-wide, single nucleotide resolution map of 6mA in *N*. *oceanica* by kinetic analysis of the SMRT data. Approximately 0.1% of the adenines were N6-methylated (6mA/A), a lower percentage than that reported for *C*. *reinhardtii* (Fu et al., 2015). Liquid chromatography tandem mass spectrometry (LC–MS/MS) is another approach for detecting and quantifying DNA methylation (Liang et al., 2018b). To further confirm the presence and level of 6mA in the *N*. *oceanica* genome, we detected 6mA and dA by an LC–MS/MS assay and quantified them using standard curves derived from 6mA and dA reference compounds. Clear peaks for 6mA and dA were observed in the corresponding mass spectra (Figure 1B), confirming the presence of 6mA in the *N*. *oceanica* genome. 6mA nucleotides accounted for 0.4% of all As (Figure 1B), again supporting the low abundance of 6mA in *N*. *oceanica*.

Motif mining of the SMRT data revealed preferences in 6mA positioning: (*i*) there were conserved guanine (G) bases next to 6mA sites (as shown in the sequence logo in Figure 1C); and (*ii*) AGGYV was the most abundant 6mA-associated motif in *N*. *oceanica* (Figure 1C and Supplemental Figure 2), similar to those of *C*. *elegans* (AGAA and GAGG) (Greer et al., 2015), *A*. *thaliana* (ANYGA, GAGG, and ACCT) (Zhe et al., 2018), and rice (AG and GAGG) (Zhou et al., 2018) but very distinct from that of *C*. *reinhardtii* (AT) (Fu et al., 2015). Thus, the base context of 6mA in *N*. *oceanica* appears more similar to that of higher plants than to that of green algae.

The map also reveals that (Figure 1D): (*i*) 6mA is widely distributed across the nuclear genome (Supplemental Figure 1), with the fraction of methylation typically ranging from 10% to 80% for each individual adenine; and (*ii*) the density of 6mA sites fluctuates across chromosomes. In the 5-kb resolution map, no strong correlation was observed between the density of 6mA sites and that of genes or transposons. However, in the single-base resolution map, we discovered regions with higher 6mA levels. Approximately 78% of the intervals between adjacent 6mA sites were <2 kbp (Supplemental Figure 3), and 2253 of the 6mA sites (9.2% of all 6mA sites) formed 126 densely methylated adenine clusters (“6mA hotspots”). Approximately 15% of these 6mA hotspots (19 of 126) were associated with TEs (vs. 9.8% on average; *p* < 0.05; permutation test), suggesting that 6mA hotspots are positively correlated with TEs. Thus, it appears that 6mA hotspots are selectively distributed in the *N*. *oceanica* genome and may have specific functions.

### Positional preferences of 6mA events in the *N. oceanica* genome

To further characterize 6mA site preferences, we examined various genomic features in a strand-specific (Figure 1E) and non-strand-specific manner (Supplemental Figure 4). In terms of the number of methylated regions (Figure 1E, top), ∼51% of protein-coding genes and 55% of transposable-element-associated genes (TEGs) were marked by at least one 6mA event, whereas repetitive elements, including TEs, simple repeats (SRs), and low-complexity sequences (LCs), were less prone to 6mA modification (Z-test, *p* < 0.001; e.g., only 5% of TEs were marked by 6mA). The density of 6mA-methylated bases (6mA/A) was much higher for repetitive elements (TEs, SRs, and LCs) than for protein-coding genes and TEGs (>0.20% vs. ∼0.16%) (Figure 1E, bottom).

TEs, which were widely distributed in the *N*. *oceanica* genome, were associated with 4361 genes (i.e., TEGs; 42.2%), and DNA/CMC-EnSpm (the CMC-EnSpm superfamily of DNA transposons) (Wei et al., 2016) accounted for ∼60% of the TEs (Figure 1F). Alignment of the TE and TEG distribution patterns revealed that TEs were inserted into both intragenic and intergenic positions of TEGs. There were 2420 TEs in coding sequences (CDSs) and 2553 TEs that overlapped with both introns and CDSs (Supplemental Figure 5); the latter may contribute to the functional evolution of TEGs. TEGs that were associated with DNA/CMC-EnSpm (EnSpm-TEGs) were enriched in Gene Ontology (GO) terms like catabolic process (GO:0009056), cellular homeostasis (GO:0019725), response to stress (GO:0006950), and others (Figure 1G). TEGs containing TEs from different superfamilies differed in transcription level (e.g., Wei et al., 2016), and the lowest transcription was observed for those containing DNA/CMC-EnSpm and LTR/Gypsy TEs (Wilcoxon test, *p* < 0.001) (Figure 1H). Three hundred and ninety-nine EnSpm-TEGs (13.0%) were differentially expressed under nitrate limitation (178 up- and 155 downregulated), a significantly higher percentage than the overall ratio (11.5%, 583 up- and 607 downregulated; binomial test, *p* < 0.01) (Supplemental Figure 5). Likewise, 352 EnSpm-TEGs (13.8%) were differentially expressed under CO_2_ limitation (297 up- and 55 downregulated), also a significantly higher percentage than the overall ratio (11.4%; 825 up- and 349 downregulated; binomial test, *p* < 0.001) (Supplemental Figure 5). 6mA-methylated bases (6mA/A) were enriched in TEs, especially DNA/CMC-EnSpm, compared with randomly selected genomic regions (similar to *Drosophila*; Zhang et al., 2015) (Figure 1I), suggesting that 6mA may contribute to the inhibition of TEG expression.

Genes consist of multiple regions, including promoters, introns, untranslated regions (UTRs), and exons. Comparison of the ratios of methylated regions (with at least one 6mA event) revealed higher 6mA occurrence in the promoter and intergenic regions than in exons or introns (Figure 1J, top). Based on the density of 6mA (defined as 6mA/A), 3′ UTR regions were much more methylated than promoters, introns, and exons (Figure 1J, bottom) (Z-test, *p* < 0.001), implying a potential role for 6mA in termination of transcription. Notably, 6mA density was similar in exons and introns (with introns being slightly higher) (Figure 1J), in contrast to *A*. *thaliana* and rice, in which most 6mA sites are found in exons (Supplemental Figure 6). The positional distribution of 6mA along the gene structure revealed that: (*i*) 6mA density increased gradually along the gene body (Figure 1K); (*ii*) the region around the TSS had the lowest 6mA density (Figure 1L); (*iii*) a prominent peak of 6mA density was found around the transcription termination site (TTS) but not the TSS (Figure 1M); and (*iv*) in introns, at least six times more 6mA modifications were located right before the splicing donor site (1–2 bp upstream) (Figure 1N), as also observed for *A*. *thaliana* and rice but not for *C*. *reinhardtii* (Supplemental Figure 6). However, unlike in *A*. *thaliana* and rice, the density of 6mA in *N. oceanica* was higher along introns than in the surrounding exon area (Supplemental Figure 6). This evidence, especially the marked abundance of 6mA around the TTS and 5′ intron, suggests roles for 6mA in transcription termination and co-transcriptional splicing.

The 6mA pattern of the *N*. *oceanica* genome is thus characterized by (*i*) no enrichment of 6mA in GATC motifs, unlike that of *C*. *reinhardtii* (Fu et al., 2015) and fungi (Mondo et al., 2017), and (*ii*) no enrichment of 6mA around the TSS or within exons, unlike that of rice (Zhou et al., 2018) and *A*. *thaliana* (Zhe et al., 2018). Regulation of gene transcription by 6mA in *N*. *oceanica* is likely distinct from that in these model organisms.

### Functional preference of 6mA events in the *N. oceanica* genome

To probe the functional consequences of 6mA modification, we correlated the presence and density of 6mA with those of various genome-encoded functional elements in *N*. *oceanica*. In non-coding genes, seven 6mA sites were found in four 28S rRNA genes and two 18S rRNA genes, suggesting a role for 6mA in RNA polymerase I–mediated gene transcription. However, no 6mA sites were found in genomic sequences of tRNAs and 5S rRNA, implying that 6mA is not involved in RNA polymerase III–mediated gene transcription. These observations appear to differ from those in other organisms such as *A. thaliana*, *C. elegans*, *Drosophila melanogaster*, and *Homo sapiens* (Li et al., 2020).

**Figure 2:**
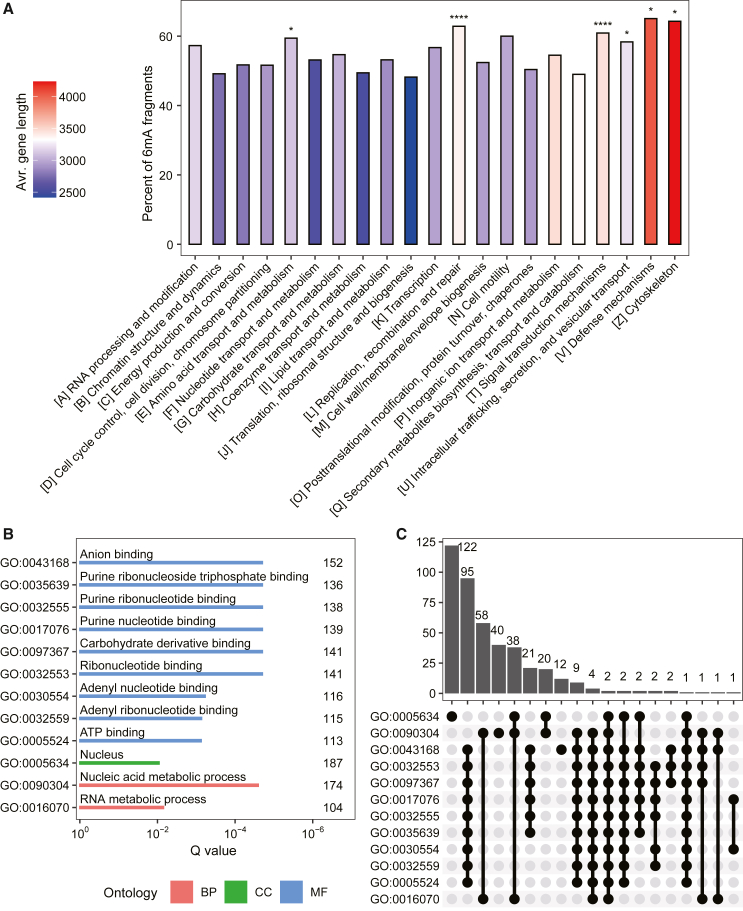
Functional preference of DNA 6mA loci in the *N*. *oceanica* genome **(A)** Percentages of 6mA-methylated fragments for genes in various COG categories. Asterisks indicate significance based on the binomial test (∗*p* < 0.05, ∗∗∗∗*p* < 0.0001). **(B)** Each of the GO terms that are enriched for genes with 6mA marks in their CDS regions. **(C)** Overlaps of genes among the gene sets associated with the GO-term enrichment in **(B)**. A black dot indicates the presence of overlap between or among the gene sets associated with a particular GO enrichment.

We next categorized 6mA-methylated and unmethylated genes using Clusters of Orthologous Genes (COGs) and GO terms. The two COG categories “replication, recombination and repair” and “signal transduction mechanisms” were enriched in 6mA-methylated genes (Figure 2A) (binomial test, adjusted *p* < 0.01). Notably, in the COG categories, the average gene lengths showed strong positive correlations with the ratios of 6mA-methylated genes (Pearson correlation = 0.77; *p* < 0.001). Moreover, the density of 6mA (6mA/A), 0.17% ± 0.02%, was quite similar among the different COG categories, with no outliers detected (Grubbs test, *p* > 0.05).

No GO terms were enriched in genes methylated in the gene body, promoter, exon, 5′ UTR, 3′ UTR, or intron region. However, twelve GO terms were enriched in CDS-methylated genes (false discovery rate [FDR] <0.01). These included the cellular component nucleus (GO:0005634), nucleic acid and RNA metabolic processes (GO:0090304 and GO:0016070), and biological processes related to nucleotide/ribonucleotide binding (GO:0032553, GO:0017076, GO:0030554, and others) (Figure 2B). Comparison of the gene sets associated with these enriched GO terms revealed that nine molecular function GO terms were associated with similar gene sets and shared 112 of the same genes (blue rows; Figure 2C). Thus, for these specific GO categories, marking of 6mA in the CDS region is conserved among selected genes. Nucleotide/ribonucleotide binding and metabolism in the nucleus are fundamental eukaryotic functions. In rice, genes associated with “nucleolus”, “nuclear lumen”, and “nucleotide binding” were enriched in 6mA in gene body regions (Zhou et al., 2018). Thus, the enrichment of these specific functions for CDS-marked genes in *N*. *oceanica* indicates that 6mA may have an ancient origin and is an epigenetic marker shared by eukaryotes.

### Genome-wide positive correlation of 6mA with gene transcription level

**Figure 3:**
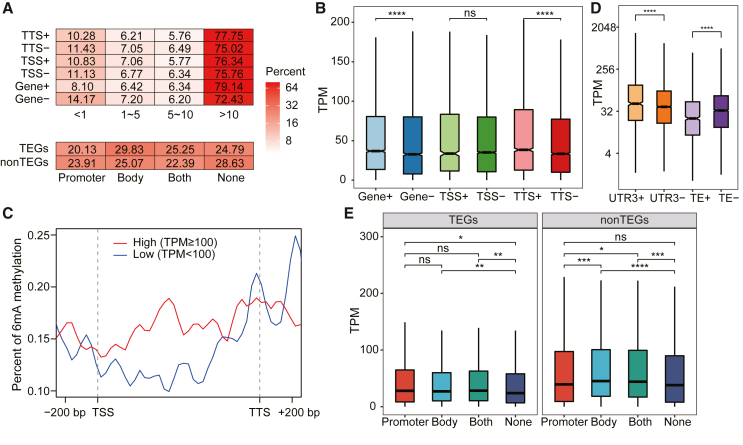
DNA 6mA is associated with gene transcription in *N*. *oceanica* **(A)** Percentages of methylated genes (Gene+), unmethylated genes (Gene–), and genes methylated around the TSS (300-bp upstream and 100-bp downstream; TSS+/TSS–) and TTS (±500 bp; TTS+/TTS–) at a given TPM level (≤1.0, 1–5, 5–10, and ≥10). The number of N6-methylated genes with expression levels below the TPM value of 1.0 (silent genes) was about one-half that of un-methylated silent genes. The percentages of TEGs and non-TEGs with 6mA marks in the promoter, gene body, and both are also shown below. **(B)** Transcription comparison for Gene+, Gene–, and genes N6-methylated near the TSS (TSS+ vs. TSS–) or TTS (TTS+ vs. TTS–). **(C)** 6mA occupancy along genes (strand specific) for highly expressed genes (High, TPM >100) and genes with low expression (Low, TPM ≤100). **(D)** Comparisons between the transcription level of active TEGs (TPM >1) with 6mA-marked TEs (“TE+”) and those without 6mA-marked TEs (“TE–”; “TE+” vs. “TE–”) and active genes (TPM >1) with 6mA-marked 3′ UTRs (“UTR3+”) and those without 6mA in their 3′ UTRs (“UTR3–”; “UTR3+” vs. “UTR3–”). **(E)** Expression levels of genes marked by 6mA only in the promoter, only in the body, or in both compared with the average expression levels of the TEGs (left) or non-TEGs (right). The *y* axis shows the average expression level (TPM). Asterisks indicate significance based on the Wilcoxon test (ns, *p* > 0.05; ∗*p* < 0.05; ∗∗*p* < 0.01; ∗∗∗*p* < 0.001; ∗∗∗∗*p* < 0.0001).

To determine whether and how 6mA regulates gene function, we used RNA sequencing (RNA-seq) to test the genome-wide correlation between 6mA and gene transcription (quantified as transcripts per million [TPM]). The proportion of unmethylated genes with low expression was 75% higher than that of 6mA-methylated genes with low expression (14.2% vs. 8.1%; low expression defined as TPM ≤1.0) (Figure 3A), suggesting an association between the presence of 6mA and active transcription. At the global scale, within the gene body and TTS (defined as ±500 bp), 6mA-methylated genes exhibited higher expression levels than unmethylated genes (Wilcoxon test, *p* < 0.001) (Figure 3B); however, there was no such difference for TSSs (defined as –300 bp to +100 bp) (Figure 3B). Furthermore, scanning the 6mA occupancy along genes and their 3′ UTRs revealed that highly expressed genes (TPM >100) harbored ∼24% more 6mA sites in the gene body than genes with lower expression (TPM <100) (Figure 3C and Supplemental Figure 7). Therefore, 6mA is positively correlated with gene expression levels in *N*. *oceanica*. Notably, such a correlation does not seem to preferentially target any specific functional categories of protein-coding genes, as in each of the COG categories, the expression levels of 6mA-methylated genes and unmethylated genes (at the gene body, TSS, TTS or CDS) showed no significant differences (Wilcoxon test, adjusted *p* > 0.05), i.e., the association between 6mA and transcription is a collective effect of all genes.

The contribution of DNA 6mA accumulation in transposons and 3′ UTRs to active gene expression (TPM >1) was also examined. The transcript levels of active genes with 6mA-marked 3′ UTRs (UTR3+) was significantly higher than that of genes without 6mA in the 3′ UTRs (UTR3–) (Figure 3D) (Wilcoxon test, *p* < 0.0001). This was expected, because the 3′ UTR is part of the gene body and 6mA in the gene body is positively associated with transcription. However, the transcript levels of active TEGs with 6mA-marked TEs (TE+) were significantly lower than those of TEGs without 6mA in TEs (TE–) (Figure 3D) (Wilcoxon test, *p* < 0.0001), similar to results in *Drosophila* (Zhang et al., 2015), implying that 6mA may repress TE activity in *N*. *oceanica*.

Intriguingly, despite the slightly lower 6mA level of TEGs than non-TEGs (Figure 1E), the proportion of TEGs with 6mA-marked promoters or gene bodies was similar to that of non-TEGs (Figure 3A). Moreover, (*i*) TEGs with 6mA marks only in the promoter (Promoter) showed higher average expression levels than un-methylated genes (None) (Wilcoxon test, *p* < 0.05), but this was not true for non-TEGs (Wilcoxon test, *p* > 0.05); (*ii*) for both TEGs (Wilcoxon test, *p* < 0.01) and non-TEGs (Wilcoxon test, *p* < 0.01), genes with 6mA marks in the gene body (Body and Both) showed higher average expression levels than un-methylated genes (None); and (*iii*) for non-TEGs, the Body genes showed higher average expression levels than the Promoter genes (Wilcoxon test, *p* < 0.001) (Figure 3E). Thus, 6mA in the gene body is likely to be the main contributor to activation of gene transcription.

The positive correlation between 6mA in the gene body (both presence and density) and gene transcript level in *N*. *oceanica* is similar to that in rice (Zhou et al., 2018) and is probably conserved in additional evolutionary branches as well. By contrast, 6mA in *C. reinhardtii* is associated with the TSSs of active genes, indicating a role in the regulation of nucleosome positioning around the TSS (Fu et al., 2015). Therefore, considering the enrichment of 6mA around the TTS and 5′ intron regions in *N. oceanica*, 6mA seems to play multiple regulatory roles, not only in transcription termination and co-transcriptional splicing but also in transcript elongation.

### Identification and validation of genes underlying DNA 6mA methylation in *N. oceanica*

**Figure 4:**
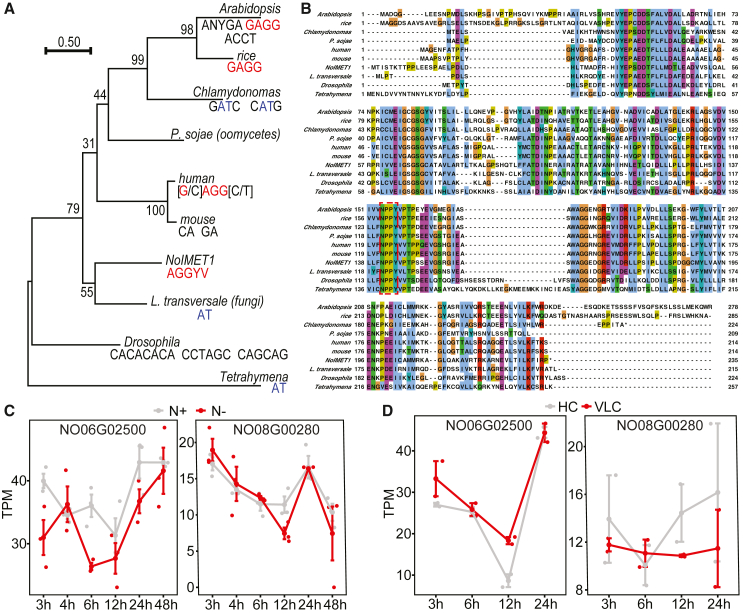
Discovery and validation of enzymes involved in DNA 6mA methylation in *N*. *oceanica* **(A)** Phylogenetic tree of DNA 6mA methyltransferases constructed using MEGA X with the maximum likelihood method. The sequences used include all those present in the alignment in **(B)**. The consensus 6mA motifs identified in each organism are mapped onto the phylogenetic tree. **(B)** Multiple protein sequence alignment of putative DNA 6mA methyltransferases. **(C)** Expression of DNA-6mA-associated genes during nitrogen depletion. The DNA 6mA methyltransferase NO08G00280 was downregulated at 48 h. **(D)** Expression of DNA-6mA-associated genes during CO_2_ depletion. Expression of the DNA 6mA demethylase NO06G02500 continued to decline at 6–12 h of CO_2_ depletion, but that of the DNA 6mA methyltransferase of NO08G00280 had begun to increase by that time frame.

To pinpoint the functional mechanism of DNA 6mA methylation in *Nannochloropsis* spp., we identified candidate genes encoding a 6mA “writer” and “eraser”. We proposed the *N. oceanica* gene NO08G00280, which encodes an S-adenosyl-L-methionine-dependent methyltransferase, as a candidate DNA 6mA methyltransferase gene because of its homology to human N6AMT1, which was functionally validated as a DNA 6mA methyltransferase in human (Xiao et al., 2018). A phylogenetic analysis of putative and validated N6AMT1 enzymes from various organisms, including *Nannochloropsis*, *Chlamydomonas*, *Arabidopsis*, and human, revealed significant conservation of their protein sequences (Figure 4A, 4B, and Supplemental Table 5). However, this analysis was unable to establish a clear link between N6AMT1 and observed 6mA motifs (Figures 4A and 4B). These results suggested that N6AMT1 may not be the primary contributor to 6mA methylation in most organisms. Nonetheless, the presence of a similar GAGG-like 6mA motif in *N*. *oceanica*, human, and plants suggested that N6AMT1 and, in this case NO08G00280, was a promising 6mA methylase candidate.

We further hypothesized that the AlkB family gene NO06G02500 was involved in 6mA demethylation, as its encoded protein is a homolog of rice OsALKBH1 (Zhou et al., 2018) and human ALKBH1 (Liu et al., 2016; Xiao et al., 2018; Zhang et al., 2020), which have been validated as 6mA demethylases by *in vivo* and *in vitro* experiments. In time-series transcriptomic profiles of *N*. *oceanica* under nitrogen depletion (Li et al., 2014) and CO_2_ depletion (Wei et al., 2019), NO08G00280 and NO06G02500 transcripts both exhibited constitutive expression, but their temporal patterns were distinct (Figures 4C and 4D). In general, expression of both genes first decreased and then increased under stress. However, under nitrogen depletion (N+/N–), NO08G00280 expression decreased further at 48 h (Figure 4C). By contrast, under CO_2_ depletion (HC/VLC), NO08G00280 expression increased from 6 h onwards, but NO06G02500 expression increased only after 12 h (Figure 4D). These observations were consistent with potential roles for NO08G00280 and NO06G02500 in regulating DNA 6mA methylation in response to stress.

**Figure 5:**
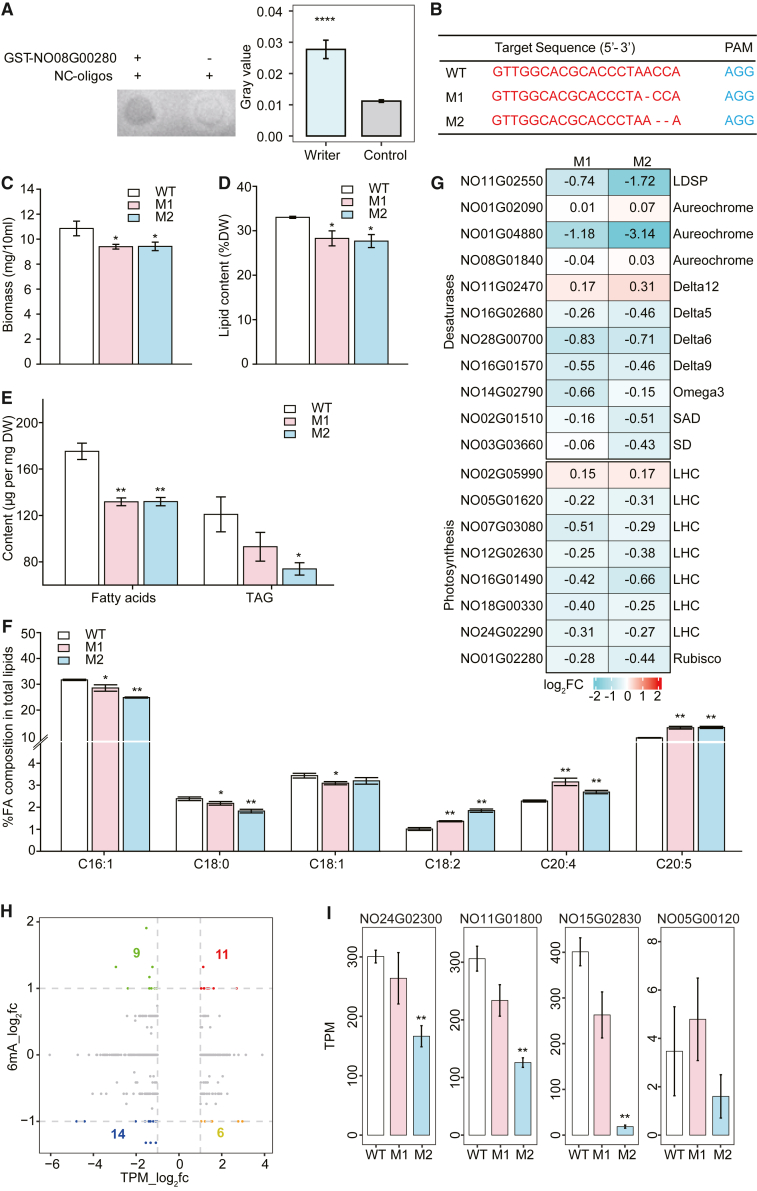
Changes in *N*. *oceanica* phenotypes resulting from knockout of NO08G00280, a putative DNA 6mA methyltransferase **(A)** The recombinant GST-NO08G00280 protein slightly methylated DNA oligos in an *in vitro* methylation reaction as revealed by dot blotting assays. The gray values of randomly selected reaction areas were compared (Wilcoxon test, ∗∗∗∗*p* < 0.0001). **(B–F)** Mutant genome sequences at the gRNA target sites. Phenotypic changes under high-light conditions are shown: biomass productivity **(C)**, lipid content **(D)**, fatty acid and TAG contents **(E)**, and fatty acid composition of total lipids **(F)**. **(G)** Transcription of selected genes associated with photosynthesis, CO_2_ fixation, FA synthesis, desaturases, etc. (RNA-seq experiments in triplicate). Log2-fold changes in expression are shown in the heat map (red, upregulation; green, downregulation). **(H)** The link between differential gene expression (TPM, log2 scale) and 6mA-level dynamics (6mA/A, log2 scale) in WT and mutants. For WT strains, WT_2 and WT_3 were used to calculate the 6mA level; for mutants, M2_1 and M2_2 were used. **(I)** Expression levels of selected genes in the WT and mutants (TPM values). Error bars denote mean ± SD (in triplicate). Asterisks indicate significance based on the *t*-test (∗*p* < 0.05, ∗∗*p* < 0.01).

To test the role of NO08G00280 as a DNA 6mA methyltransferase, we investigated its effect on the 6mA level of *N*. *oceanica* genomic DNA *in vitro* and *in vivo*. For the *in vitro* test, the cDNA of NO08G00280 was ligated into the pGEX plasmid, and recombinant GST-NO08G00280 was purified. In an *in vitro* methylation assay, a synthesized NC-oligo (Xiao et al., 2018) was methylated by the purified recombinant GST-NO08G00280, and the methylated DNA was detected and quantified by dot blotting with an anti-6mA antibody. The dot blot result showed that GST-NO08G00280 increased the 6mA level of synthetic oligonucleotide substrates (NC-oligo) by ∼1.5-fold (Xiao et al., 2018) (Figure 5A). Notably, the dA methylation activity of NO08G00280 was not particularly high, consistent with the low abundance of 6mA in *N*. *oceanica*. To test its role as a DNA 6mA methyltransferase *in vivo*, NO08G00280 was knocked out by CRISPR–Cas9 to produce mutants M1 and M2 (Figure 5B). High light, which enhances lipid accumulation in wild-type (WT) *Nannochloropsis* spp. (Alboresi et al., 2016; Ma et al., 2016; Huete-Ortega et al., 2018; Han et al., 2020), was used to test potential links among the knockout genotype, altered 6mA patterns, and changes in gene expression. After 7 days of high-light culture, three replicate samples of M1, M2, and WT *N. oceanica* were obtained, and genome-wide 6mA events were profiled using the PacBio Sequel II platform (>100× sequencing depth per sample).

The mutants and WT had equivalent global 6mA levels, indicating the presence of additional DNA 6mA methyltransferases in *N*. *oceanica* (Supplemental Figure 8). However, comparison between M2 and WT revealed that 19.5% of the 6mA-marked genes (i.e., those with at least one 6mA site in all replicates of M2 or WT; 225 of 1152) were shared between M2 and WT; 15.6% of them showed changes in 6mA levels (fold change >2) in M2 relative to WT, with 7.8% showing increased 6mA levels. In addition, 80.5% of the 6mA-marked genes changed methylation state from carrying at least one 6mA site to none (WT to M2, 43.7%) or vice versa (WT to M2, 36.8%). No significant differences in the 6mA distribution pattern along genes were detected between M2 and WT (Supplemental Figure 8). These observations suggest a large-scale alteration of genome-wide 6mA patterns due to knockout of NO08G00280.

In terms of the microalgal phenotype, mutant growth (biomass accumulation) was 13% lower than WT growth (*t*-test, *p* < 0.05) (Figure 5C). Similarly, lipid content was 14.3% lower in M1 and 16.2% lower in M2 (Figure 5D) than in the WT. Gas chromatography–mass spectrometry (GC–MS) revealed that the fatty acid content was 24.8% lower in M1 and 24.7% lower in M2, and triacylglycerol (TAG) content was 26.8% lower in M2 (Figure 5E). However, the percentage of polyunsaturated fatty acids (PUFAs) was significantly higher in the mutants than in the WT (34.4% and 81.4% increases in C18:2, 38.1% and 17.8% increases in C20:4, and 42.7% and 44.0% increases in C20:5 in M1 and M2, respectively) (Figure 5F). Thus, 6mA is important for growth and lipid metabolism in *N*. *oceanica*.

To probe the underlying mechanism, RNA-seq for the WT and mutants was performed for the same samples in biological triplicates (which showed excellent reproducibility) (Supplemental Figure 9). Compared with the WT, the NO08G00280-knockout mutants (M1 or M2) contained 600 differentially expressed genes (DEGs) (Supplemental File 1). Specifically, expression levels of Rubisco (Vieler et al., 2012) and almost all light-harvesting complex (LHC) genes were downregulated in M1 and M2 (Figure 5G) (Supplemental File 1), consistent with the slower growth phenotype. Expression of the lipid droplet (LD) surface protein gene NO11G02550, which encodes a major LD-associated protein that serves as a marker of TAG accumulation and LD dynamics in *N*. *oceanica* (Vieler et al., 2012; Zienkiewicz et al., 2020), was also downregulated in M1 and M2, consistent with the phenotype of reduced TAG contents. Also downregulated was NO01G04880, which encodes an aureochrome, a stramenopile-specific transcription factor with a bZIP DNA-binding motif (Takahashi et al., 2007). We recently showed that another *N*. *oceanica* aureochrome (NO08G01840) is a blue light–responsive transcription factor that modulates lipid production by repressing NoDGAT2B transcription (Zhang et al., 2022). Notably, the reduction in NO01G04880 transcripts (log2-fold change) (Figure 5G) was consistent with the reduction in TAG content in the mutants (Figure 5E), suggesting a potential link between NO01G04880 and TAG production as well.

Comparison of 6mA dynamics (6mA/A, log2 scale) with differential gene expression levels (TPM, log2 scale) further supported the positive correlation between 6mA levels and gene expression in *N*. *oceanica*. Specifically, (*i*) among the upregulated DEGs, those with a >2-fold change in 6mA outnumbered those with a <0.5-fold change in 6mA (11 vs. 6), and (*ii*) among the downregulated DEGs, those with a <0.5-fold change in 6mA outnumbered those with a >2-fold change in 6mA (14 vs. 9) (Figure 5H).

In addition, a reduction in 6mA level was observed for many downregulated genes, e.g., NO24G02300, NO11G01800, NO15G02830, and NO05G00120 (Figure 5I) (NO08G00280-knockout mutants vs. WT). NO24G02300 encodes a protein involved in biosynthesis of the molybdenum cofactor, a compound present at the active site of many molybdenum-containing enzymes such as nitrate reductase (NR), sulfite oxidase, xanthine oxidoreductase, and aldehyde oxidase (Tejada-Jimenez et al., 2018). Indeed, we observed a strong positive correlation (Pearson correlation coefficient, R = 0.72) between transcript abundance of NO24G02300 and that of the NR gene (NO14G02460) (dropping by half for both genes; NO08G00280-knockout strains vs. WT). The reduction in NO24G02300 transcription in the mutants may therefore hinder nitrogen assimilation, helping to explain their slower growth. NO11G01800 encodes a sulfate transporter, and its transcriptional inhibition due to a reduced 6mA level may limit sulfur fixation in this microalga, also contributing to slower growth (Dong et al., 2017). NO15G02830 encodes a glycosyl transferase with a UDP-sulfoquinovose:DAG sulfoquinovosyltransferase domain (PLN02871) that participates in synthesis of sulfoquinovosyl diacylglycerols (SQDGs; sulfur-containing lipids and major components of the microalgal thylakoid membrane) (Merchant and Helmann, 2012). Thus, the reduction in NO15G02830 transcription associated with reduced 6mA modification may explain the reduction in lipids. NO05G00120 encodes a lipase, which hydrolyzes the ester bonds of lipids with a substrate preference (Long et al., 1998; Chen et al., 2020; Jithu Paul, 2020). Among the ∼40 candidate lipase genes in *N*. *gaditana*, at least 6 are thought to mediate translocation of EPA from polar membrane lipids to TAG during nitrogen starvation (Janssen et al., 2020). Notably, NO05G00120 was upregulated in *N*. *oceanica* at 12 h of nitrogen starvation (Li et al., 2014), consistent with its likely role in liberating PUFA-rich membrane lipids. Therefore, the downregulation of NO05G00120 transcripts, correlated with reduced 6mA modification, could explain the increased PUFA content in the NO08G00280-knockout strains.

In summary, knockout of the candidate DNA 6mA methyltransferase NO08G00280 resulted in large-scale alterations in 6mA levels and a cascade of transcriptomic changes. Specifically, downregulation of NO24G02300, NO11G01800, NO15G02830, and NO05G00120, which was correlated with their reduced 6mA modification, was linked to a reduction in microalgal growth and lipid accumulation. This evidence supports the identification of NO08G00280 as a writer that introduces genome-wide 6mA modifications in *N*. *oceanica*.

### Identification and validation of a DNA 6mA demethylation gene in *N. oceanica*

**Figure 6:**
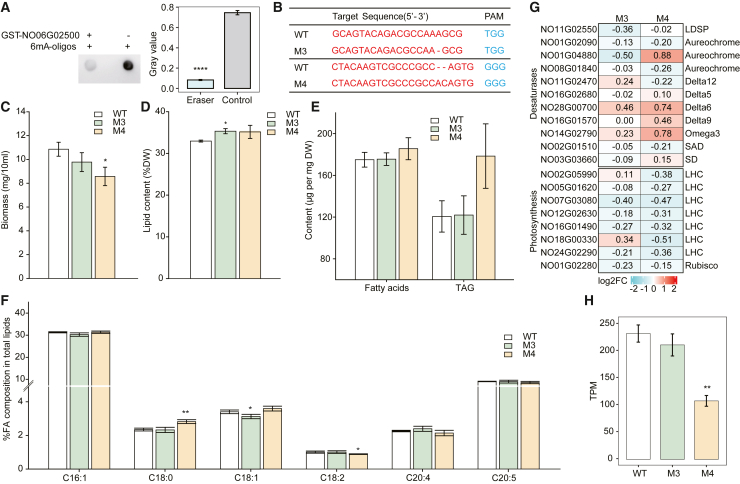
Changes in *N*. *oceanica* phenotypes resulting from knockout of NO06G002500, a putative DNA 6mA demethylase **(A)** The recombinant GST-NO06G002500 protein directly and efficiently demethylated the 6mA modification in an *in vitro* demethylation reaction with 6mA-DNA oligos as the substrate. The gray values of randomly selected reaction areas were compared (Wilcoxon test; ∗∗∗∗*p* < 0.0001). **(B)** Mutant genome sequences at the gRNA target sites. Phenotypic changes under high-light conditions are shown: biomass productivity **(C)**, lipid content **(D)**, fatty acid and TAG contents **(E)**, and fatty acid composition of total lipids **(F)**. **(G)** Transcription of selected genes associated with photosynthesis, CO_2_ fixation, FA synthesis, desaturases, etc. (RNA-seq experiments in triplicate). Log2-fold changes in expression are shown in the heat map (red, upregulation; green, downregulation). **(H)** Expression levels of selected genes in the WT and mutants (TPM values). Error bars denote mean ± SD (in triplicate). Asterisks indicate significance based on the *t*-test (∗*p* < 0.05, ∗∗*p* < 0.01).

To verify the functional activity of the candidate DNA 6mA demethylase, we investigated whether NO06G02500 could demethylate 6mA from DNA *in vitro* and *in vivo*. The cDNA of NO06G02500 was ligated into the pGEX plasmid, and recombinant GST-NO06G02500 was purified. In an *in vitro* demethylation assay, synthesized 6mA-oligos (Xiao et al., 2018) were demethylated by the purified recombinant GST-NO06G02500, and the reduction in methylated DNA due to demethylase activity was detected and quantified by dot blotting. The results showed that GST-NO06G02500 could directly and efficiently reduce the 6mA level of synthetic 6mA-modified oligonucleotide substrates (6mA-oligo) (Xiao et al., 2018) (Figure 6A).

To test the role of the NO06G02500 protein as a DNA 6mA demethylase *in vivo*, the NO06G02500 gene was knocked out by CRISPR–Cas9 to create the M3 and M4 mutants (Figure 6B). After 7 days of high-light culture, replicate M3, M4, and WT plants were obtained, and genome-wide 6mA events were profiled by PacBio Sequel II sequencing (>100× sequencing depth per sample). Consistent with the proposed demethylase activity of NO06G02500, global 6mA levels for both knockout mutants were increased by 21.3% (*p* < 0.05, one-tailed Wilcoxon test) (Supplemental Figure 8). Specifically, 20.9% of the 6mA-marked genes (i.e., those with at least one 6mA site in all replicates of M4 or WT; 306 out of 1464) were shared between M4 and WT; 20.3% of them showed changes in 6mA levels (fold-change >2) in M4 relative to WT, with 13.7% showing increased 6mA levels. In addition, 79.1% of the 6mA-marked genes changed methylation state from carrying at least one 6mA site to none (WT to M4, 28.8%) or vice versa (WT to M4, 50.3%). The distribution pattern of 6mA density along genes was similar in M4 and WT, but the overall 6mA density was higher in M4 (Supplemental Figure 8).

In terms of phenotype, growth rates of M3 and M4 were 10% and 21% lower than that of the WT (Figure 6C) (*t*-test, *p* < 0.05), and lipid contents were 7.2% and 6.7% higher (Figure 6D) (*t*-test, *p* < 0.05). However, there were no significant differences between M3 or M4 and the WT in fatty acid content, TAG content, or PUFA composition (except for C18:2 which was 11.8% lower in M4) (Figure 6E and 6F). The corresponding RNA-seq data revealed that 79 genes were significantly differentially expressed in M3 or M4 compared with the WT (Supplemental File 1), 46 of which were upregulated (58.2% of DEGs).

The dynamics of 6mA modification and changes in transcript abundance were overlayed to examine their links. Expression of Rubisco and nearly all LHC genes was downregulated in M3 and M4 (Figure 6G; Supplemental File 1); consistent with this result, the 6mA levels of these genes were reduced or remained unchanged. Both 6mA level and transcript abundance were reduced for a peptide methionine sulfoxide reductase gene (NoMSR; NO21G00950) that functions in protein repair and oxidative damage protection (Tarrago et al., 2009). The downregulation of NoMSR in M3 (fold change = 0.87) and M4 (fold change = 0.47; Figure 6H) may have aggravated the level of unrepaired proteins (with changes in activity or conformation), which may have contributed to the slow growth of the mutants.

In summary, knockout of the candidate DNA 6mA demethylase NO06G02500 in *N*. *oceanica* resulted in a significant increase in global 6mA levels (by 21.3%), which was accompanied by growth defects. However, transcripts with changes in 6mA could be either up- or downregulated, underscoring the complexity of NO06G02500-mediated demethylation in the regulation of gene expression.

### A model for epigenomic regulation of lipid accumulation in *N. oceanica*

**Figure 7:**
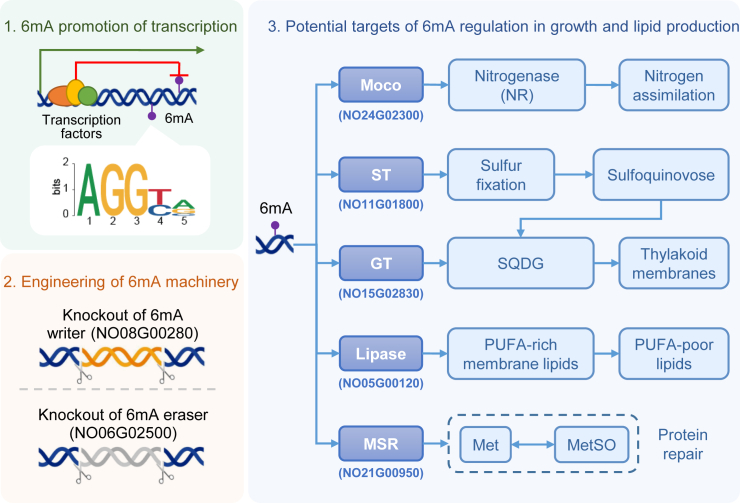
A mechanistic model for 6mA-mediated regulation of lipid metabolism in *Nannochloropsis* spp. The 6mA-mediated regulation of oil production has multiple aspects, as demonstrated by the phenotypes of knockout mutants of a methyltransferase and a demethylase in the DNA 6mA machinery. These aspects include mediation of nutrient uptake and assimilation (such as nitrogen and sulfur; Moco and ST), modulation of membrane lipid composition (GT), and regulation of specific core genes (MSR). GT, glycosyl transferase; Moco, molybdenum cofactor; Met, methionine; MetSO, methionine sulfoxide; MSR, methionine sulphoxide reductase; ST, sulfate transporter.

Genome-wide profiling of 6mA sites and genetic engineering of DNA-modification machinery enabled us to propose an epigenetic layer of regulation for lipid production in *N*. *oceanica* (Figure 7). On the basis of the positional and functional preference of 6mA localization, we showed that 6mA can promote gene transcription, perhaps by facilitating the recruitment of general transcription factors. Moreover, knockout of a methyltransferase and a demethylase in the DNA 6mA machinery disturbed the transcription of multiple genes associated with lipid production and significantly altered growth and lipid accumulation.

In this model, the epigenetic regulatory layer of oil production has three aspects: (*i*) 6mA may regulate the expression of several genes that mediate uptake and assimilation of nutrients (such as nitrogen and sulfur; NO24G02300 and NO11G01800), which not only are essential elements of the building blocks of lipids such as phospholipids, SQDGs, etc., but also affect cell growth. (*ii*) 6mA may regulate the expression of genes involved in molding the composition of membrane lipids (NO15G02830), thus further affecting the function of subcellular compartments. For example, the abundance of SQDGs in thylakoid membranes would affect cell photosynthesis, thereby linking lipid accumulation to cell growth; moreover, some lipases can mediate the conversion between membrane lipids and free neutral lipids, thus altering the properties of organelle membranes. (*iii*) 6mA may regulate certain core genes that constitute the foundation of lipid accumulation. For instance, the core gene NoMSR mediates the maintenance of protein activity, and compromised regulation by 6mA could lead to growth defects. This model therefore provides potential mechanistic links between 6mA-based transcriptional regulation and lipid production in *N*. *oceanica*.

## Discussion

A correlation between 6mA modification and gene transcription is found in eukaryotic organisms including *C*. *reinhardtii* (Fu et al., 2015), *A*. *thaliana* (Zhe et al., 2018), rice (Zhang et al., 2018; Zhou et al., 2018), and mouse (Wu et al., 2016). In *C*. *reinhardtii*, 6mA affects nucleosome positioning around the TSS and thus contributes to transcription initiation (Fu et al., 2015). In *N*. *oceanica*, after excluding the possibility of the results being skewed by bacterial contamination (Supplemental results; Supplemental Table 6), we showed that 6mA genomic distribution, enriched motifs, and association with transcription in *N. oceanica* are highly distinct from those in *C*. *reinhardtii* and higher plants. Enrichment of 6mA around the 5′ intron and TTS suggests roles in co-transcriptional splicing and transcription termination in *N*. *oceanica*. Our data support the hypothesis that 6mA regulates gene transcription by influencing the recruitment/binding of transcription-related factors (Zhe et al., 2018).

Comparison of *N*. *oceanica* with *C*. *reinhardtii* sheds light on the evolutionary conservation and the functional significance of 6mA in algae (Supplemental Table 7). In general, the 6mA level is much lower in *N. oceanica* than in *C*. *reinhardtii*, and their 6mA distribution patterns vary markedly. In *C*. *reinhardtii*, 6mA mainly resides at ApT dinucleotides around the TSS, with a bimodal distribution, and it appears to mark active genes (Fu et al., 2015). By contrast, 6mA sites in *N. oceanica* are non-canonical, mainly enriched at AGGYV motifs and not around TSSs. The most striking feature observed in *N*. *oceanica* was that highly expressed genes had higher 6mA levels in the gene body, a pattern distinct from that in *C*. *reinhardtii* but similar to that in plants. Thus, 6mA has evolved distinct functions between stramenopiles and green algae.

A comparison of 6mA in *N*. *oceanica* and plants also yields intriguing insights. The non-canonical localization of 6mA sites and the enrichment mostly at AGGYV motifs in *N*. *oceanica* are similar to those in plants (*A*. *thaliana* and rice). However, in contrast to observations in plants, 6mA sites are not enriched around the TSS in *N. oceanica*, and 6mA/A is higher in introns than in exons. In addition, although highly expressed genes have higher 6mA levels in the gene body in both *N*. *oceanica* and plants, there is no indication that 6mA in promoters marks silent genes in *N*. *oceanica*. Notably, despite such differences in distribution patterns among organisms, DNA 6mA is associated with active transcription for most organisms.

Knockout of the candidate methyltransferase NO08G00280 and the candidate demethylase NO06G02500 via CRISPR–Cas9 altered 6mA levels in *N*. *oceanica*. In the NO08G00280-knockout mutants, the loss of 6mA was correlated with decreased gene expression, further supporting the association between 6mA and actively expressed genes. Consistent with the slow growth and altered lipid profiles of the mutants, NO08G00280 knockout led to reduced 6mA levels at the key genes NO24G02300, NO11G01800, NO15G02830, and NO14G02460, which were accompanied by reduced gene expression. Thus, 6mA plays an important role in growth and lipid metabolism of *N*. *oceanica*. However, the 6mA level increased only slightly (by 21.3%) in NO06G02500-knockout mutants, likely owing to the relatively short cell division cycle of microalgae (compared with higher plants). Moreover, ∼65% of the DEGs still have no functional annotations (Supplemental File 1), underscoring the challenge in untangling the mechanisms behind the mutant phenotypes. The mechanistic links between 6mA and transcriptional regulation of lipid/growth-related genes, which to date derive mainly from multi-omics associations, should be investigated further by additional biochemical or genetic experiments.

Epigenetic manipulation of algal genomes holds untapped potential for increasing biofuel productivity (Steadman et al., 2020). Epigenetic proteins are druggable targets that can be addressed through small-molecule inhibitors. For example, in the microalga *Picochlorum soloecismus*, inhibition of DNA methylation using 5-aza-20-deoxycytidine increases cell size and lipid accumulation (Steadman et al., 2020). Moreover, the epigenome can be manipulated in a locus-specific manner via CRISPR–dCas9/12a (Sgro and Blancafort, 2020). Therefore, the genome-wide 6mA map and identification of key 6mA machineries reported here for the model oleaginous microalga *N*. *oceanica* pave the way for epigenetic engineering of superior algal feedstocks that can directly convert carbon dioxide to oils.

## Methods

### Cultivation and SMRT sequencing of WT *N. oceanica* for construction of a genome-wide 6mA map

*N*. *oceanica* IMET1 was inoculated into modified f/2 liquid medium, which was prepared with 35 g L^−1^ sea salt, 1 g L^−1^ NaNO_3_, 67 mg L^−1^ NaH_2_PO_4_·H_2_O, 3.65 mg L^−1^ FeCl_3_·6H_2_O, 4.37 mg L^−1^ Na_2_EDTA·2H_2_O, trace metal mix (0.0196 mg L^−1^ CuSO_4_·5H_2_O, 0.0126 mg L^−1^ NaMoO_4_·2H_2_O, 0.044 mg L^−1^ ZnSO_4_·7H_2_O, 0.01 mg L^−1^ CoCl_2_, and 0.36 mg L^−1^ MnCl_2_·4H_2_O), and vitamin mix (2.5 μg L^−1^ vitamin B_12_, 2.5 μg L^−1^ biotin, and 0.5 μg L^−1^ thiamine HCl). The algal cells were grown in liquid cultures under continuous light (∼50 μmol photons m^−2^ s^−1^) at 25°C and aerated by bubbling with a mixture of 1.5% CO_2_ in air.

Cells were collected by centrifugation and used for genomic DNA isolation. Cultures were pelleted, and nucleic acids were extracted using phenol–chloroform and treated with RNase to degrade RNA. gDNA (5–10 μg) was sheared to >10 kb using g-TUBEs (Covaris). The sheared DNA was treated with DNA damage repair mix, followed by end repair and ligation of SMRT hairpin adapters using the SMRTbell Template Preparation Reagent Kit (Pacific Biosciences). Fragments without adaptors were digested with exonuclease. Libraries were sequenced on a Pacific Biosciences RS-II sequencer using standard protocols at the DOE Joint Genome Institute.

The PacBio SMRT analysis platform (version: 2.3.0.140936.p4) was used to detect DNA 6mA modifications. In brief, the subreads were filtered based on strict criteria (minimum read length of 100, read score of 0.8); the filtered reads were then aligned to the reference genome using pbalign (version: 0.2.0) with strict parameters (“--minAccuracy=0.75 --minLength=100 --concordant”); finally, kinetic analysis of the aligned subreads was used to identify DNA 6mA modifications with kineticsTools (Clark et al., 2012) in the “P_ModificationDetection” module. DNA 6mA modifications were extracted from the identified base modifications.

### Computational mapping of the genome-wide distribution of DNA 6mA sites

DREME (from MEME suite v5.0.2) (Bailey, 2011) was used to find relatively enriched motifs in the two flanking 4-bp sequences of 6mA sites (9mers), with all candidate sequences (both strands) in the genome as controls and the parameters “-norc -mink 2 -maxk 5”. Densely methylated adenine clusters were defined as at least 10 adjacent 6mA sites (with intervals <100 bp).

We defined the genome-wide N6-methylation level of adenine sites as the mean of 6mA sites from all adenine sites (strand specifically if not specified). The ratio of methylation for each adenine site cannot be reliably determined and was thus not taken into consideration. Analyses were strand specific unless otherwise indicated (several non-strand-specific analyses were also performed, with results shown in the Supplemental information). The percentage of methylation sites in each gene category was defined as above after considering the non-strand-specificity of intergenic regions. A fragment/gene was designated as methylated if any adenine it contained was N6-methylated. DNA 6mA distribution around the gene body, TSS, and TTS was calculated along the forward strand of the gene and drawn after smoothing (locally weighted scatterplot smoothing regression with f = 0.05). For a fair comparison, distribution of the 6mA ratio along the gene body (or intron) was consolidated into 1000 bp (or 200 bp) by uniform sampling or interpolation.

GO enrichment of *N*. *oceanica* genes with 6mA modifications was analyzed using the R package clusterProfiler (Wu et al., 2021) with a Q-value cutoff of 0.01. For cross-organism comparison, public datasets were retrieved from the Gene Expression Omnibus (GEO) database (*Chlamydomonas reinhardtii*, GSE 68860; *Arabidopsis thaliana*, GSM 2157793; *Oryza sativa*, GSE 103145).

### Measurement of 6mA/A ratio by LC–MS/MS

*N*. *oceanica* 6mA and dA were profiled via LC–MS/MS (Allwegene Technologies Inc.). The extracted *N*. *oceanica* genomic DNA was first digested by the Dpn I restriction enzyme, then subjected to ultrafiltration to remove possible bacterial DNA contamination. DNA (dissolved in water) was first denatured by heating at 95°C for 5 min and then chilling on ice for 2 min. After addition of S1 nuclease buffer, alkaline phosphatase, and DNase I (Takara Biotechnology), the mixture was incubated at 37°C. After the DNA was completely digested into nucleosides, the mixture was extracted with chloroform. The resulting aqueous layer was collected, reconstituted in water, and analyzed by LC–electrospray ionization–MS/MS.

### Dot blotting for DNA 6mA

Dot blotting was performed as described previously with minor modifications (Zhang et al., 2015). In brief, the synthesized oligos (Wu et al., 2016) were loaded on an HATF00010 nitrocellulose membrane (Merck Millipore) and air dried for 5 min. The membrane was baked at 80°C for 2 h and then blocked in blocking buffer (5% milk in PBST) for 2 h at room temperature. The membrane was incubated with a specific anti-6mA antibody (Synaptic systems; 1:2000) overnight at 4°C, then incubated with horseradish peroxidase-conjugated anti-rabbit IgG secondary antibody M21003 (Abmart; 1:2000) at room temperature for 1.5 h. The antibody-bound 6mA was then incubated with a high-sensitivity enhanced chemiluminescence reagent (Sangon Biotech) and detected and quantified with a FUSION Solo 6S imaging system (VILBER).

### 6mA methylation assays *in vitro*

*In vitro* methylation reactions were performed as described previously with minor modifications (Xiao et al., 2018). In brief, the reactions were performed in a 25-μL methylation reaction buffer containing 250 pmol DNA NC-oligos, 800 ng recombinant GST-NO08G00280 protein, 50 mM Tris–HCl (pH 7.6), 50 mM KCl, 10 mM Mg(OAc)_2_, 7 mM β-mercaptoethanol, 800 mM S-adenosylmethionine, and 100 μg/mL bovine serum albumin. Reactions were carried out overnight at 25°C. DNA was purified with a DP214 DNA purification kit (Tiangen Biotech), and the purified DNA was used for 6mA dot blotting. The NC-oligos used are listed in Supplemental Table 8.

### 6mA demethylation assays *in vitro*

*In vitro* demethylation reactions were performed as described previously with minor modifications (Wu et al., 2016). In brief, the reactions were performed in a 50-mL demethylation reaction buffer containing 50 pmol 6mA-oligos, 230 ng recombinant GST-NO06G02500 protein, 50 mM HEPES (pH 7.0), 50 mM KCl, 1 mM MgCl_2_, 2 mM ascorbic acid, 1 mM a-KG, and 1 mM (NH_4_)_2_Fe(SO_4_)_2_·6H_2_O. Reactions were carried out for 1 h at 37°C, and 2 mL of reaction product was used for dot blotting. The 6mA-oligos used are listed in Supplemental Table 8.

### CRISPR-based knockout of NO08G00280 and NO06G02500

For CRISPR-based targeted knockout in *N*. *oceanica* (Wang et al., 2016), a modular CRISPR–Cas9 toolbox system (pNOC-ARS-CRISPR) was used to construct the CRISPR plasmids (Poliner et al., 2018). Specifically, guide RNAs (gRNAs) were designed using the CHOPCHOP platform (http://chopchop.cbu.uib.no). For each gRNA, a pair of synthesized oligonucleotides named Target-3230-gRNA F and R were annealed to form a dimer with overhangs at both ends. Each dimer was ligated with the BspQI-digested plasmid pNOC-ARS-CRISPR to generate an entry clone with the full-length gRNA. The linearized vectors (containing a bleomycin resistance gene) with *AseI* digestion were introduced into the microalgae by electroporation (Li et al., 2014). For transformant selection, electroporated cells were plated onto 50% fresh seawater agar plates (1% agar) supplemented with 5 μg mL^−1^ zeocin (Invitrogen). After 2–3 weeks of incubation in white light (ca. 100 μmol m^−2^ s^−1^) at 20°C, individual resistant colonies were inoculated into liquid f/2 medium with 5 μg mL^−1^ zeocin. The transformants were screened by checking for integration of the ShBle gene with the primers ble_fw and ble_rv.

### Comparing the global 6mA maps of *N. oceanica* WT and mutants by SMRT sequencing

For both WT and mutants, microalgal cells were cultivated in a glass column (25 cm height × 3.5 cm diameter) under high light (150 ± 20 μmol photons m^−2^ s^−1^) with an initial OD_750_ = 0.5 and bubbling with air. The samples were collected at 7 days for preparation of DNA and RNA libraries. *N*. *oceanica* cells were harvested by centrifugation for 5 min at 2500 × *g*, then immediately quenched with liquid N_2_ and stored in a –80°C freezer.

DNA libraries for SMRT sequencing were constructed with an insert size of 20 kb using the SMRTbell Express Template Prep Kit 2.0, then sequenced on the PacBio Sequel II platform (Sequel II Sequencing Kit 2.0; running in CLR mode) at Novogene Biotech Co., Ltd.

PacBio SMRT Link (v10.1.0) was used for the resequencing and base modification analysis. The resequencing module was used to map subreads to the reference genome (with minimum mapped length set to 200 bp) and check variant information at the mutation sites. Then, datasets with sufficient sequencing depth, including two replicates of WT (WT_2 and WT_3), one replicate of M1 (M1_3), two replicates of M2 (M2_1 and M2_2), three replicates of M3 (M3_1, M3_2, and M3_3), and two replicates of M4 (M4_1 and M4_2), were subsampled to 100× (with a genome size of 31 Mb) from the mappable subreads. To detect DNA 6mA modification, the Base Modification Analysis module was used with a minimum mapped length of 200 bp and a minimum methylated fraction of 0.1. Finally, DNA 6mA modifications were extracted from the identified base modifications.

### RNA-seq library construction for WT and mutant lines

Total algal RNA was extracted using TRIzol reagent (Tiangen). The concentration and purity of the RNA were determined spectrophotometrically (IMPLEN), and RNA integrity was assessed using the RNA Nano 6000 Assay Kit with the Agilent Bioanalyzer 2100. A total of 2 μg RNA per sample was used as input material for RNA sample preparation. Sequencing libraries were generated using the NEBNext Ultra RNA Library Prep Kit for Illumina (New England Biolabs) following the manufacturer’s recommendations, and index codes were added to attribute sequences to each sample. Clustering of the index-coded samples was performed on a cBot Cluster Generation System using the HiSeq 3000/4000 PE Cluster Kit Box1 from Illumina. After cluster generation, the libraries were sequenced on the Illumina HiSeq 4000 platform, and 150-bp paired-end reads were generated.

### Computational analysis of RNA-seq data

RNA-seq datasets were processed using the nfcore/rnaseq pipeline (https://nf-co.re/rnaseq), and reads were aligned using STAR with modified parameters to limit intron lengths (--alignIntronMin 20 --alignIntronMax 3000). Trinity (Grabherr et al., 2011) was then used to generate a gene expression matrix with the RSEM2 method and TMM normalization to account for variation in library size between samples. TPM values (Li and Dewey, 2011; Wagner et al., 2012) were averaged among replicates. For mutants, differentially expressed genes were identified using edgeR (Robinson et al., 2010) with an FDR of ≤0.001 and a minimum fold change of >2. Differences in expression between genes with and without 6mA modifications were determined on the basis of TPM values. A Wilcoxon test was performed to assess the statistical significance of TPMs in different categories.

### Phenotyping of WT and mutant lines

For strain phenotyping, 10 mL of cells were collected by centrifugation after 7 days of culture and dried by vacuum freezing for at least 15 h before dry weight measurement. The lyophilized algal powder (5–10 mg) was stirred with 2 mL chloroform:methanol (2:1, v/v) for 1 h at 30°C. The extract was mixed with 1 mL 0.7% KCl and centrifuged at 1500 × *g* for 10 min; the lower organic layer was transferred to a new vial. The organic layer was evaporated under nitrogen gas and dried in a vacuum drying oven at 65°C for 1 h before calculation of net lipid content. Transmethylation was performed by incubating the extracted lipids with 20 μL 2 mg/mL tridecanoate, 200 μL chloroform:methanol (2:1, v/v), and 300 μL 5% HCL:methanol (v/v) at 85°C for 1 h. The fatty acid methyl esters (FAMEs) were extracted with hexane and analyzed directly by GC–MS using the Agilent 7890A GC system with an Agilent 19091-N133 column. Mixed analytical standards of FAMEs and pentadecane were used as external and internal standards, respectively. The amounts of the FA profiles were calculated on the basis of results derived from GC–MS. The chemicals used as standards were from Sigma.

TAGs were separated on a silica thin layer chromatography plate using a mixture of solvents consisting of petroleum ether, ethyl ether, and acetic acid (70:30:1 by volume), and TAG bands were scraped from the plate. FAMEs were prepared by acid-catalyzed transmethylation of the TAG bands and then analyzed by GC–MS as described previously. TAG amounts were calculated on the basis of results derived from GC–MS.

## Data and code availability

For PacBio sequencing, raw sequencing data from the 19 runs have been deposited into the Sequence Read Archive (SRA) by the DOE Joint Genome Institute as SRR2022894 to SRR2022912. For Hi-C sequencing data, raw reads have been deposited into the SRA under accession SRR8420587. For MinION sequencing, raw signal files are available from the SRA under accession SRR8417804. The genome assembly, annotation, and methylation analysis results can be downloaded from the NanDeSyn database (https://nandesyn.single-cell.cn/download; Gong et al., 2020). RNA-seq and 6mA profiling data from the mutants have been deposited in the GEO database with accession numbers GSE178672 and GSE212585. Accession numbers for the sequence data used in this study are provided in Supplemental Table 9. Raw sequence data reported in this paper have also been deposited in the Genome Sequence Archive at the National Genomics Data Center, China National Center for Bioinformation, under accession number PRJCA021066.

## Funding

This work was supported by the Synthetic Biology Program of the 10.13039/501100002855Ministry of Science and Technology of the People's Republic of China (2021YFA0909700), I201908 and E1551402 from the Qingdao Institute of Bioenergy and Bioprocess Technology, 10.13039/501100002367Chinese Academy of Sciences, 31900071 from the 10.13039/501100001809Natural Science Foundation of China, and ZR2019QC012 from the 10.13039/501100007129Natural Science Foundation of Shandong Province. We thank Professor Byeong-ryool Jeong for discussions.

## Author contributions

J.X., Y.G., and L.W. conceived and designed the study; Q.W. and L.W. performed the experiments; Y.G. analyzed the sequencing data; W.L., L.W., N.L., X.D., J.Z., C.S., and Y.X. contributed to experiments and data interpretation; L.S. advised on manuscript writing; Y.G. and J.X. wrote the paper.

## Supplemental information

Document S1. Supplemental Results, Supplemental Tables 1–9, and Supplemental Figures 1–9

File S1. List of DEGs for the wild type and knockout mutants of *N. oceanica* based on RNA-seq data

Document S2. Article plus supplemental information
