# Supplementary material for: Genome-wide adenine N6-methylation map reveals epigenomic regulation of lipid accumulation in Nannochloropsis
Source: Plant Commun. 2023 Nov 24;5(3):100773. doi: 10.1016/j.xplc.2023.100773 (PMC10943562; doi:10.1016/j.xplc.2023.100773)
Supplement: Document S1. Supplemental Results, Supplemental Tables 1–9, and Supplemental Figures 1–9 [file mmc1.pdf]

Plant Communications, Volume *5*

Supplemental information

Genome-wide adenine N6-methylation map reveals epigenomic regulation of lipid accumulation in *Nannochloropsis*

Yanhai Gong, Qintao Wang, Li Wei, Wensi Liang, Lianhong Wang, Nana Lv, Xuefeng Du, Jiashun Zhang, Chen Shen, Yi Xin, Luyang Sun, and Jian Xu

Supplemental Results

Nannochloropsis oceanica genome assembly using the PacBio and Hi-C data

The most high quality nuclear genome of *N. oceanica* IMET1 published up to date (Wang et al., 2014) is fragmented (293 scaffolds) and with significant amount of ambiguous bases (11%) which will lead to low accuracy of 6mA detection. The lack of a high-quality reference genome impedes investigations of genome-wide 6mA distribution patterns and other downstream studies. Therefore, we started by *de novo* assembly for an improved nuclear genome using ultra-high depth of PacBio and Hi-C data. We generated ~340X sequencing coverage of the *N. oceanica* IMET1 genome using 19 SMRT cells on the PacBio RS-II platform (**Methods**; **Table S1**). The resulting subreads had a read N50 length of over 9.43 Kb (**Table S1**). The longest ~120X subreads were self-corrected using all the subreads, then the longest ~50X error-corrected reads were assembled using Celera assembler (PBcR; (Berlin et al., 2015)) followed by polishing via Quiver (**Supplemental Methods**). The assembly contained 122 contigs spanning 31.54 Mb genome size with N50 length 907.6 Kb and without any gaps (ambiguous bases). Then, a repeat-aware de novo assembly upgrading tool -- FinisherSC (Lam et al., 2015) was used to resolve repeated genome structures and improve contiguity followed by another round of polishing via Quiver (**Supplemental Methods**). Finally, putative contaminations were removed. Above assembly strategy helped us to obtain 70 contigs with total length of 31.0 Mb (with N50 length of 1.08 Mb; **Table S2**). We also found 11 contigs with telomeric repeats (TTAGGG; (Corteggiani Carpinelli et al., 2014)) at both extremities and 33 contigs with only one telomere.

To further improve the continuity of nuclear genome and thoroughly resolve its chromosome architectures, we utilized a high-resolution contact map derived from ultra-high

depth of Hi-C data (**Supplemental Methods**). Hi-C interaction maps share common features which relate 3D interaction frequencies to the 1D ordering of the genome (Oddes et al., 2018), and have been used to improve draft genome assemblies to create chromosome-length scaffolds for large genomes (Bickhart et al., 2017; Dudchenko et al., 2017; Mascher et al., 2017). We obtained ultra-high (~2000X) coverage of *in situ* Hi-C data, and 319.6 million read pairs (92.16%) passed the quality filter to constitute the clean data. Data analysis showed 73.8% pairs were uniquely mapped and 72.2% were valid interaction pairs (55.6% after removing PCR duplicates). Initial scaffolding attempts using LACHESIS (Burton et al., 2013) and 3d-dna (Dudchenko et al., 2017) clustered all contigs into one chromosome. To maximize the number of ordered and oriented contigs, we manually scaffolded these contigs mainly based on the inter-contigs contact map (generated by LACHESIS). Two aberrant contigs were rejected with careful examination; then, the positions of telomeres were leveraged to resolve potential conflicts/ambiguities; in the end, the scaffoldings were rechecked by inspecting the contact map of scaffolded pseudo-chromosomes. Previous analyses of potential connections between contigs are consistent with the manual scaffolding based on Hi-C.

The final assembly (NoIMET1v2) were scaffolded into 30 pseudo-chromosomes (**Table S2**) which is consistent with the estimation for *N. gaditana* (Corteggiani Carpinelli et al., 2014; Schwartz et al., 2018) (**Fig. S1A**). The NoIMET1v2 assembly had 9.8-fold higher contiguity than NoIMET1v1, ~1,500 gaps eliminated/filled, and with average base accuracy at ~99.988% (**Fig. S1B** and **Table S2**). Besides, compared with data that were obtained using NoIMET1v1 (Wang et al., 2014) as reference, the 6mA map reveals ~12% more 6mA sites, while the average distance between adjacent 6mA sites is 9.2% lower (Wilcoxon test, $p < 0.001$; **Fig. S3**).

Genome-wide annotation of transposons and gene models

The improved genome of NoIMET1v2 enabled complete genome-wide annotation of transposons, which could potentially affect the function of DNA 6mA. For example, in rice, transposon associated genes (TEGs) with the highest expression displayed the lowest 6mA levels at the TSS, suggesting that 6mA at the TSS represses TEG expression (Zhou et al., 2018). In *Drosophila melanogaster*, 6mA sites are enriched in transposable elements and correlated with transposon expression (Zhang et al., 2015). So, we firstly identified repeat elements genome-wide through *de novo* repeat family identification, and annotated 16.2% of the assembly as repetitive element content (**Supplemental Methods**; **Fig. S1C**). Besides simple repeats (7.08%) and low complexity fragments (2.44%), DNA elements were the most abundant (3.97%), of which the DNA/CMC-EnSpm elements occupied 3.84% of the genome (**Fig. S1C**). En/Spm is the most thoroughly studied member of the CACTA TE superfamily having TIRs terminating with the sequence CACTA and the creation of 3-bp TSDs (Gbadegesin and Beeching, 2010). Apart from the main groups of DNA elements, 0.65% of the genome was annotated as long interspersed nuclear elements (LINE) and 0.12% as LTR elements, whereas the remainder was either assigned to other repeat families or could not be assigned (**Fig. S1B** and **Fig. S1C**). Among the several sequenced algae and plant species (**Table S3**), the repetitive elements identified in the *N. oceanica* genome were comparable to those in diatom (TEs contribute 6.4% of the *P. tricornutum* genome and 1.9% of the *T. pseudonana* genome, with LTR-RTs the most abundant in both genomes; (Maumus et al., 2009)). Class I elements (retrotransposons or retroelements) are the most widespread class of eukaryotic TEs (International Human Genome Sequencing et al., 2001; SanMiguel et al., 1996), but in *N. oceanica* DNA/CMC-EnSpm elements are the most

abundant TEs, which could serve as a valuable model to study the influence of DNA/CMC-EnSpm elements.

The initial set of *N. oceanica* IMET1 gene models for NoIMET1v1 genome was generated using EVidenceModeler (Haas et al., 2008) based on a limited amount of transcriptomic information (387K 454 cDNA reads; (Wang et al., 2014)) in nitrogen-repletion/starvation conditions (three time points; no replicates) and therefore involved a significant amount of *de novo* prediction. To provide diversity of transcripts, ~307 million pairs of reads, obtained from 54 RNA-Seq libraries (GEO datasets GSE42508 and GSE55861) among nitrogen-repletion/starvation conditions (six time points; three replicates) and CO_2-starvation conditions (five time points; two replicates) were used as evidence (**Supplemental Methods**). As a starting point, 10,353 protein-coding genes were predicted using BRAKER1 (Hoff et al., 2016) with hints from the alignments of these RNA-Seq data. These gene models were further improved by exploiting spliced alignments of transcript sequences using PASA2 (Haas et al., 2011) pipeline to annotate UTRs, add alternative splicing variants, merge/split genes, adjust boundaries and model novel genes (**Supplemental Methods**). Finally, 10,333 gene models (12,442 isoforms) were obtained, including 1,777 novel gene loci (**Table S4**). These protein-coding genes were further analyzed to predict biological functions by comparing protein sequences with the NR, InterPro and eggNOG databases (**Supplemental Methods**). This process allowed functional annotations to be assigned to 88.4%, 77.7% and 68.2% transcripts, respectively (**Table S4**).

Genome sequence validation using 454 and RNA-Seq data

Reads from 454 sequencing (Wang et al., 2014) were mapped to NoIMET1v1 and NoIMET1v2 respectively (**Supplemental Methods**). For NoIMET1v2, 1,834 high confidence

SNPs and 638 high confidence indels were found, which was less than half compared to NoIMET1v1 (**Fig. S1D**). RNA-Seq reads (from GSE42508 and GSE55861) were also individually mapped (**Supplemental Methods**) with 91.78% mapping ratio compared with 89.91% for NoIMET1v1. Similarly, the number of variants found was about 30% less (**Fig. S1D**).

Assessment of DNA contamination on 6mA detection based on simulated datasets

The abundance of DNA 6mA in eukaryotes may be overestimated due to prokaryotic DNA and RNA contamination in the original genomic DNA samples or in the enzymes for sample processing (Kong et al., 2022; O'Brown et al., 2019). To tackle this problem, firstly, during 6mA detection, we applied strict parameters for subread filtering and aligning (**Methods**), which greatly reduces false positives; then, based on multiple simulated datasets of PacBio sequencing data from NCBI BioProject PRJNA477598 (McIntyre et al., 2019), we assessed the degree to which the contaminated reads would interfere with the 6mA map of *N. oceanica*. We simulated the situation of DNA contamination by manually adding PacBio sequencing data from multiple microorganisms (yeast and seven bacteria) and generated nine mock datasets (**Supplemental Methods**). Detection of 6mA on these datasets (**Table S6**) revealed that: (*i*) very few such yeast and bacterial PacBio subreads (<1‰ in bases) would be aligned to the reference genome of *N. oceanica*; (*ii*) adding these contaminating DNA hardly changed the number and predicted loci of 6mA in the *N. oceanica* genome. Therefore, it is unlikely that the genome-wide 6mA map for *N. oceanica* is skewed by bacterial and fungal DNA contaminations.

Supplemental Methods

Hi-C library preparation and sequencing

Following the standard protocol described previously with certain modifications (Belton et al., 2012), we constructed Hi-C libraries using the *N. oceanica* cells as inputs (under low and high CO_2 conditions). Briefly, the microalgal cells were cross-linked by 2% formaldehyde solution at room temperature in a vacuum for 30 mins. 2.5 M glycine was added to quench the crosslinking reaction for 10 mins at room temperature. After ground with liquid nitrogen and re-suspended with 25 ml of extraction buffer I (0.4 M sucrose, 10 mM Tris-HCl, pH 8, 10 mM $MgCl_2$, 5 mM β-mercaptoethanol, 0.1 mM phenylmethylsulfonyl fluoride [PMSF], and 13 units protease inhibitor), the mixture supernatant was further centrifuged at 4000 rpm at 4 °C for 20 mins. Re-suspended pellet in extraction buffer II (0.25 M sucrose, 10 mM Tris-HCl, pH 8, 10 mM MgCl2, 1% Triton X-100, 5 mM β-mercaptoethanol, 0.1 mM PMSF, and 13 units protease inhibitor) was centrifuged at 14,000 rpm and 4 °C for 10 mins. The pellet was re-suspended in extraction buffer III (1.7 M sucrose, 10 mM Tris-HCl, pH 8, 0.15% Triton X-100, 2 mM MgCl2, 5mM β-mercaptoethanol, 0.1 mM PMSF, and 13 units protease inhibitor) and loaded on the top of an equal amount of clean extraction buffer III, which was then centrifuged at 14,000 rpm for 10 mins. The pellet was washed twice in 500 μL ice cold 1x CutSmart buffer and then centrifuged for 5 mins at 2,500 g. The nuclei were washed by 0.5 mL of restriction enzyme buffer and solubilized with dilute SDS followed by incubation at 65 °C for 10 min. After quenching the SDS by Triton X-100, an overnight digestion was applied to the samples with a 6-cutter restriction enzyme HindIII (400 units) at 37 °C on a rocking platform.

The subsequent steps involved marking the DNA ends with biotin-14-dCTP and blunt-end ligation of the cross-linked fragments. The proximal chromatin DNA was re-ligated by ligation enzyme. The nuclear complexes were revers cross-linked by incubation with the proteinase K at

65 °C. DNA was purified by the phenol-chloroform extraction. Biotin was removed from non-ligated fragment ends using T4 DNA polymerase. Ends of sheared fragments by sonication (200-600 base pairs) were repaired by the mixture of T4 DNA polymerase, T4 polynucleotide kinase and Klenow DNA polymerase. Biotin-labeled Hi-C samples were specifically enriched using streptavidin C1 magnetic beads. After adding A-tails to the fragment ends and following ligation by the illumina paired-end (PE) sequencing adapters, Hi-C sequencing libraries were amplified by PCR (10-15 cycles) and sequenced on Illumina HiSeq-2500 platform (PE 125bp).

Genome assembly based on the PacBio and Hi-C data for *N. oceanica*

PacBio reads were sequenced using 19 SMRT cells with P6-C4 chemistry on the PacBio RS-II platform (**Methods**). PacBio subreads were first *de novo* assembled using PBcR in Celera Assembler (Berlin et al., 2015)with parameters "-length 500 -partitions 200 -maxCoverage 120", then polished using Quiver tool from SMRT-Analysis package version 2.3.0. After that, FinisherSC (finishingTool-2.1; (Lam et al., 2015)) was used to resolve potential repeats and promote continuity. Finally, another round of Quiver was used to improve base qualities.

The PacBio only assembly was visualized by TAGC (Taxon-annotated Gc-Coverage) plot using Blobtools (Laetsch and Blaxter, 2017), for this, taxon annotations were generated from blasting to NCBI NT database using parameters "-culling_limit 5 -evalue 1e-25" and UniRef90 database using "diamond blastx" tool (Buchfink et al., 2015), coverages of every contig/scaffold were calculated from remapping PacBio subreads using pbalign (from SMRT-Analysis). Contigs/scaffolds without proper annotations (not alga) were manually examined and contaminated ones were removed from the assembly.

One high-resolution Hi-C library (high CO_2) was prepared and sequenced using an Illumina

HiSeq instrument with PE150 layout to yield approximately 2000X coverage of the *N. oceanica* IMET1 genome. Raw sequencing reads were filtered by removing adapter contaminated reads, low quality reads and reads with more than 5% ambiguous bases. Clean reads were mapped to the draft genome using Juicer v1.5.6 (Durand et al., 2016) and HiC-Pro v2.9.0 (Servant et al., 2015). LACHESIS (Burton et al., 2013) and 3d-dna (Dudchenko et al., 2017) were tested to scaffold PacBio assembled contigs, but both pipelines assembled all contigs into one chromosome which was unreasonable. To resolve potential conflicts, the inter-contigs contact map generated using LACHESIS (Burton et al., 2013) was used as global reference, then orders and orientations between contigs were manually examined with the assistance of Juicebox (Durand et al., 2016) for visualization of Hi-C contact matrix and revised with the assistance of external information such as position of telomeres in contigs, PacBio reads connection between contigs and synteny between other *Nannochloropsis* species. Synteny analysis between *N. oceanica* NoIMET1v1, *N. oceanica* NoIMET1v2, *N. oceanica* CCMP1779, *N. salina* CCMP1776, *N. gaditana* B-31 and *C. reinhardtii* v4.0 were performed using SyMAP ((Soderlund et al., 2011); with parameters to allow merging of synteny blocks).

Assessment of genome assembly NoIMET1v2

To evaluate the quality of NoIMET1v2, 454 clean reads (Wang et al., 2014) were mapped to the assembly using runMapping from 454 Sequencing System Off-Instrument Software Applications suite, SNP information were extracted from the output files. RNA-Seq reads from each library were mapped to the genome using HISAT2 ((Pertea et al., 2016); with parameters "--max-intronlen 2000 --dta -k 1 -X 1000 --no-mixed --no-discordant --mm"), variants were called using HaplotypeCaller from GATK ((Auwera et al., 2013); with parameters "-

dontUseSoftClippedBases -stand_call_conf 20.0 -stand_emit_conf 20.0") and filtered using VariantFiltration (with parameters: -window 35 -cluster 3 -filterName FS -filter "FS > 30.0" -filterName QD -filter "QD < 2.0").

Genome-wide annotation for NoIMET1v2

RNA-Seq data were used to assist the identification and structural annotation of genes. These data were previously sequenced and taken from GSE42508 (Li et al., 2014) and GSE55861 (Wei et al., 2019). All the raw reads were quality controlled using a script (from http://justpreprocessmyreads.sourceforge.net) with parameters "-cdna -qtrim 10 -slide_window 8 -slide_quality 20 -min_length 70". All the RNA-Seq clean reads were mapped to the genome using HISAT2 (Kim et al., 2015) (see above). A primitive reference annotation was made using BRAKER1 (Hoff et al., 2016) with evidence hints from all RNA-Seq mapping files. Then, the gene models were refined (annotating UTRs, creating alternatively spliced isoforms, etc) using PASA2 (Haas et al., 2003) served on the online platform GenSAS v5.1 (Lee et al., 2011) (https://www.gensas.org). During the refinement, the RNA-Seq data sets were in silico normalized to targeted maximum read coverage of 100 using Trinity (Grabherr et al., 2011).

The resultant protein isoforms were annotated via searching against the NCBI NR database using diamond blastp (diamond 0.8.36, evalue 1e-6, max_target_seqs 1) and predicting protein families and functional domains using InterProScan v5 (Jones et al., 2014) with parameters "--applications Pfam, PRINTS, ProDom, ProSiteProfiles, SMART, SUPERFAMILY, PANTHER -dp -iprlookup". Then, GO/KO/COG annotations were inferred based on orthology assignments from eggNOG database using eggNOG-Mapper (Huerta-Cepas et al., 2017).

Additionally, repeats were identified and masked using RepeatModeler and RepeatMasker

(http://www.repeatmasker.org), potential ncRNAs were inferred using Infernal cmscan (Nawrocki and Eddy, 2013) by searching the CM-format Rfam database with parameters "--rfam --nohmmonly --fmt 2 --cut_ga", tRNA and rRNA locus were extracted from the output and combined with the outputs from tRNAscan-SE ((Lowe and Eddy, 1997); Cove mode), barrnap (https://github.com/tseemann/barrnap; with parameters: "--kingdom euk") and RNAmmer ((Lagesen et al., 2007); with parameters: "-S euk -m lsu,ssu,tsu -multi") manually.

Detection of 6mA modifications for the datasets that simulated microbial contamination

To test the influence of DNA contamination on the calling of 6mA from our *N. oceanica* PacBio data, simulated datasets were used for comparison. These simulated datasets were synthesized by mixing *N. oceanica* PacBio reads with PacBio reads from different microbial sources (**Table S6**). Firstly, eight PacBio sequencing datasets (originated from *Bacillus subtilis*, *Enterococcus faecalis*, *Escherichia coli*, *Listeria monocytogenes*, *Pseudomonas aeruginosa*, *Saccharomyces cerevisiae*, *Salmonella enterica* and *Staphylococcus aureus*, respectively) were obtained from NCBI BioProject PRJNA477598 (McIntyre et al., 2019). Secondly, each dataset was combined with the PacBio sequencing data of *N. oceanica* to make eight simulated datasets, and a special simulated dataset ("Altogether", which represents the situation of heavy contamination) was generated by combining the eight collected datasets and *N. oceanica* data. Finally, the DNA 6mA sites for the nine simulated datasets were detected (**Methods**) and compared with the 6mA sites used in this study. Collectively, 99.8% of all 6mA sites were consistent between the two, even under the circumstance of heavy contamination (i.e., the simulated dataset of "Altogether").

References

Auwera, G.A., Carneiro, M.O., Hartl, C., Poplin, R., Angel, G.d., Levy‐Moonshine, A., Jordan, T., Shakir, K., Roazen, D., Thibault, J., *et al.* (2013). From FastQ data to high‐confidence variant calls: the genome analysis toolkit best practices pipeline. Current Protocols in Bioinformatics *43*, 11.10.11-11.10.33.

Belton, J.-M., McCord, R.P., Gibcus, J.H., Naumova, N., Zhan, Y., and Dekker, J. (2012). Hi-C: a comprehensive technique to capture the conformation of genomes. Methods *58*, 268-276.

Berlin, K., Koren, S., Chin, C.-S., Drake, J.P., Landolin, J.M., and Phillippy, A.M. (2015). Assembling large genomes with single-molecule sequencing and locality-sensitive hashing. Nature Biotechnology *33*, 623.

Bickhart, D.M., Rosen, B.D., Koren, S., Sayre, B.L., Hastie, A.R., Chan, S., Lee, J., Lam, E.T., Liachko, I., Sullivan, S.T., *et al.* (2017). Single-molecule sequencing and chromatin conformation capture enable de novo reference assembly of the domestic goat genome. Nature Genetics *49*, 643.

Buchfink, B., Xie, C., and Huson, D.H. (2015). Fast and sensitive protein alignment using DIAMOND. Nature Methods *12*, 59-60.

Burton, J.N., Adey, A., Patwardhan, R.P., Qiu, R., Kitzman, J.O., and Shendure, J. (2013). Chromosome-scale scaffolding of de novo genome assemblies based on chromatin interactions. Nature Biotechnology *31*, 1119.

Corteggiani Carpinelli, E., Telatin, A., Vitulo, N., Forcato, C., D'Angelo, M., Schiavon, R., Vezzi, A., Giacometti, G.M., Morosinotto, T., and Valle, G. (2014). Chromosome scale genome assembly and transcriptome profiling of *Nannochloropsis gaditana* in nitrogen depletion. Molecular Plant *7*, 323-335.

Dudchenko, O., Batra, S.S., Omer, A.D., Nyquist, S.K., Hoeger, M., Durand, N.C., Shamim, M.S., Machol, I., Lander, E.S., Aiden, A.P., *et al.* (2017). De novo assembly of the *Aedes aegypti* genome using Hi-C yields chromosome-length scaffolds. Science *356*, 92-95.

Durand, N.C., Shamim, M.S., Machol, I., Rao, S.S.P., Huntley, M.H., Lander, E.S., and Aiden, E.L. (2016). Juicer provides a one-click system for analyzing loop-resolution Hi-C experiments. Cell Systems *3*, 95-98.

Gbadegesin, M.A., and Beeching, J.R. (2010). Enhancer/Suppressor mutator (En/Spm)-like transposable elements of cassava (*Manihot esculenta*) are transcriptionally inactive. Genetics and Molecular Research *9*, 639-650.

Grabherr, M.G., Haas, B.J., Yassour, M., Levin, J.Z., Thompson, D.A., Amit, I., Adiconis, X., Fan, L., Raychowdhury, R., Zeng, Q., *et al.* (2011). Full-length transcriptome assembly from RNA-Seq data without a reference genome. Nature Biotechnology *29*, 644-652.

Haas, B.J., Delcher, A.L., Mount, S.M., Wortman, J.R., Smith, R.K., Jr., Hannick, L.I., Maiti, R., Ronning, C.M., Rusch, D.B., Town, C.D., *et al.* (2003). Improving the *Arabidopsis* genome annotation using maximal transcript alignment assemblies. Nucleic Acids Research *31*, 5654-5666.

Haas, B.J., Salzberg, S.L., Zhu, W., Pertea, M., Allen, J.E., Orvis, J., White, O., Buell, C.R., and Wortman, J.R. (2008). Automated eukaryotic gene structure annotation using EVidenceModeler and the Program to Assemble Spliced Alignments. Genome Biology *9*, R7.

Haas, B.J., Zeng, Q., Pearson, M.D., Cuomo, C.A., and Wortman, J.R. (2011). Approaches to fungal genome annotation. Mycology *2*, 118-141.

Hoff, K.J., Lange, S., Lomsadze, A., Borodovsky, M., and Stanke, M. (2016). BRAKER1: unsupervised RNA-Seq-based genome annotation with GeneMark-ET and AUGUSTUS. Bioinformatics *32*, 767-769.

Huerta-Cepas, J., Forslund, K., Coelho, L.P., Szklarczyk, D., Jensen, L.J., von Mering, C., and Bork, P. (2017). Fast genome-wide functional annotation through orthology assignment by eggNOG-Mapper. Molecular Biology and Evolution *34*, 2115-2122.

International Human Genome Sequencing, C., Lander, E.S., Linton, L.M., Birren, B., Nusbaum, C., Zody, M.C., Baldwin, J., Devon, K., Dewar, K., Doyle, M., *et al.* (2001). Initial sequencing and analysis of the human

genome. Nature *409*, 860.

Jones, P., Binns, D., Chang, H.-Y., Fraser, M., Li, W., McAnulla, C., McWilliam, H., Maslen, J., Mitchell, A., Nuka, G.*, et al.* (2014). InterProScan 5: genome-scale protein function classification. Bioinformatics *30*, 1236-1240.

Kim, D., Langmead, B., and Salzberg, S.L. (2015). HISAT: a fast spliced aligner with low memory requirements. Nature Methods *12*, 357.

Kong, Y., Cao, L., Deikus, G., Fan, Y., Mead, E.A., Lai, W., Zhang, Y., Yong, R., Sebra, R., Wang, H.*, et al.* (2022). Critical assessment of DNA adenine methylation in eukaryotes using quantitative deconvolution. Science *375*, 515-522.

Laetsch, D., and Blaxter, M. (2017). BlobTools: Interrogation of genome assemblies [version 1; referees: 1 approved with reservations]. F1000Research *6*, 1287.

Lagesen, K., Hallin, P., Rodland, E.A., Staerfeldt, H.H., Rognes, T., and Ussery, D.W. (2007). RNAmmer: consistent and rapid annotation of ribosomal RNA genes. Nucleic Acids Research *35*, 3100–3108.

Lam, K.-K., LaButti, K., Khalak, A., and Tse, D. (2015). FinisherSC: a repeat-aware tool for upgrading de novo assembly using long reads. Bioinformatics *31*, 3207-3209.

Lee, T., Peace, C., Jung, S., Zheng, P., Main, D., and Cho, I. (2011). GenSAS — An online integrated genome sequence annotation pipeline. In 2011 4th International Conference on Biomedical Engineering and Informatics (BMEI), pp. 1967-1973.

Li, J., Han, D., Wang, D., Ning, K., Jia, J., Wei, L., Jing, X., Huang, S., Chen, J., Li, Y.*, et al.* (2014). Choreography of transcriptomes and lipidomes of *Nannochloropsis* reveals the mechanisms of oil synthesis in microalgae. The Plant Cell *26*, 1645-1665.

Lowe, T.M., and Eddy, S.R. (1997). tRNAscan-SE: a program for improved detection of transfer RNA genes in genomic sequence. Nucleic Acids Research *25*, 955-964.

Mascher, M., Gundlach, H., Himmelbach, A., Beier, S., Twardziok, S.O., Wicker, T., Radchuk, V., Dockter, C., Hedley, P.E., Russell, J.*, et al.* (2017). A chromosome conformation capture ordered sequence of the barley genome. Nature *544*, 427.

Maumus, F., Allen, A.E., Mhiri, C., Hu, H., Jabbari, K., Vardi, A., Grandbastien, M.-A., and Bowler, C. (2009). Potential impact of stress activated retrotransposons on genome evolution in a marine diatom. BMC Genomics *10*, 624.

McIntyre, A.B.R., Alexander, N., Grigorev, K., Bezdan, D., Sichtig, H., Chiu, C.Y., and Mason, C.E. (2019). Single-molecule sequencing detection of N6-methyladenine in microbial reference materials. Nature Communications *10*, 579.

Nawrocki, E.P., and Eddy, S.R. (2013). Infernal 1.1: 100-fold faster RNA homology searches. Bioinformatics *29*, 2933-2935.

O'Brown, Z.K., Boulias, K., Wang, J., Wang, S.Y., O'Brown, N.M., Hao, Z., Shibuya, H., Fady, P.-E., Shi, Y., He, C.*, et al.* (2019). Sources of artifact in measurements of 6mA and 4mC abundance in eukaryotic genomic DNA. BMC Genomics *20*, 445.

Oddes, S., Zelig, A., and Kaplan, N. (2018). Three invariant Hi-C interaction patterns: Applications to genome assembly. Methods *142*, 89-99.

Pertea, M., Kim, D., Pertea, G.M., Leek, J.T., and Salzberg, S.L. (2016). Transcript-level expression analysis of RNA-seq experiments with HISAT, StringTie and Ballgown. Nature Protocols *11*, 1650-1667.

SanMiguel, P., Tikhonov, A., Jin, Y.-K., Motchoulskaia, N., Zakharov, D., Melake-Berhan, A., Springer, P.S., Edwards, K.J., Lee, M., Avramova, Z.*, et al.* (1996). Nested retrotransposons in the intergenic regions of the maize genome. Science *274*, 765-768.

Schwartz, A.S., Brown, R., Ajjawi, I., McCarren, J., Atilla, S., Bauman, N., and Richardson, T.H. (2018). Complete

genome sequence of the model oleaginous alga *Nannochloropsis gaditana* CCMP1894. Genome Announcements *6*, e01448-01417.
Servant, N., Varoquaux, N., Lajoie, B.R., Viara, E., Chen, C.-J., Vert, J.-P., Heard, E., Dekker, J., and Barillot, E. (2015). HiC-Pro: an optimized and flexible pipeline for Hi-C data processing. Genome Biology *16*, 259.
Soderlund, C., Bomhoff, M., and Nelson, W.M. (2011). SyMAP v3.4: a turnkey synteny system with application to plant genomes. Nucleic Acids Research *39*, e68-e68.
Wang, D., Ning, K., Li, J., Hu, J., Han, D., Wang, H., Zeng, X., Jing, X., Zhou, Q., Su, X., *et al.* (2014). *Nannochloropsis* genomes reveal evolution of microalgal oleaginous traits. PLOS Genetics *10*, e1004094.
Wei, L., El Hajjami, M., Shen, C., You, W., Lu, Y., Li, J., Jing, X., Hu, Q., Zhou, W., Poetsch, A., *et al.* (2019). Transcriptomic and proteomic responses to very low CO_2 suggest multiple carbon concentrating mechanisms in *Nannochloropsis oceanica*. Biotechnology for Biofuels *12*, 168.
Zhang, G., Huang, H., Liu, D., Cheng, Y., Liu, X., Zhang, W., Yin, R., Zhang, D., Zhang, P., and Liu, J. (2015). N6-methyladenine DNA modification in *Drosophila*. Cell *161*, 893-906.
Zhou, C., Wang, C., Liu, H., Zhou, Q., Liu, Q., Guo, Y., Peng, T., Song, J., Zhang, J., Chen, L., *et al.* (2018). Identification and analysis of adenine N6-methylation sites in the rice genome. Nature Plants *4*, 554-563.

Supplemental Tables

Table S1. Statistics of reads from the PacBio RS-II sequencing. Whole genomic DNA of *N. oceanica* IMET1 was sequenced by 19 SMRT cells from PacBio RS-II system and P6-C4 chemistry on a size selected 20kb library. After base calling, 1.13 million polymerase reads, which account for ~340X sequencing depth, were obtained. After adapter trimming and quality filtering, 1.49 million PacBio subreads remained for genome assembly and base modification analysis with subread N50 length of 9.43 kb.

Metrics	Pre-filter	Post-filter	PacBio subreads
Number of bases	11.667 Gb	10.786 Gb	10.766 Gb
Number of reads	2,855,548	1,127,196	1,489,782
Read N50	12,628	13,070	7,226
Mean read length	4,085	9,568	9,431
Mean read quality	0.355	0.815	-

Table S2. Genome assembly metrics of *N. oceanica*. The chromosome resolved assembly NoIMET1v2 had 9.8-fold higher contiguity than NoIMET1v1 and used as reference for calling DNA 6mA modifications.

Metrics	NoIMET1v1	IMET1pb	NoIMET1v2
# contigs	294	70	30*
Total bases	31.5 Mb	31.0 Mb	31.0 Mb
Avr. length	107.2 Kb	443 Kb	1.034 Mb
N25 length	1.171 Mb	1.332 Mb	1.471 Mb
N50 length	935.2 Kb	1.080 Mb	1.168 Mb
N90 length	150.5 Kb	248.9 Kb	685.9 Kb
GC %	47.79%	54.33%	54.33%
Ns	11.05%	0.00%	0.01%

Table S3. Comparison of TEs among multiple species. For algae and plants, super families from Class II TEs (DNA transposons) rarely become the most abundant TE type.

Species	TEs	Top TE super family	CMC-EnSpm
A. thaliana	19.5%	RC/Helitron (6.3%)	0.98%
C. reinhardtii	12.5%	LINEs (3.84%)	-
V. carteri	20.4%	LTR (3.68%)	-
P. tricornutum	6.4%	Ty1/Copia (~5.7%)	-
T. pseudonana	1.9%	Ty1/Copia (~0.9%)	-
O. sativa	19.9%	Ty3/gypsy (7.3%)	-
Z. mays	64.0%	LTR/RLG (34.88%)	-
S. cerevisiae	3.4%	LTR/Ty1-Ty5	-
human	44.0%	ALU	-
zebrafish	51.2%	DNA (19.31%)	2.19%
N. oceanica	16.2%	DNA/CMC-EnSpm	3.84%

Table S4. Statistics for genome annotation of *N. oceanica*. NoIMET1v2 was selected as reference sequence for the annotation. The annotation showed that 90.5% bases are covered by genes and 2,442 genes are overlapped.

Items		Number/Percent
**Structural prediction**		
Number of genes		10,333
Number of mRNAs		12,442
Number of exons		51,592
Number of introns		39,150
Number of CDS		12,442
Overlapping genes		2,442
Contained genes		628
Mean gene length		2,715
Mean mRNA length		2,890
Mean exon length		554
Mean intron length		190
Mean CDS length		1,639
Covered by genes		90.5%
Covered by CDS		65.8%
Mean mRNAs per gene		1
Mean exons per mRNA		4
Mean introns per mRNA		3
**Functional annotation**		
	NR	10,994 (88.4%)
	InterProScan	9,673 (77.7%)
EggNOG	GO	5,087 (40.9%)
EggNOG	KO	5,728 (46.0%)
EggNOG	COG	7,972 (64.1%)

Table S5. List of DNA 6mA associated genes in *N. oceanica*. In human, N6AMT1 was functionally validated as DNA 6mA methyltransferase. The putative DNA 6mA methyltransferases were selected based on homologous analysis.

Type	Organism	Gene id	Protein length
DNA 6mA methyltransferase	*Arabidopsis*	AT3G13440.1	278
	Rice	XP_015631415.1	285
	Chlamydomonas	Cre01.g036750.t1.2	223
	P. sojae	XP_009527935.1	209
	Human	N6AMT1	217
	Mouse	NP_080642.1	214
	Nannochloropsis	NO08G00280.1	234
	L. transversale	XP_021883074.1	215
	Drosophila	NP_001027221.1	224
	Tetrahymena	XP_001015373.1	257
DNA 6mA demethylase	*Nannochloropsis*	NO06G02500.1	506
	Chlamydomonas	Cre06.g278198.t1.1	558
	Arabidopsis	AT1G11780.1	344
	Rice	LOC_Os03g60190	370
	Human	ALKBH1	388

Table S6. Statistics of 6mA detection in the mock dataset for accessing potential bacterial and fungal contamination. The penultimate column records the number of 6mA sites detected from the mock datasets which augmented PacBio sequencing data of *N. oceanica* in this study. The last row represents a mock dataset which combines all the PacBio sequencing data from the eight microorganisms on the list. 6mA sites were detected based on the reference genome of *N. oceanica* IMET1 in this study. Based on only *N. oceanica* data, 24,450 6mA sites were detected.

**Species**	**SRA accession**	**Subread bases (G)**	**Mapped bases**	**# 6mA**	**# 6mA***	**Shared 6mA**
Bacillus subtilis	SRR7498042	0.94	0.08‰	4	24,450	100.0%
Enterococcus faecalis	SRR7415622	1.13	0.07‰	2	24,447	100.0%
Escherichia coli	SRR8154667<br>SRR8154668<br>SRR8154669<br>SRR8154675<br>SRR7498041<br>SRR7498044	4.43	0.01‰	4	24,449	100.0%
Listeria monocytogenes	SRR7415624	0.71	0.05‰	0	24,449	100.0%
Pseudomonas aeruginosa	SRR7498043	0.70	0.01‰	0	24,452	100.0%
Saccharomyces cerevisiae	SRR7498045<br>SRR7498046<br>SRR7498048	1.07	0.72‰	41	24,463	99.9%
Salmonella enterica	SRR7415626	0.56	0.01‰	0	24,453	100.0%
Staphylococcus aureus	SRR7415627	0.60	0.10‰	1	24,452	100.0%
Altogether	-	-	-	-	24,466	99.8%

366 **Table S7. Characteristics of DNA 6mA between the *N. oceanica* and *C. reinhardtii* genomes.**

367

	N. oceanica **(stramenopiles)**	***C. reinhardtii*** **(green algae)**
**Global 6mA level (6mA/A)**	~0.1%	~0.4%
**6mA preference (motif)**	ApT	AGGYV, GAGWG, GAVGT, etc.
**Enrichment in specific gene structure**	Splicing donor site (1-2 bp upstream) and 3'UTR	TSS
**Function in gene transcription**	Positive correlation with gene transcription	Marking active TSS

368

369

Table S8. Oligonucleotides used in the *in vitro* methylation and demethylation assays.

**6mA-oligo**	5'-CATGATACCTTATGGAA*(6mA)AGCATGCTTGTATTTCTTATGAACCATGA TACCTTATGGAAAGCATGCTTGTATTTCTTATGAAC-3'
**NC-oligo**	5'-CATGATACCTTATGGAAAGCATGCTTGTATTTCTTATGAACCATGA TACCTTATGGAAAGCATGCTTGTATTTCTTATGAAC-3'

Table S9. List of accession numbers for the sequence data used in this article. All the sequencing data were shared using public databases according to the listed accession numbers.

No.	Accession numbers	Description
1	SRR2022894-SRR2022912	19 PacBio sequencing runs
2	SRR8420587	Hi-C sequencing data
4	SRP017310	N+/N- mRNA-seq datasets
5	GSE55861	C+/C- mRNA-seq datasets
6	GSE178672	RNA-seq datasets for wild-type and mutants under high light
7	GSE212585	PacBio Sequel II sequencing data for the wild-type and mutants under high light
8	https://nandesyn.single-cell.cn/download	All the assemblies and other omics resources

Supplemental Figures

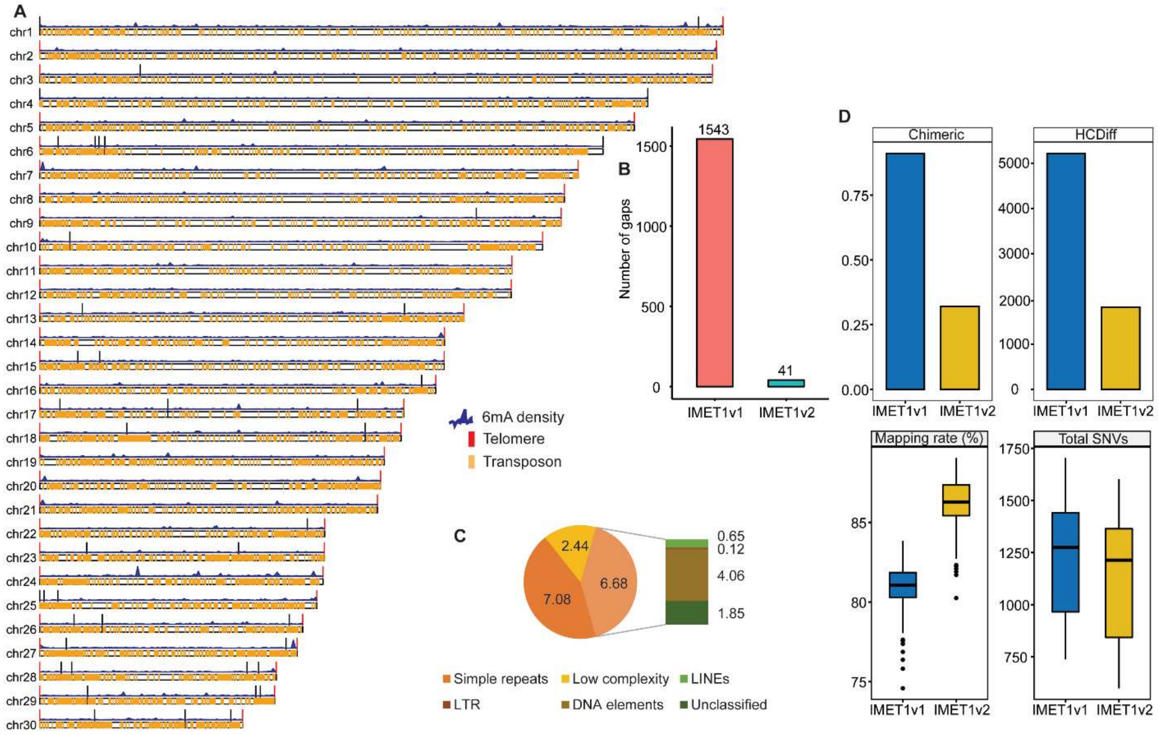

Figure S1. The improved genome sequence of *N. oceanica*. (**A**) The thirty chromosomes of NoIMET1v2. Notable features are shown above each bar with telomeres as red stripes, gaps as black stripes, and 6mA densities as blue curves (in 5 kb resolution). Transposons are shown inside each bar as orange regions. (**B**) Comparison between NoIMET1v1 and NoIMET1v2 in the number of gaps. (**C**) Repeat statistics for NoIMET1v2. DNA elements exceed retrotransposons (e.g., LTR and LINES) to become the most abundant transposons. (**D**) Evaluation of NoIMET1v2 using 454 and RNA-Seq data. Genomic (top) and transcriptomic (bottom) analysis reveal that the new assembly NoIMET1v2 is more complete than NoIMET1v1 and possesses bases with higher quality.

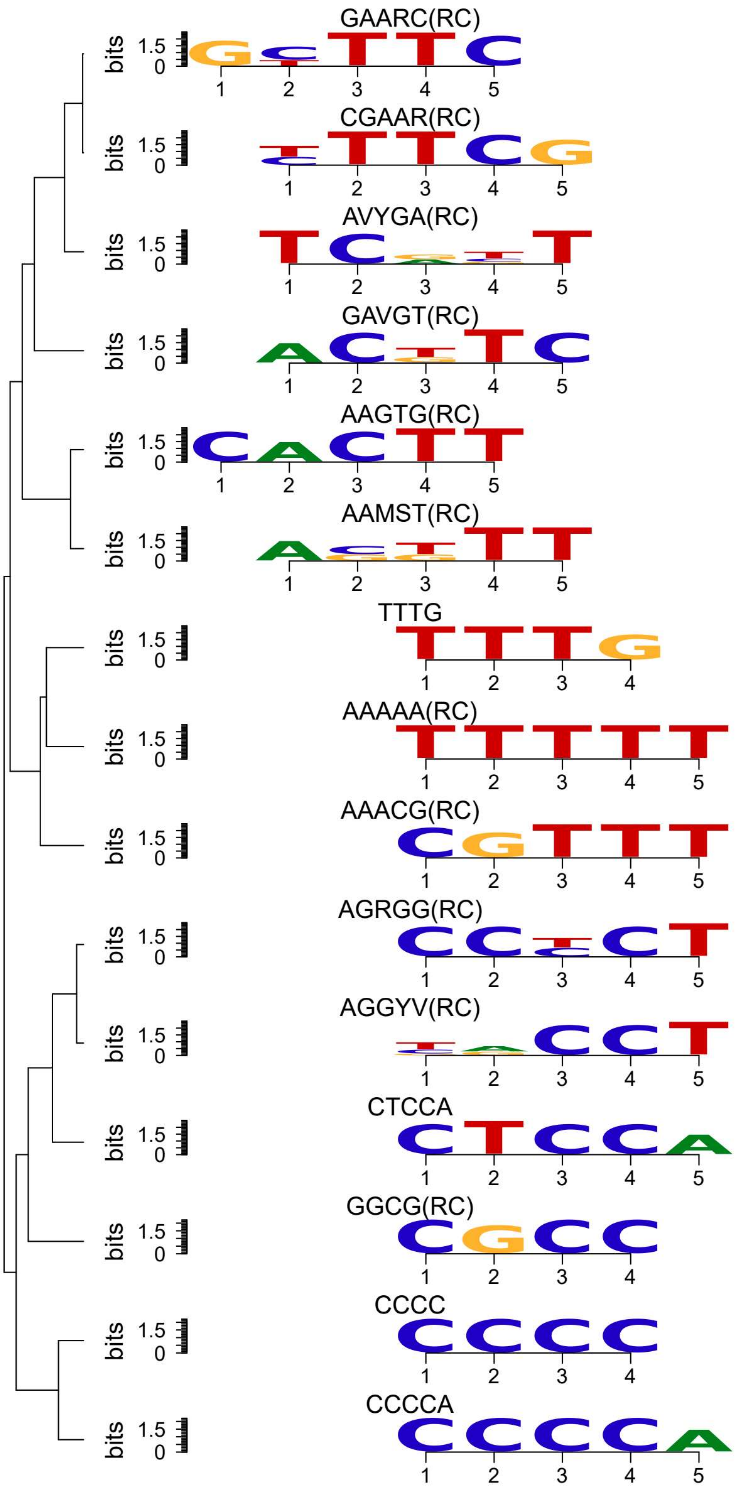

Figure S2. DNA 6mA enriched motifs in the *N. oceanica* genome. Totally 15 motifs were enriched in 6mA modified DNA fragments.

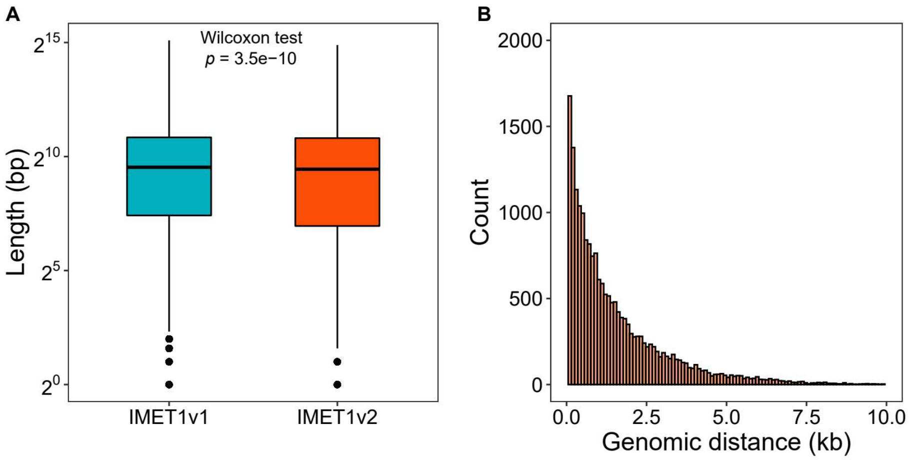

Figure S3. Distribution of 6mA events in the *N. oceanica* genome. (**A**) Distances of nearby 6mA sites detected using NoIMET1v1 and NoIMET1v2 as references. (**B**) Distribution of 6mA interval distances. Over 78% 6mA sites were with distance to nearest 6mA site within 2 kb which will affect the enrichment of 6mA-antibody.

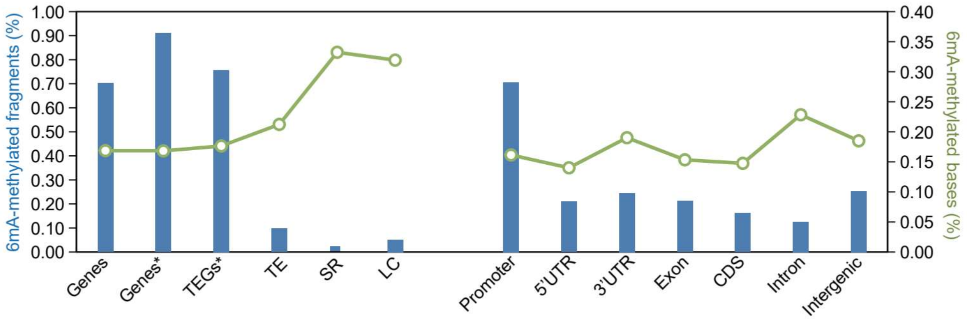

Figure S4. Global features of the 6mA events in the *N. oceanica* genome. The proportions and base proportions of 6mA events for different genomic regions (both strands). Genes: gene body; Genes*: gene body and 2kb promoter regions; TEGs*: Genes* overlapped with TEs; SR: simple repeats. LC: low complexity fragments; UTR: untranslated region.

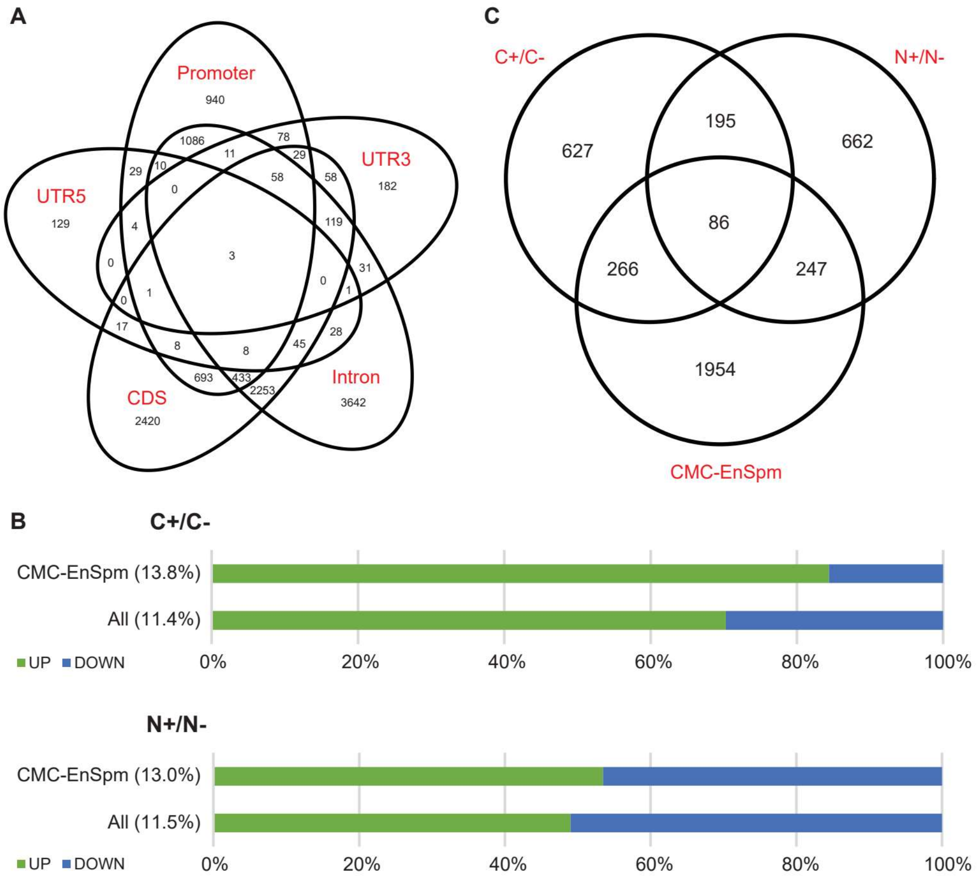

Figure S5. Transposable elements repress gene transcription in the *N. oceanica* genome. (**A**) Position statistics of TEs. (**B**) Many EnSpm-TEGs were differentially expressed with response to nitrate or CO_2 limitation. (**C**) Venn diagrams show relationships between nitrogen-depletion induced DEGs, CO_2-depletion induced DEGs and EnSpm-TEGs.

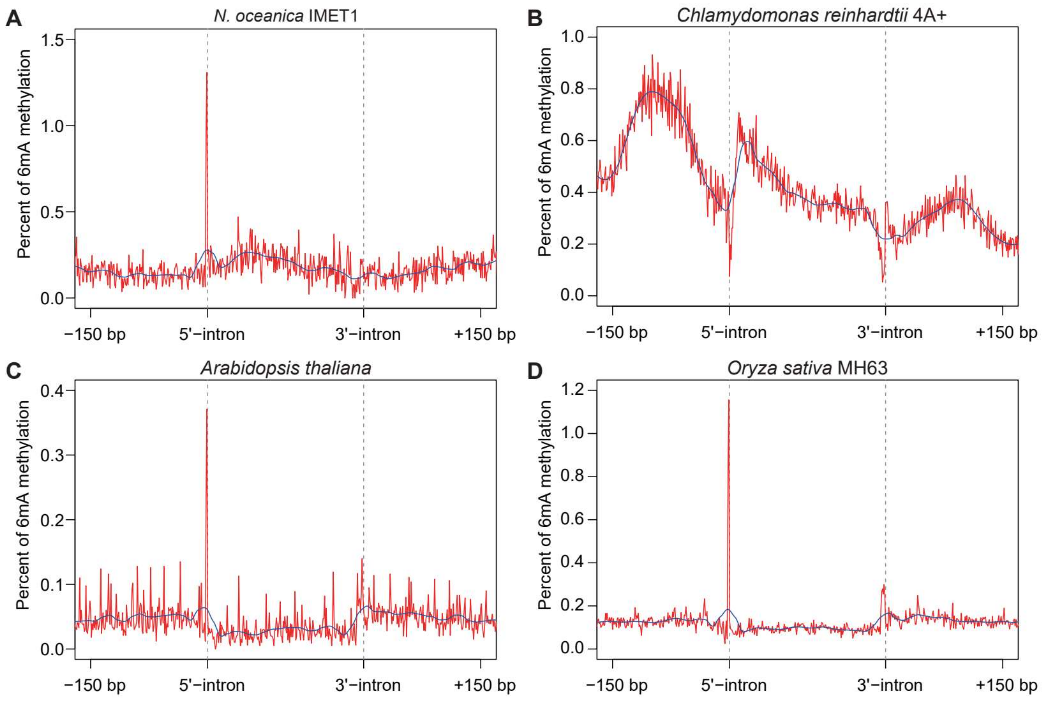

Figure S6. 6mA occupancy around intron (strand-specific) for *N. oceanica* (A), *Chlamydomonas reinhardtii* (B), *Arabidopsis thaliana* (C), and *Oryza sativa* (D). The strand of intron was defined as the strand of the same gene, each intron was consolidated into 200 bp. The 6mA occupancy data around intron was plotted with and without smoothing (blue and red).

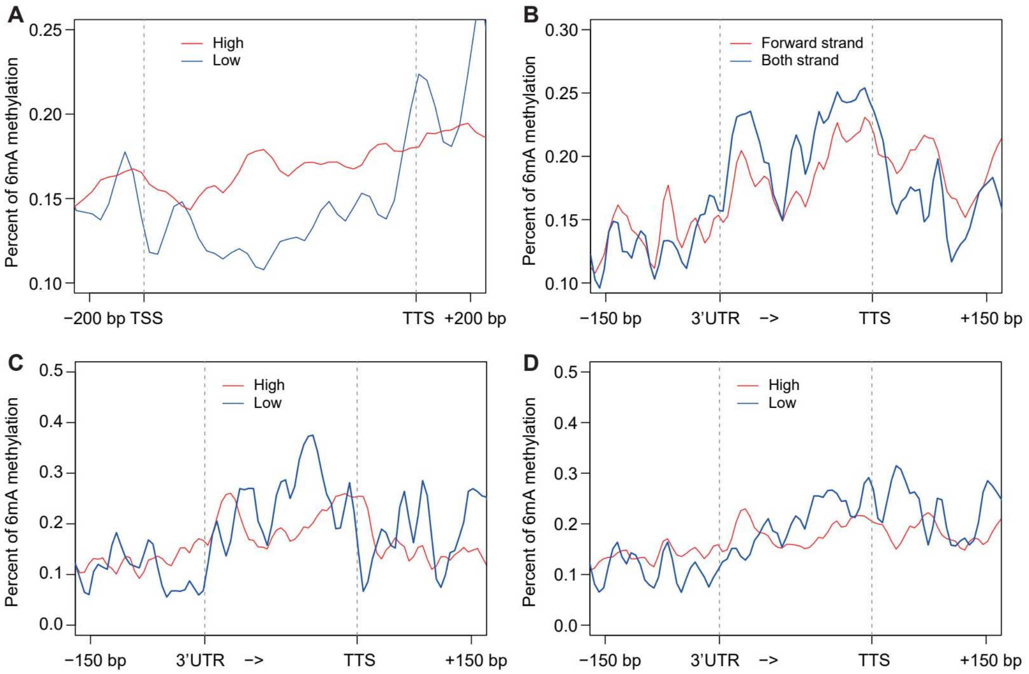

Figure S7. DNA 6mA occupancy along the genes and the TSSs in the *N. oceanica* genome. (**A**) 6mA occupancy along genes (non-strand-specific) for highly expressed genes (High: TPM>100) and poorly expressed genes (Low: TPM≤100). (**B**) 6mA occupancy along 3'UTRs (strand-specific: red line; non-strand-specific: blue line). (**C**) 6mA occupancy along 3'UTRs (strand-specific) for highly expressed genes (High: TPM>100) and poorly expressed genes (Low: TPM≤100). (**D**) 6mA occupancy along 3'UTRs (non-strand-specific) for highly expressed genes (High: TPM>100) and poorly expressed genes (Low: TPM≤100).

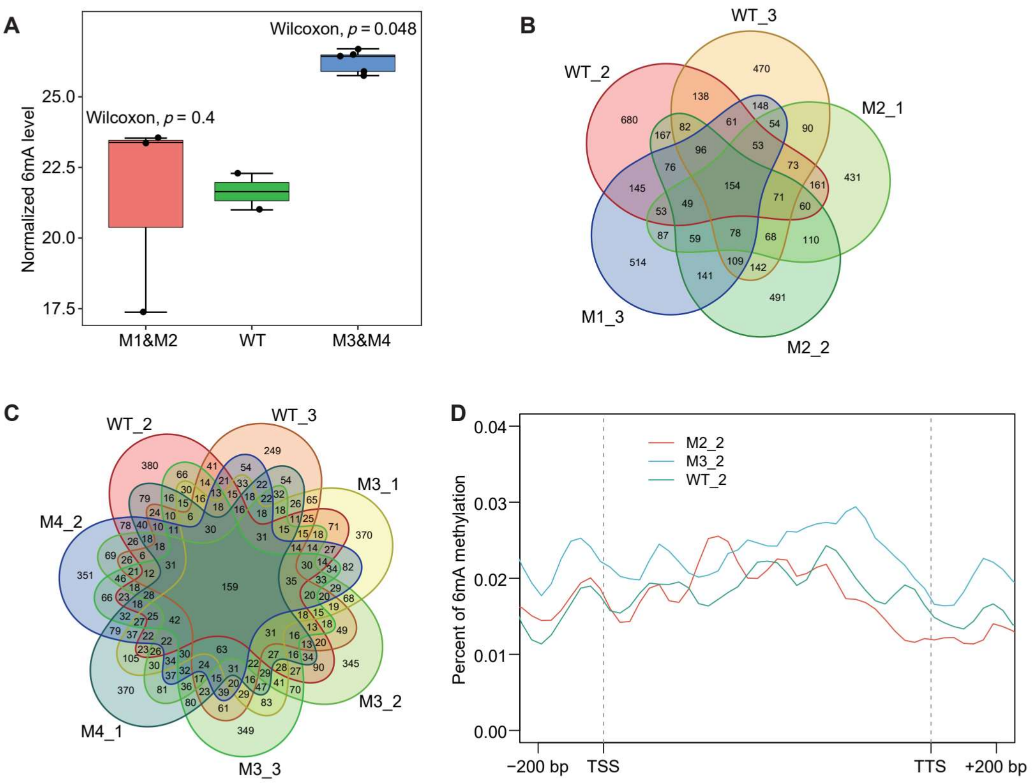

Figure S8. Characteristics of 6mA distribution in the wild-type versus the mutants of *N. oceanica*. (**A**) Comparison of 6mA levels in wild-type versus mutants. M1&M2 group includes Mutant 1 and 2; M3&M4 group contains Mutant 3 and 4. PacBio sequencing data for all the samples are subsampled to ~100X mappable subreads, then the 6mA levels are normalized (divide by) according to the final average base coverage. One-tailed Wilcoxon test is used to compare wild-type and mutant groups. (**B**) Overlap of 6mA-marked genes in M1, M2 and WT. (**C**) Overlap of 6mA-marked genes in M3, M4 and WT. (**D**) 6mA/A along protein-coding genes for M2, M3 and WT. One replicate for each group is shown.

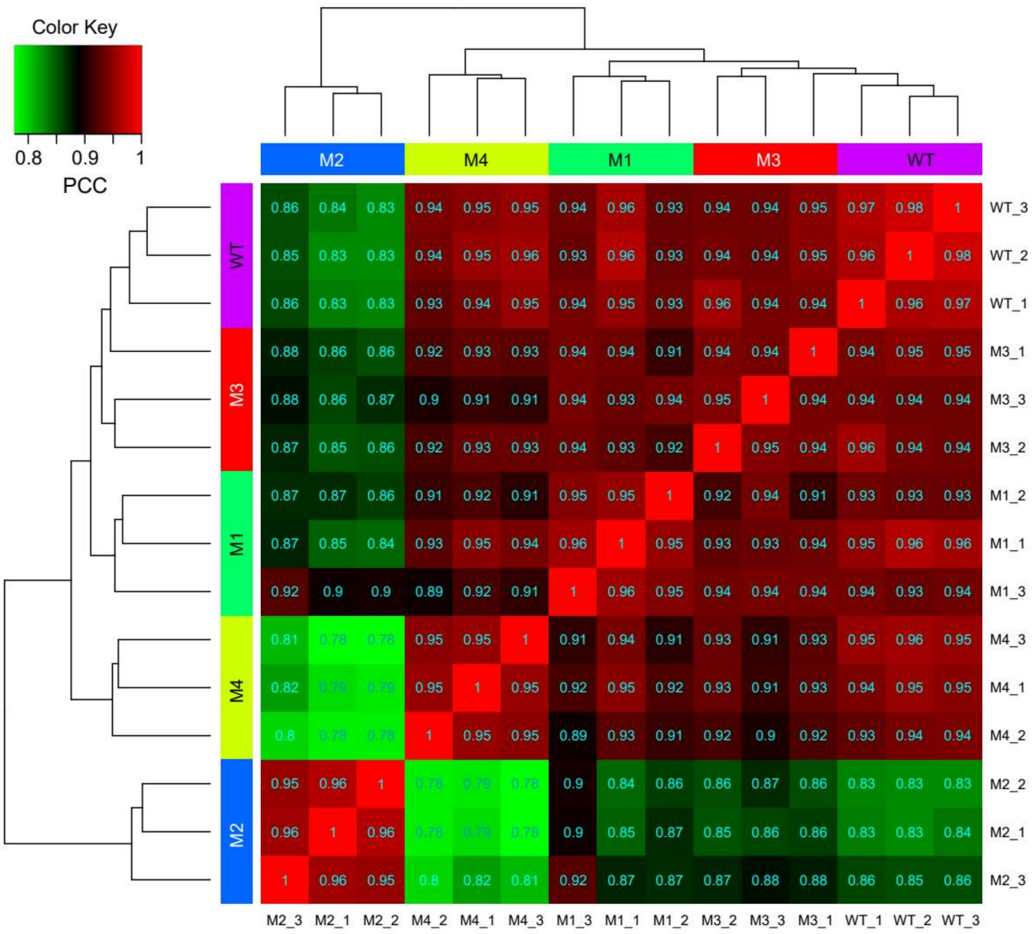

Figure S9. Sample correlation matrix for the wild-type and the knockout mutants of *N. oceanica* based on RNA-seq data. PCC: Pearson correlation coefficient. Algal cells were cultivated under high light and collected at 7 days. Mutants M1 and M2 are NO08G00280-knockout mutants; M3 and M4 are NO06G02500-knockout mutants. Expression values of differentially expressed genes were used to calculate the similarity between samples. All PCCs between replicates are > 0.94, which revealed excellent reproducibility among the biological replicates.
