# Supplementary material for: Genome-wide adenine N6-methylation map reveals epigenomic regulation of lipid accumulation in Nannochloropsis
Source: Plant Commun. 2023 Nov 24;5(3):100773. doi: 10.1016/j.xplc.2023.100773 (PMC10943562; doi:10.1016/j.xplc.2023.100773)
Supplement: Document S2. Article plus supplemental information [file mmc3.pdf]

Plant Communications
Research article

Genome-wide adenine N6-methylation map reveals epigenomic regulation of lipid accumulation in *Nannochloropsis*

Yanhai Gong[1,2,3,4,5,6], Qintao Wang[1,2,3,4,5,6], Li Wei[1,2,3,4,5], Wensi Liang[1,2,3,4,5], Lianhong Wang[1,2,3,4,5], Nana Lv[1,2,3,4,5], Xuefeng Du[1,2,3,4,5], Jiashun Zhang[1,2,3,4,5], Chen Shen[1,2,3,4,5], Yi Xin[1,2,3,4,5], Luyang Sun[1,2,3,4,5] and Jian Xu[1,2,3,4,5,*]

[1]Single-Cell Center, CAS Key Laboratory of Biofuels, Shandong Key Laboratory of Energy Genetics, Qingdao Institute of Bioenergy and Bioprocess Technology, Chinese Academy of Sciences, Qingdao, China

[2]Shandong Energy Institute, Qingdao, China

[3]Qingdao New Energy Shandong Laboratory, Qingdao, China

[4]University of Chinese Academy of Sciences, Beijing 100049, China

[5]Laboratory for Marine Biology and Biotechnology, Qingdao National Laboratory for Marine Science and Technology, Qingdao 266237, China

[6]These authors contributed equally to this article.

***Correspondence: Jian Xu (xujian@qibebt.ac.cn)**

https://doi.org/10.1016/j.xplc.2023.100773

ABSTRACT

Epigenetic marks on histones and DNA, such as DNA methylation at N6-adenine (6mA), play crucial roles in gene expression and genome maintenance, but their deposition and function in microalgae remain largely uncharacterized. Here, we report a genome-wide 6mA map for the model industrial oleaginous microalga *Nannochloropsis oceanica* produced by single-molecule real-time sequencing. Found in 0.1% of adenines, 6mA sites are mostly enriched at the AGGYV motif, more abundant in transposons and 3′ untranslated regions, and associated with active transcription. Moreover, 6mA gradually increases in abundance along the direction of gene transcription and shows special positional enrichment near splicing donor and transcription termination sites. Highly expressed genes tend to show greater 6mA abundance in the gene body than do poorly expressed genes, indicating a positive interaction between 6mA and general transcription factors. Furthermore, knockout of the putative 6mA methylase NO08G00280 by genome editing leads to changes in methylation patterns that are correlated with changes in the expression of molybdenum cofactor, sulfate transporter, glycosyl transferase, and lipase genes that underlie reductions in biomass and oil productivity. By contrast, knockout of the candidate demethylase NO06G02500 results in increased 6mA levels and reduced growth. Unraveling the epigenomic players and their roles in biomass productivity and lipid metabolism lays a foundation for epigenetic engineering of industrial microalgae.

Key words: adenine N6-methylation, industrial oleaginous microalgae, *Nannochloropsis oceanica*, transcriptional regulation, epigenomics

Gong Y., Wang Q., Wei L., Liang W., Wang L., Lv N., Du X., Zhang J., Shen C., Xin Y., Sun L., and Xu J. (2024). Genome-wide adenine N6-methylation map reveals epigenomic regulation of lipid accumulation in *Nannochloropsis*. Plant Comm. **5**, 100773.

INTRODUCTION

DNA N6-methyladenine (6mA), a non-canonical DNA modification present at low levels in eukaryotes, has emerged as an important epigenetic marker (Liang et al., 2018a). In prokaryotes, 6mA regulates DNA replication and repair, virulence, and gene expression (Wright et al., 1997; Julio et al., 2001; Kahng and Shapiro, 2001; Reisenauer and Shapiro, 2002). In eukaryotes, the genome-wide distribution and function of 6mA were largely unknown until recent reports on unicellular green algae (Fu et al., 2015), fungi (Mondo et al., 2017), animals

Published by the Plant Communications Shanghai Editorial Office in association with Cell Press, an imprint of Elsevier Inc., on behalf of CSPB and CEMPS, CAS.

This is an open access article under the CC BY license (http://creativecommons.org/licenses/by/4.0/).

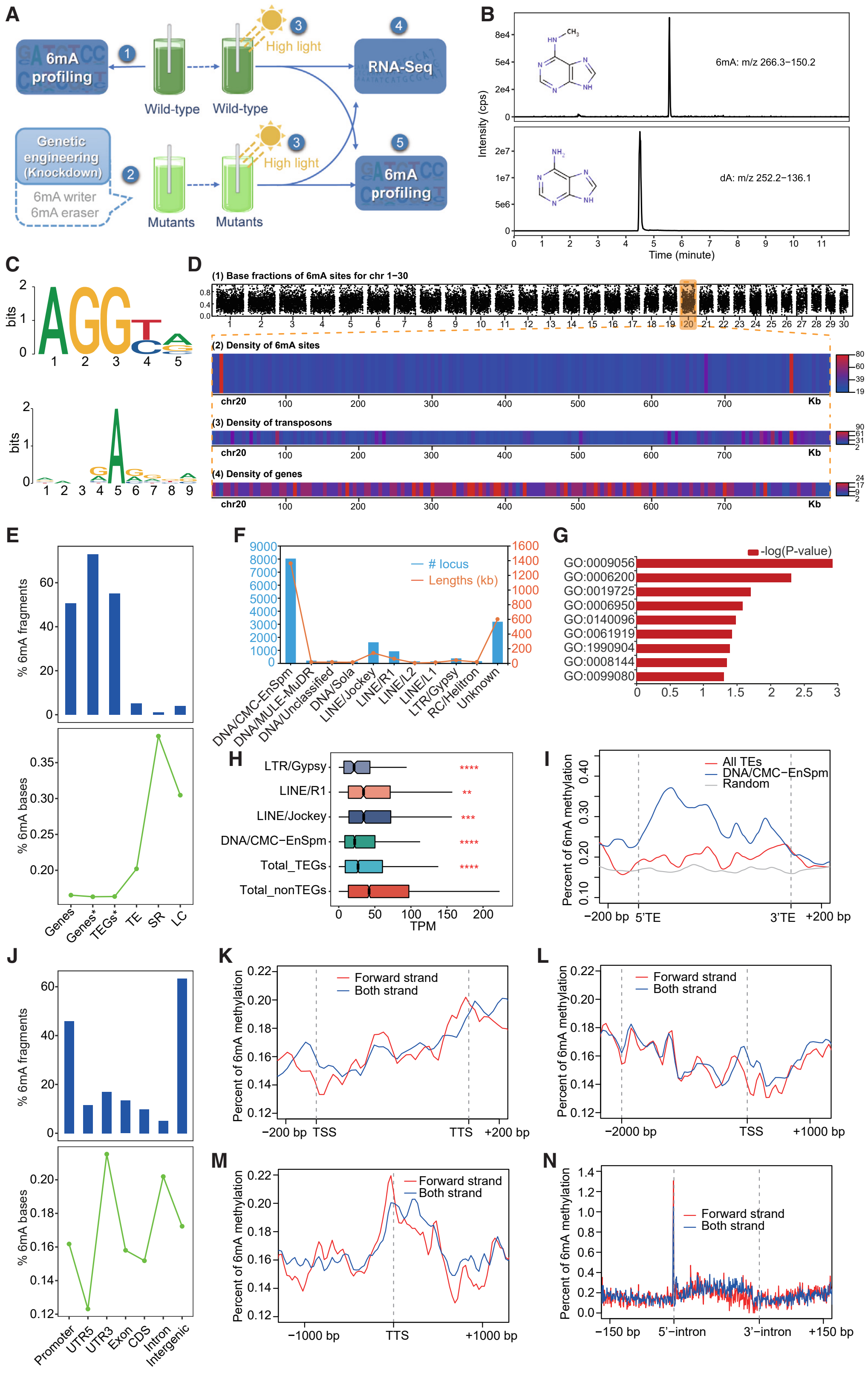

Figure 1. Distribution and global features of 6mA events in the *N. oceanica* genome

(A) Experimental design of this study.

(B) Mass spectra of 6mA and dA in isolated *N. oceanica* genomic DNA as detected by UPLC–MS/MS.

(legend continued on next page)

(Greer et al., 2015; Zhang et al., 2015; Wu et al., 2016; Wang et al., 2017; Yao et al., 2017; Ma et al., 2018), and plants (Zhang et al., 2018; Zhe et al., 2018; Zhou et al., 2018). The functions of 6mA appear to be quite divergent among eukaryotes. For example, 6mA is enriched around transcription start sites (TSSs) and its association with active transcription is conserved among *Chlamydomonas*, early-diverging fungi, and *Arabidopsis* (Fu et al., 2015; Mondo et al., 2017; Zhe et al., 2018). However, in rice, N6-methylated As in gene bodies can activate gene transcription, but those in promoter regions are involved in gene silencing (Zhang et al., 2018; Zhou et al., 2018); in *Tetrahymena*, 6mA is associated with genes transcribed by RNA polymerase II but is not correlated with active transcription (Wang et al., 2017). Another key role of 6mA is activation or repression of transposable elements (TEs), e.g., in *Drosophila* (Zhang et al., 2015) and mouse (Wu et al., 2016; Yao et al., 2017). Notably, green algae (*Chlamydomonas*), ciliates (*Tetrahymena*), and early-diverging fungi, which contain the AMT1 clade of MT-A70 methyltransferases (Wang et al., 2019), are the only species that show symmetrical distribution of 6mA at ApT dinucleotides (Fu et al., 2015; Mondo et al., 2017), suggesting the functional divergence of 6mA in unicellular eukaryotes.

Microalgae are diverse, unicellular, photosynthetic organisms that are responsible for one-half of global photosynthetic activity and primary production (Singh and Saxena, 2015). 6mA and 5mC have been found in the green algae *Chlamydomonas reinhardtii* (Hattman et al., 1978) and *Volvox carteri* (Babinger et al., 2001), but a whole-genome 6mA map is available only for *C. reinhardtii*, in which 6mA marks active TSSs (Fu et al., 2015). It is not clear whether patterns of 6mA are conserved in microalgal genomes, how such patterns are linked to gene regulation, or to what degree organismal phenotypes are altered by 6mA. Efforts to tackle these questions have been hindered by the genomic coexistence of 5mC, whose TE-silencing activities in animals, plants, fungi, and algae (such as diatoms) can mask or obscure the roles of 6mA (Deniz et al., 2019).

Nannochloropsis spp. are industrial feedstocks and leading research models for microalgal oil production (Dong et al., 2013; Li et al., 2014; Xin et al., 2017, 2019). Intriguingly, their genomes lack 5mC owing to the absence of DNA (cytosine-5) methyltransferases (Fan et al., 2020), suggesting that they may serve as exceptional models for the study of 6mA function. Here, we used the industrial photosynthetic oleaginous microalga *N. oceanica* as a model and produced a genome-wide map that reveals the distribution of 6mA on each chromosome and suggests its mode of transcriptional regulation. Two key genes controlling 6mA epigenomic modifications were identified and their functions in biomass productivity and lipid metabolism were demonstrated through knockout experiments. These findings lay a foundation for epigenetic engineering of industrial microalgae.

RESULTS

Creating a genome-wide 6mA map for *N. oceanica*

To examine the function of DNA methylation in *N. oceanica*, we employed a strategy that integrated genome-wide 6mA profiling and genetic perturbation of the 6mA machinery (Figure 1A). We started by improving the *N. oceanica* IMET1 genome assembly using ∼340× genome coverage of PacBio single-molecule real-time (SMRT) sequencing data and further scaffolding with additional Hi-C data (Gong et al., 2020) (Supplemental methods). This produced a high-quality reference genome with chromosome-scale assembly and superior base completeness (IMET1v2; details in the Supplemental results, Supplemental Table 1–4, and Supplemental Figure 1).

We next produced a genome-wide, single nucleotide resolution map of 6mA in *N. oceanica* by kinetic analysis of the SMRT data. Approximately 0.1% of the adenines were N6-methylated (6mA/A), a lower percentage than that reported for *C. reinhardtii* (Fu et al., 2015). Liquid chromatography tandem mass spectrometry (LC–MS/MS) is another approach for detecting and quantifying DNA methylation (Liang et al., 2018b). To further confirm the presence and level of 6mA in the *N. oceanica* genome, we detected 6mA and dA by an LC–MS/MS assay and quantified them using standard curves derived from 6mA and dA reference compounds. Clear peaks for 6mA and dA were observed in the corresponding mass spectra (Figure 1B), confirming the presence of 6mA in the *N. oceanica* genome. 6mA nucleotides accounted for 0.4% of all As (Figure 1B), again supporting the low abundance of 6mA in *N. oceanica*.

Motif mining of the SMRT data revealed preferences in 6mA positioning: (*i*) there were conserved guanine (G) bases next to 6mA sites (as shown in the sequence logo in Figure 1C); and (*ii*) AGGYV was the most abundant 6mA-associated motif in *N. oceanica* (Figure 1C and Supplemental Figure 2), similar to those of *C. elegans* (AGAA and GAGG) (Greer et al., 2015), *A. thaliana* (ANYGA, GAGG, and ACCT) (Zhe et al., 2018), and rice (AG and

(C) The motifs and sequence logo (bottom) that are enriched around 6mA sites (9mers). 6mA sites occur most frequently at GAGG motifs (top).
(D) The frequencies of 6mA sites, TEs, and genes along the chromosomes suggest 6mA site preferences. Details for chromosome 20 are shown as an example. The heatmaps were drawn in 5-kb bins.
(E) Proportions and base proportions of 6mA events for different genomic regions (strand specific). Genes, gene body; Genes*, gene body and 2-kb promoter region; LC, low-complexity fragments; TEGs*, Genes* that overlap with TEs.
(F) Counts and lengths of different TE superfamilies in the *N. oceanica* genome.
(G) Significant GO terms for CMC-EnSpm-associated genes (EnSpm-TEGs).
(H) Association between TEGs and gene transcription implies that TEs such as DNA/CMC-EnSpm and LTR/Gypsy have a repressive effect. Asterisks indicate significance based on the Wilcoxon test (**$p < 0.01$, ***$p < 0.001$, ****$p < 0.0001$).
(I) 6mA occupancy around transposons.
(J–N) Percentage of 6mA fragments (top) and percentage of 6mA bases (bottom) for various types of genomic regions (strand specific). Also shown are the 6mA occupancy around the gene body **(K)**, TSS **(L)**, TTS **(M)**, and introns **(N)**. For each gene, the gene body was consolidated into 1000 bp, and the intron was consolidated into 200 bp. The intron strand was defined as the strand of the same gene.

GAGG) (Zhou et al., 2018) but very distinct from that of *C. reinhardtii* (AT) (Fu et al., 2015). Thus, the base context of 6mA in *N. oceanica* appears more similar to that of higher plants than to that of green algae.

The map also reveals that (Figure 1D): (*i*) 6mA is widely distributed across the nuclear genome (Supplemental Figure 1), with the fraction of methylation typically ranging from 10% to 80% for each individual adenine; and (*ii*) the density of 6mA sites fluctuates across chromosomes. In the 5-kb resolution map, no strong correlation was observed between the density of 6mA sites and that of genes or transposons. However, in the single-base resolution map, we discovered regions with higher 6mA levels. Approximately 78% of the intervals between adjacent 6mA sites were <2 kbp (Supplemental Figure 3), and 2253 of the 6mA sites (9.2% of all 6mA sites) formed 126 densely methylated adenine clusters ("6mA hotspots"). Approximately 15% of these 6mA hotspots (19 of 126) were associated with TEs (vs. 9.8% on average; $p < 0.05$; permutation test), suggesting that 6mA hotspots are positively correlated with TEs. Thus, it appears that 6mA hotspots are selectively distributed in the *N. oceanica* genome and may have specific functions.

Positional preferences of 6mA events in the *N. oceanica* genome

To further characterize 6mA site preferences, we examined various genomic features in a strand-specific (Figure 1E) and non-strand-specific manner (Supplemental Figure 4). In terms of the number of methylated regions (Figure 1E, top), ~51% of protein-coding genes and 55% of transposable-element-associated genes (TEGs) were marked by at least one 6mA event, whereas repetitive elements, including TEs, simple repeats (SRs), and low-complexity sequences (LCs), were less prone to 6mA modification (Z-test, $p < 0.001$; e.g., only 5% of TEs were marked by 6mA). The density of 6mA-methylated bases (6mA/A) was much higher for repetitive elements (TEs, SRs, and LCs) than for protein-coding genes and TEGs (>0.20% vs. ~0.16%) (Figure 1E, bottom).

TEs, which were widely distributed in the *N. oceanica* genome, were associated with 4361 genes (i.e., TEGs; 42.2%), and DNA/CMC-EnSpm (the CMC-EnSpm superfamily of DNA transposons) (Wei et al., 2016) accounted for ~60% of the TEs (Figure 1F). Alignment of the TE and TEG distribution patterns revealed that TEs were inserted into both intragenic and intergenic positions of TEGs. There were 2420 TEs in coding sequences (CDSs) and 2553 TEs that overlapped with both introns and CDSs (Supplemental Figure 5); the latter may contribute to the functional evolution of TEGs. TEGs that were associated with DNA/CMC-EnSpm (EnSpm-TEGs) were enriched in Gene Ontology (GO) terms like catabolic process (GO:0009056), cellular homeostasis (GO:0019725), response to stress (GO:0006950), and others (Figure 1G). TEGs containing TEs from different superfamilies differed in transcription level (e.g., Wei et al., 2016), and the lowest transcription was observed for those containing DNA/CMC-EnSpm and LTR/Gypsy TEs (Wilcoxon test, $p < 0.001$) (Figure 1H). Three hundred and ninety-nine EnSpm-TEGs (13.0%) were differentially expressed under nitrate limitation (178 up- and 155 downregulated), a significantly higher percentage than the overall ratio (11.5%, 583 up- and 607 downregulated; binomial test, $p < 0.01$) (Supplemental Figure 5). Likewise, 352 EnSpm-TEGs (13.8%) were differentially expressed under CO_2 limitation (297 up- and 55 downregulated), also a significantly higher percentage than the overall ratio (11.4%; 825 up- and 349 downregulated; binomial test, $p < 0.001$) (Supplemental Figure 5). 6mA-methylated bases (6mA/A) were enriched in TEs, especially DNA/CMC-EnSpm, compared with randomly selected genomic regions (similar to *Drosophila*; Zhang et al., 2015) (Figure 1I), suggesting that 6mA may contribute to the inhibition of TEG expression.

Genes consist of multiple regions, including promoters, introns, untranslated regions (UTRs), and exons. Comparison of the ratios of methylated regions (with at least one 6mA event) revealed higher 6mA occurrence in the promoter and intergenic regions than in exons or introns (Figure 1J, top). Based on the density of 6mA (defined as 6mA/A), 3′ UTR regions were much more methylated than promoters, introns, and exons (Figure 1J, bottom) (Z-test, $p < 0.001$), implying a potential role for 6mA in termination of transcription. Notably, 6mA density was similar in exons and introns (with introns being slightly higher) (Figure 1J), in contrast to *A. thaliana* and rice, in which most 6mA sites are found in exons (Supplemental Figure 6). The positional distribution of 6mA along the gene structure revealed that: (*i*) 6mA density increased gradually along the gene body (Figure 1K); (*ii*) the region around the TSS had the lowest 6mA density (Figure 1L); (*iii*) a prominent peak of 6mA density was found around the transcription termination site (TTS) but not the TSS (Figure 1M); and (*iv*) in introns, at least six times more 6mA modifications were located right before the splicing donor site (1–2 bp upstream) (Figure 1N), as also observed for *A. thaliana* and rice but not for *C. reinhardtii* (Supplemental Figure 6). However, unlike in *A. thaliana* and rice, the density of 6mA in *N. oceanica* was higher along introns than in the surrounding exon area (Supplemental Figure 6). This evidence, especially the marked abundance of 6mA around the TTS and 5′ intron, suggests roles for 6mA in transcription termination and co-transcriptional splicing.

The 6mA pattern of the *N. oceanica* genome is thus characterized by (*i*) no enrichment of 6mA in GATC motifs, unlike that of *C. reinhardtii* (Fu et al., 2015) and fungi (Mondo et al., 2017), and (*ii*) no enrichment of 6mA around the TSS or within exons, unlike that of rice (Zhou et al., 2018) and *A. thaliana* (Zhe et al., 2018). Regulation of gene transcription by 6mA in *N. oceanica* is likely distinct from that in these model organisms.

Functional preference of 6mA events in the *N. oceanica* genome

To probe the functional consequences of 6mA modification, we correlated the presence and density of 6mA with those of various genome-encoded functional elements in *N. oceanica*. In non-coding genes, seven 6mA sites were found in four 28S rRNA genes and two 18S rRNA genes, suggesting a role for 6mA in RNA polymerase I–mediated gene transcription. However, no 6mA sites were found in genomic sequences of tRNAs and 5S rRNA, implying that 6mA is not involved in RNA polymerase III–mediated gene transcription. These observations appear to differ

from those in other organisms such as *A. thaliana*, *C. elegans*, *Drosophila melanogaster*, and *Homo sapiens* (Li et al., 2020).

We next categorized 6mA-methylated and unmethylated genes using Clusters of Orthologous Genes (COGs) and GO terms. The two COG categories "replication, recombination and repair" and "signal transduction mechanisms" were enriched in 6mA-methylated genes (Figure 2A) (binomial test, adjusted $p < 0.01$). Notably, in the COG categories, the average gene lengths showed strong positive correlations with the ratios of 6mA-methylated genes (Pearson correlation = 0.77; $p < 0.001$). Moreover, the density of 6mA (6mA/A), 0.17% ± 0.02%, was quite similar among the different COG categories, with no outliers detected (Grubbs test, $p > 0.05$).

No GO terms were enriched in genes methylated in the gene body, promoter, exon, 5′ UTR, 3′ UTR, or intron region. However, twelve GO terms were enriched in CDS-methylated genes (false discovery rate [FDR] <0.01). These included the cellular component nucleus (GO:0005634), nucleic acid and RNA metabolic processes (GO:0090304 and GO:0016070), and biological processes related to nucleotide/ribonucleotide binding (GO:0032553, GO:0017076, GO:0030554, and others) (Figure 2B). Comparison of the gene sets associated with these enriched GO terms revealed that nine molecular function GO terms were associated with similar gene sets and shared 112 of the same genes (blue rows; Figure 2C). Thus, for these specific GO categories, marking of 6mA in the CDS region is conserved among selected genes. Nucleotide/ribonucleotide binding and metabolism in the nucleus are fundamental eukaryotic functions. In rice, genes associated with "nucleolus", "nuclear lumen", and "nucleotide binding" were enriched in 6mA in gene body regions (Zhou et al., 2018). Thus, the enrichment of these specific functions for CDS-marked genes in *N. oceanica* indicates that 6mA may have an ancient origin and is an epigenetic marker shared by eukaryotes.

Genome-wide positive correlation of 6mA with gene transcription level

To determine whether and how 6mA regulates gene function, we used RNA sequencing (RNA-seq) to test the genome-wide correlation between 6mA and gene transcription (quantified as transcripts per million [TPM]). The proportion of unmethylated genes with low expression was 75% higher than that of 6mA-methylated genes with low expression (14.2% vs. 8.1%; low expression defined as TPM $\leq$1.0) (Figure 3A), suggesting an association between the presence of 6mA and active transcription. At the global scale, within the gene body and TTS (defined as ±500 bp), 6mA-methylated genes exhibited higher expression levels than unmethylated genes (Wilcoxon test, $p < 0.001$) (Figure 3B); however, there was no such difference for TSSs (defined as –300 bp to +100 bp) (Figure 3B). Furthermore, scanning the 6mA occupancy along genes and their 3′ UTRs revealed that highly expressed genes (TPM >100) harbored ~24% more 6mA sites in the gene body than genes with lower expression (TPM <100) (Figure 3C and Supplemental Figure 7). Therefore, 6mA is positively correlated with gene expression levels in *N. oceanica*. Notably, such a correlation does not seem to preferentially target any specific functional categories of protein-coding genes, as in each of the COG categories, the expression levels of 6mA-methylated genes and un-methylated genes (at the gene body, TSS, TTS or CDS) showed no significant differences (Wilcoxon test, adjusted $p > 0.05$), i.e., the association between 6mA and transcription is a collective effect of all genes.

The contribution of DNA 6mA accumulation in transposons and 3′ UTRs to active gene expression (TPM >1) was also examined. The transcript levels of active genes with 6mA-marked 3′ UTRs (UTR3+) was significantly higher than that of genes without 6mA in the 3′ UTRs (UTR3–) (Figure 3D) (Wilcoxon test, $p < 0.0001$). This was expected, because the 3′ UTR is part of the gene body and 6mA in the gene body is positively associated with transcription. However, the transcript levels of active TEGs with 6mA-marked TEs (TE+) were significantly lower than those of TEGs without 6mA in TEs (TE–) (Figure 3D) (Wilcoxon test, $p < 0.0001$), similar to results in *Drosophila* (Zhang et al., 2015), implying that 6mA may repress TE activity in *N. oceanica*.

Intriguingly, despite the slightly lower 6mA level of TEGs than non-TEGs (Figure 1E), the proportion of TEGs with 6mA-marked promoters or gene bodies was similar to that of non-TEGs (Figure 3A). Moreover, (*i*) TEGs with 6mA marks only in the promoter (Promoter) showed higher average expression levels than un-methylated genes (None) (Wilcoxon test, $p < 0.05$), but this was not true for non-TEGs (Wilcoxon test, $p > 0.05$); (*ii*) for both TEGs (Wilcoxon test, $p < 0.01$) and non-TEGs (Wilcoxon test, $p < 0.01$), genes with 6mA marks in the gene body (Body and Both) showed higher average expression levels than un-methylated genes (None); and (*iii*) for non-TEGs, the Body genes showed higher average expression levels than the Promoter genes (Wilcoxon test, $p < 0.001$) (Figure 3E). Thus, 6mA in the gene body is likely to be the main contributor to activation of gene transcription.

The positive correlation between 6mA in the gene body (both presence and density) and gene transcript level in *N. oceanica* is similar to that in rice (Zhou et al., 2018) and is probably conserved in additional evolutionary branches as well. By contrast, 6mA in *C. reinhardtii* is associated with the TSSs of active genes, indicating a role in the regulation of nucleosome positioning around the TSS (Fu et al., 2015). Therefore, considering the enrichment of 6mA around the TTS and 5′ intron regions in *N. oceanica*, 6mA seems to play multiple regulatory roles, not only in transcription termination and co-transcriptional splicing but also in transcript elongation.

Identification and validation of genes underlying DNA 6mA methylation in *N. oceanica*

To pinpoint the functional mechanism of DNA 6mA methylation in *Nannochloropsis* spp., we identified candidate genes encoding a 6mA "writer" and "eraser". We proposed the *N. oceanica* gene NO08G00280, which encodes an S-adenosyl-L-methionine-dependent methyltransferase, as a candidate DNA 6mA methyltransferase gene because of its homology to human N6AMT1, which was functionally validated as a DNA 6mA methyltransferase in human (Xiao et al., 2018). A phylogenetic analysis of putative and validated N6AMT1 enzymes from various organisms, including *Nannochloropsis*,

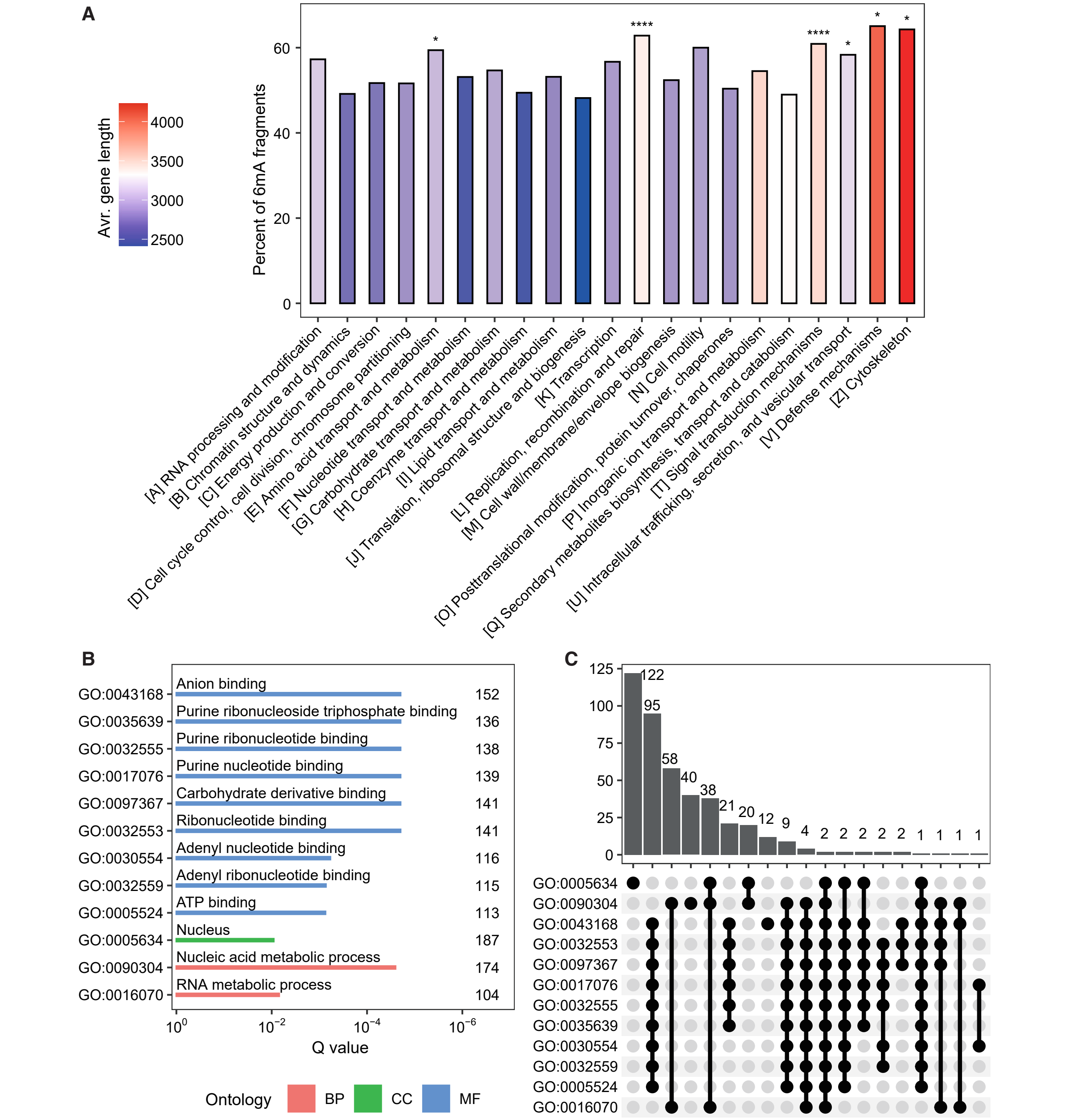

Figure 2. Functional preference of DNA 6mA loci in the *N. oceanica* genome

(A) Percentages of 6mA-methylated fragments for genes in various COG categories. Asterisks indicate significance based on the binomial test (*$p < 0.05$, ****$p < 0.0001$).

(B) Each of the GO terms that are enriched for genes with 6mA marks in their CDS regions.

(C) Overlaps of genes among the gene sets associated with the GO-term enrichment in **(B)**. A black dot indicates the presence of overlap between or among the gene sets associated with a particular GO enrichment.

Chlamydomonas, *Arabidopsis*, and human, revealed significant conservation of their protein sequences (Figure 4A, 4B, and Supplemental Table 5). However, this analysis was unable to establish a clear link between N6AMT1 and observed 6mA motifs (Figures 4A and 4B). These results suggested that N6AMT1 may not be the primary contributor to 6mA methylation in most organisms. Nonetheless, the presence of a similar GAGG-like 6mA motif in *N. oceanica*, human, and plants suggested that N6AMT1 and, in this case NO08G00280, was a promising 6mA methylase candidate.

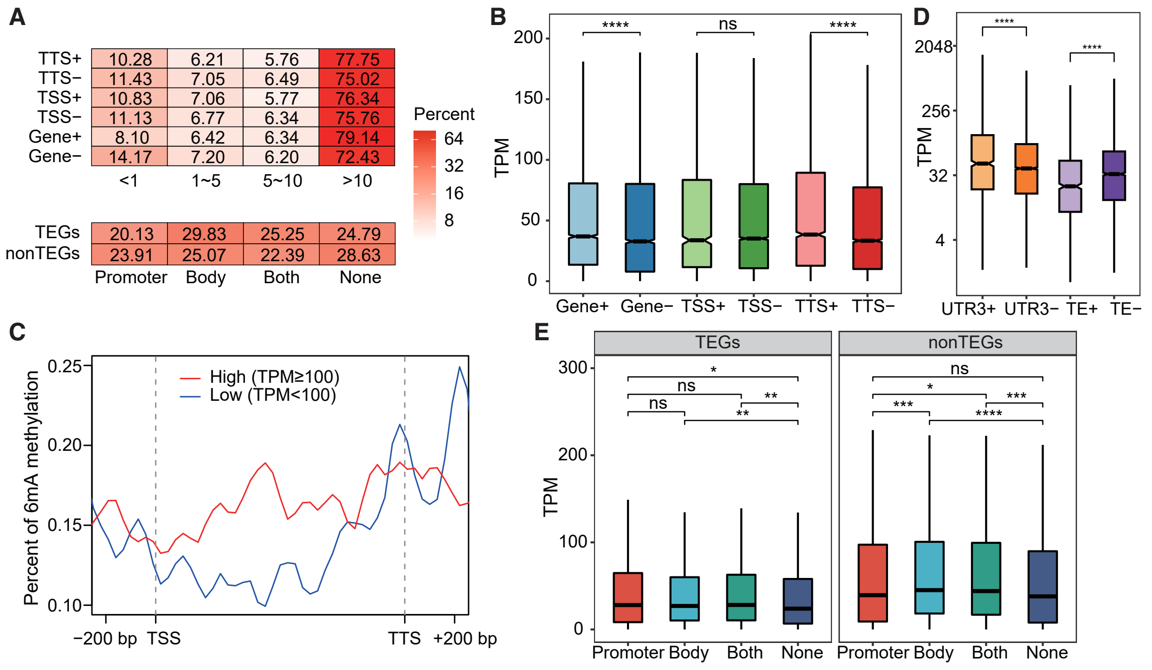

Figure 3. DNA 6mA is associated with gene transcription in *N. oceanica*

(A) Percentages of methylated genes (Gene+), unmethylated genes (Gene–), and genes methylated around the TSS (300-bp upstream and 100-bp downstream; TSS+/TSS–) and TTS (±500 bp; TTS+/TTS–) at a given TPM level (≤1.0, 1–5, 5–10, and ≥10). The number of N6-methylated genes with expression levels below the TPM value of 1.0 (silent genes) was about one-half that of un-methylated silent genes. The percentages of TEGs and non-TEGs with 6mA marks in the promoter, gene body, and both are also shown below.

(B) Transcription comparison for Gene+, Gene–, and genes N6-methylated near the TSS (TSS+ vs. TSS–) or TTS (TTS+ vs. TTS–).

(C) 6mA occupancy along genes (strand specific) for highly expressed genes (High, TPM >100) and genes with low expression (Low, TPM ≤100).

(D) Comparisons between the transcription level of active TEGs (TPM >1) with 6mA-marked TEs ("TE+") and those without 6mA-marked TEs ("TE–"; "TE+" vs. "TE–") and active genes (TPM >1) with 6mA-marked 3′ UTRs ("UTR3+") and those without 6mA in their 3′ UTRs ("UTR3–"; "UTR3+" vs. "UTR3–").

(E) Expression levels of genes marked by 6mA only in the promoter, only in the body, or in both compared with the average expression levels of the TEGs (left) or non-TEGs (right). The *y* axis shows the average expression level (TPM). Asterisks indicate significance based on the Wilcoxon test (ns, $p > 0.05$; *$p < 0.05$; **$p < 0.01$; ***$p < 0.001$; ****$p < 0.0001$).

We further hypothesized that the AlkB family gene NO06G02500 was involved in 6mA demethylation, as its encoded protein is a homolog of rice OsALKBH1 (Zhou et al., 2018) and human ALKBH1 (Liu et al., 2016; Xiao et al., 2018; Zhang et al., 2020), which have been validated as 6mA demethylases by *in vivo* and *in vitro* experiments. In time-series transcriptomic profiles of *N. oceanica* under nitrogen depletion (Li et al., 2014) and CO_2 depletion (Wei et al., 2019), NO08G00280 and NO06G02500 transcripts both exhibited constitutive expression, but their temporal patterns were distinct (Figures 4C and 4D). In general, expression of both genes first decreased and then increased under stress. However, under nitrogen depletion (N+/N–), NO08G00280 expression decreased further at 48 h (Figure 4C). By contrast, under CO_2 depletion (HC/VLC), NO08G00280 expression increased from 6 h onwards, but NO06G02500 expression increased only after 12 h (Figure 4D). These observations were consistent with potential roles for NO08G00280 and NO06G02500 in regulating DNA 6mA methylation in response to stress.

To test the role of NO08G00280 as a DNA 6mA methyltransferase, we investigated its effect on the 6mA level of *N. oceanica* genomic DNA *in vitro* and *in vivo*. For the *in vitro* test, the cDNA of NO08G00280 was ligated into the pGEX plasmid, and recombinant GST-NO08G00280 was purified. In an *in vitro* methylation assay, a synthesized NC-oligo (Xiao et al., 2018) was methylated by the purified recombinant GST-NO08G00280, and the methylated DNA was detected and quantified by dot blotting with an anti-6mA antibody. The dot blot result showed that GST-NO08G00280 increased the 6mA level of synthetic oligonucleotide substrates (NC-oligo) by ∼1.5-fold (Xiao et al., 2018) (Figure 5A). Notably, the dA methylation activity of NO08G00280 was not particularly high, consistent with the low abundance of 6mA in *N. oceanica*. To test its role as a DNA 6mA methyltransferase *in vivo*, NO08G00280 was knocked out by CRISPR–Cas9 to produce mutants M1 and M2 (Figure 5B). High light, which enhances lipid accumulation in wild-type (WT) *Nannochloropsis* spp. (Alboresi et al., 2016; Ma et al., 2016; Huete-Ortega et al., 2018; Han et al., 2020), was used to test potential links among the knockout genotype, altered 6mA patterns, and changes in gene expression. After 7 days of high-light culture, three replicate samples of M1, M2, and WT *N. oceanica* were obtained, and genome-wide 6mA events were profiled using the PacBio Sequel II platform (>100× sequencing depth per sample).

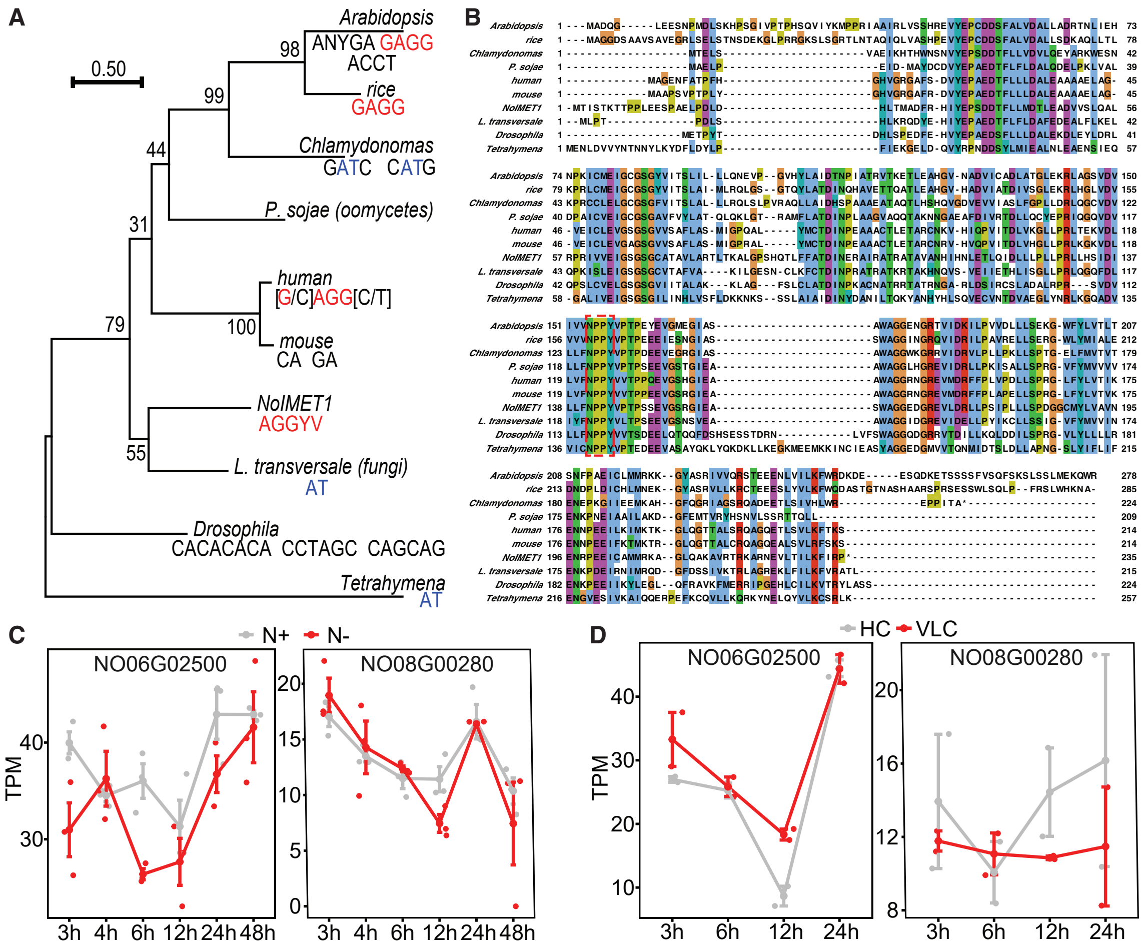

Figure 4. Discovery and validation of enzymes involved in DNA 6mA methylation in *N. oceanica*

(A) Phylogenetic tree of DNA 6mA methyltransferases constructed using MEGA X with the maximum likelihood method. The sequences used include all those present in the alignment in **(B)**. The consensus 6mA motifs identified in each organism are mapped onto the phylogenetic tree.

(B) Multiple protein sequence alignment of putative DNA 6mA methyltransferases.

(C) Expression of DNA-6mA-associated genes during nitrogen depletion. The DNA 6mA methyltransferase NO08G00280 was downregulated at 48 h.

(D) Expression of DNA-6mA-associated genes during CO_2 depletion. Expression of the DNA 6mA demethylase NO06G02500 continued to decline at 6–12 h of CO_2 depletion, but that of the DNA 6mA methyltransferase of NO08G00280 had begun to increase by that time frame.

The mutants and WT had equivalent global 6mA levels, indicating the presence of additional DNA 6mA methyltransferases in *N. oceanica* (Supplemental Figure 8). However, comparison between M2 and WT revealed that 19.5% of the 6mA-marked genes (i.e., those with at least one 6mA site in all replicates of M2 or WT; 225 of 1152) were shared between M2 and WT; 15.6% of them showed changes in 6mA levels (fold change >2) in M2 relative to WT, with 7.8% showing increased 6mA levels. In addition, 80.5% of the 6mA-marked genes changed methylation state from carrying at least one 6mA site to none (WT to M2, 43.7%) or vice versa (WT to M2, 36.8%). No significant differences in the 6mA distribution pattern along genes were detected between M2 and WT (Supplemental Figure 8). These observations suggest a large-scale alteration of genome-wide 6mA patterns due to knockout of NO08G00280.

In terms of the microalgal phenotype, mutant growth (biomass accumulation) was 13% lower than WT growth (*t*-test, $p < 0.05$) (Figure 5C). Similarly, lipid content was 14.3% lower in M1 and 16.2% lower in M2 (Figure 5D) than in the WT. Gas chromatography–mass spectrometry (GC–MS) revealed that the fatty acid content was 24.8% lower in M1 and 24.7% lower in M2, and triacylglycerol (TAG) content was 26.8% lower in M2 (Figure 5E). However, the percentage of polyunsaturated fatty acids (PUFAs) was significantly higher in the mutants than in the WT (34.4% and 81.4% increases in C18:2, 38.1% and 17.8% increases in C20:4, and 42.7% and 44.0% increases in C20:5 in M1 and M2, respectively) (Figure 5F). Thus, 6mA is important for growth and lipid metabolism in *N. oceanica*.

To probe the underlying mechanism, RNA-seq for the WT and mutants was performed for the same samples in biological triplicates (which showed excellent reproducibility) (Supplemental Figure 9). Compared with the WT, the NO08G00280-knockout mutants (M1 or M2) contained 600 differentially expressed genes (DEGs) (Supplemental File 1). Specifically, expression levels of Rubisco (Vieler et al., 2012) and almost all light-harvesting complex (LHC) genes were downregulated in M1 and M2 (Figure 5G) (Supplemental File 1), consistent with the slower growth phenotype. Expression of the lipid droplet

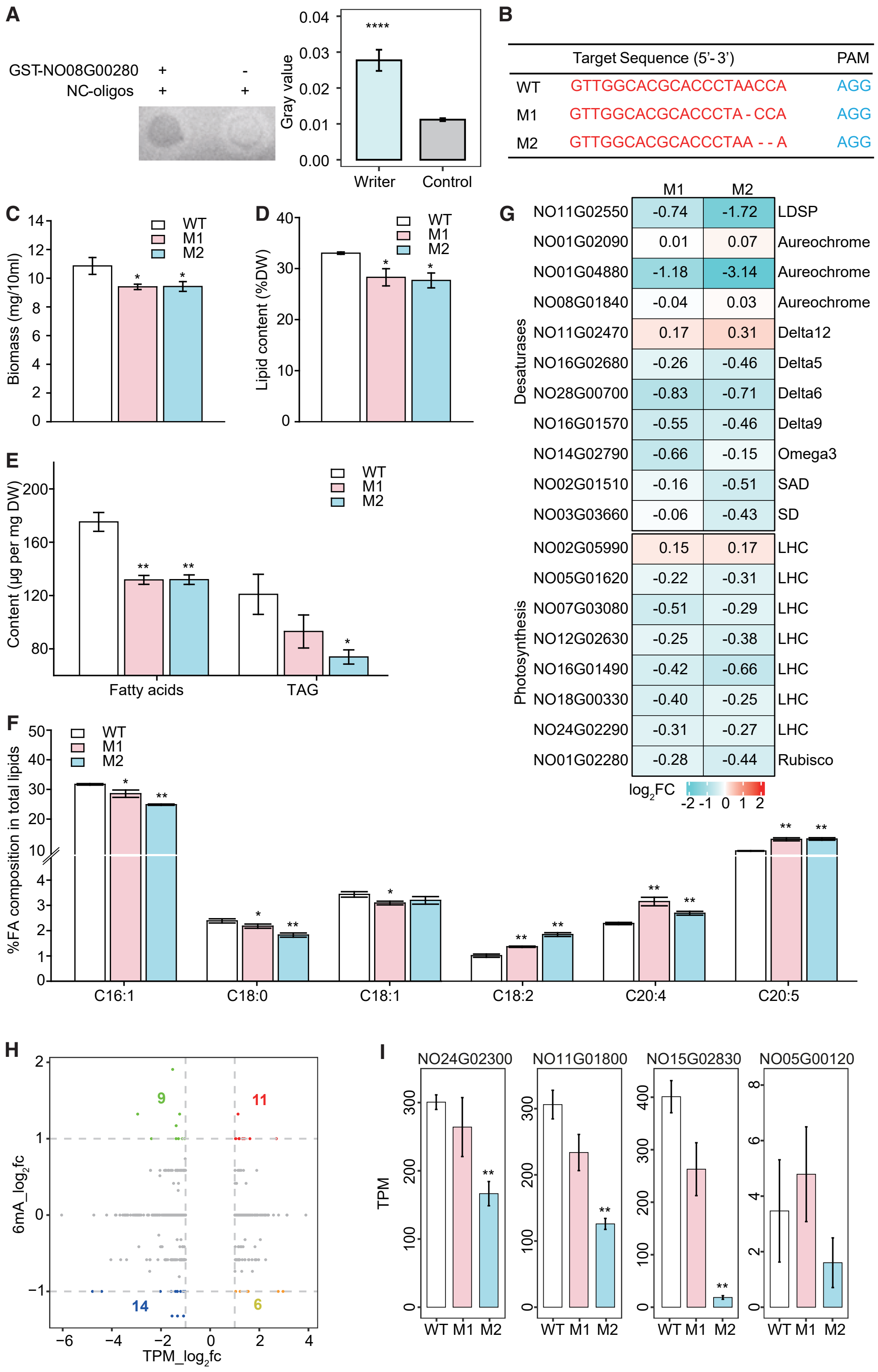

Figure 5. Changes in *N. oceanica* phenotypes resulting from knockout of NO08G00280, a putative DNA 6mA methyltransferase

(A) The recombinant GST-NO08G00280 protein slightly methylated DNA oligos in an *in vitro* methylation reaction as revealed by dot blotting assays. The gray values of randomly selected reaction areas were compared (Wilcoxon test, ****p < 0.0001).

(legend continued on next page)

(LD) surface protein gene NO11G02550, which encodes a major LD-associated protein that serves as a marker of TAG accumulation and LD dynamics in *N. oceanica* (Vieler et al., 2012; Zienkiewicz et al., 2020), was also downregulated in M1 and M2, consistent with the phenotype of reduced TAG contents. Also downregulated was NO01G04880, which encodes an aureochrome, a stramenopile-specific transcription factor with a bZIP DNA-binding motif (Takahashi et al., 2007). We recently showed that another *N. oceanica* aureochrome (NO08G01840) is a blue light–responsive transcription factor that modulates lipid production by repressing NoDGAT2B transcription (Zhang et al., 2022). Notably, the reduction in NO01G04880 transcripts (log2-fold change) (Figure 5G) was consistent with the reduction in TAG content in the mutants (Figure 5E), suggesting a potential link between NO01G04880 and TAG production as well.

Comparison of 6mA dynamics (6mA/A, log2 scale) with differential gene expression levels (TPM, log2 scale) further supported the positive correlation between 6mA levels and gene expression in *N. oceanica*. Specifically, (*i*) among the upregulated DEGs, those with a >2-fold change in 6mA outnumbered those with a <0.5-fold change in 6mA (11 vs. 6), and (*ii*) among the downregulated DEGs, those with a <0.5-fold change in 6mA outnumbered those with a >2-fold change in 6mA (14 vs. 9) (Figure 5H).

In addition, a reduction in 6mA level was observed for many downregulated genes, e.g., NO24G02300, NO11G01800, NO15G02830, and NO05G00120 (Figure 5I) (NO08G00280-knockout mutants vs. WT). NO24G02300 encodes a protein involved in biosynthesis of the molybdenum cofactor, a compound present at the active site of many molybdenum-containing enzymes such as nitrate reductase (NR), sulfite oxidase, xanthine oxidoreductase, and aldehyde oxidase (Tejada-Jimenez et al., 2018). Indeed, we observed a strong positive correlation (Pearson correlation coefficient, R = 0.72) between transcript abundance of NO24G02300 and that of the NR gene (NO14G02460) (dropping by half for both genes; NO08G00280-knockout strains vs. WT). The reduction in NO24G02300 transcription in the mutants may therefore hinder nitrogen assimilation, helping to explain their slower growth. NO11G01800 encodes a sulfate transporter, and its transcriptional inhibition due to a reduced 6mA level may limit sulfur fixation in this microalga, also contributing to slower growth (Dong et al., 2017). NO15G02830 encodes a glycosyl transferase with a UDP-sulfoquinovose:DAG sulfoquinovosyltransferase domain (PLN02871) that participates in synthesis of sulfoquinovosyl diacylglycerols (SQDGs; sulfur-containing lipids and major components of the microalgal thylakoid membrane) (Merchant and Helmann, 2012). Thus, the reduction in NO15G02830 transcription associated with reduced 6mA modification may explain the reduction in lipids. NO05G00120 encodes a lipase, which hydrolyzes the ester bonds of lipids with a substrate preference (Long et al., 1998; Chen et al., 2020; Jithu Paul, 2020). Among the ∼40 candidate lipase genes in *N. gaditana*, at least 6 are thought to mediate translocation of EPA from polar membrane lipids to TAG during nitrogen starvation (Janssen et al., 2020). Notably, NO05G00120 was upregulated in *N. oceanica* at 12 h of nitrogen starvation (Li et al., 2014), consistent with its likely role in liberating PUFA-rich membrane lipids. Therefore, the downregulation of NO05G00120 transcripts, correlated with reduced 6mA modification, could explain the increased PUFA content in the NO08G00280-knockout strains.

In summary, knockout of the candidate DNA 6mA methyltransferase NO08G00280 resulted in large-scale alterations in 6mA levels and a cascade of transcriptomic changes. Specifically, downregulation of NO24G02300, NO11G01800, NO15G02830, and NO05G00120, which was correlated with their reduced 6mA modification, was linked to a reduction in microalgal growth and lipid accumulation. This evidence supports the identification of NO08G00280 as a writer that introduces genome-wide 6mA modifications in *N. oceanica*.

Identification and validation of a DNA 6mA demethylation gene in *N. oceanica*

To verify the functional activity of the candidate DNA 6mA demethylase, we investigated whether NO06G02500 could demethylate 6mA from DNA *in vitro* and *in vivo*. The cDNA of NO06G02500 was ligated into the pGEX plasmid, and recombinant GST-NO06G02500 was purified. In an *in vitro* demethylation assay, synthesized 6mA-oligos (Xiao et al., 2018) were demethylated by the purified recombinant GST-NO06G02500, and the reduction in methylated DNA due to demethylase activity was detected and quantified by dot blotting. The results showed that GST-NO06G02500 could directly and efficiently reduce the 6mA level of synthetic 6mA-modified oligonucleotide substrates (6mA-oligo) (Xiao et al., 2018) (Figure 6A).

To test the role of the NO06G02500 protein as a DNA 6mA demethylase *in vivo*, the NO06G02500 gene was knocked out by CRISPR–Cas9 to create the M3 and M4 mutants (Figure 6B). After 7 days of high-light culture, replicate M3, M4, and WT plants were obtained, and genome-wide 6mA events were profiled by PacBio Sequel II sequencing (>100× sequencing depth per sample). Consistent with the proposed demethylase activity of NO06G02500, global 6mA levels for both knockout mutants were increased by 21.3% ($p < 0.05$, one-tailed Wilcoxon test) (Supplemental Figure 8). Specifically, 20.9% of the 6mA-marked genes (i.e., those with at least one 6mA site in all replicates of M4 or WT; 306 out of 1464) were shared between M4 and WT; 20.3% of them showed changes in 6mA levels (fold-change >2) in M4 relative to WT, with 13.7% showing increased 6mA levels. In addition, 79.1% of the 6mA-marked genes changed methylation state from carrying at least one 6mA site to none (WT to M4, 28.8%) or vice versa (WT to M4,

(B–F) Mutant genome sequences at the gRNA target sites. Phenotypic changes under high-light conditions are shown: biomass productivity **(C)**, lipid content **(D)**, fatty acid and TAG contents **(E)**, and fatty acid composition of total lipids **(F)**.

(G) Transcription of selected genes associated with photosynthesis, CO_2 fixation, FA synthesis, desaturases, etc. (RNA-seq experiments in triplicate). Log2-fold changes in expression are shown in the heat map (red, upregulation; green, downregulation).

(H) The link between differential gene expression (TPM, log2 scale) and 6mA-level dynamics (6mA/A, log2 scale) in WT and mutants. For WT strains, WT_2 and WT_3 were used to calculate the 6mA level; for mutants, M2_1 and M2_2 were used.

(I) Expression levels of selected genes in the WT and mutants (TPM values). Error bars denote mean ± SD (in triplicate). Asterisks indicate significance

based on the *t*-test (*$p < 0.05$, **$p < 0.01$).

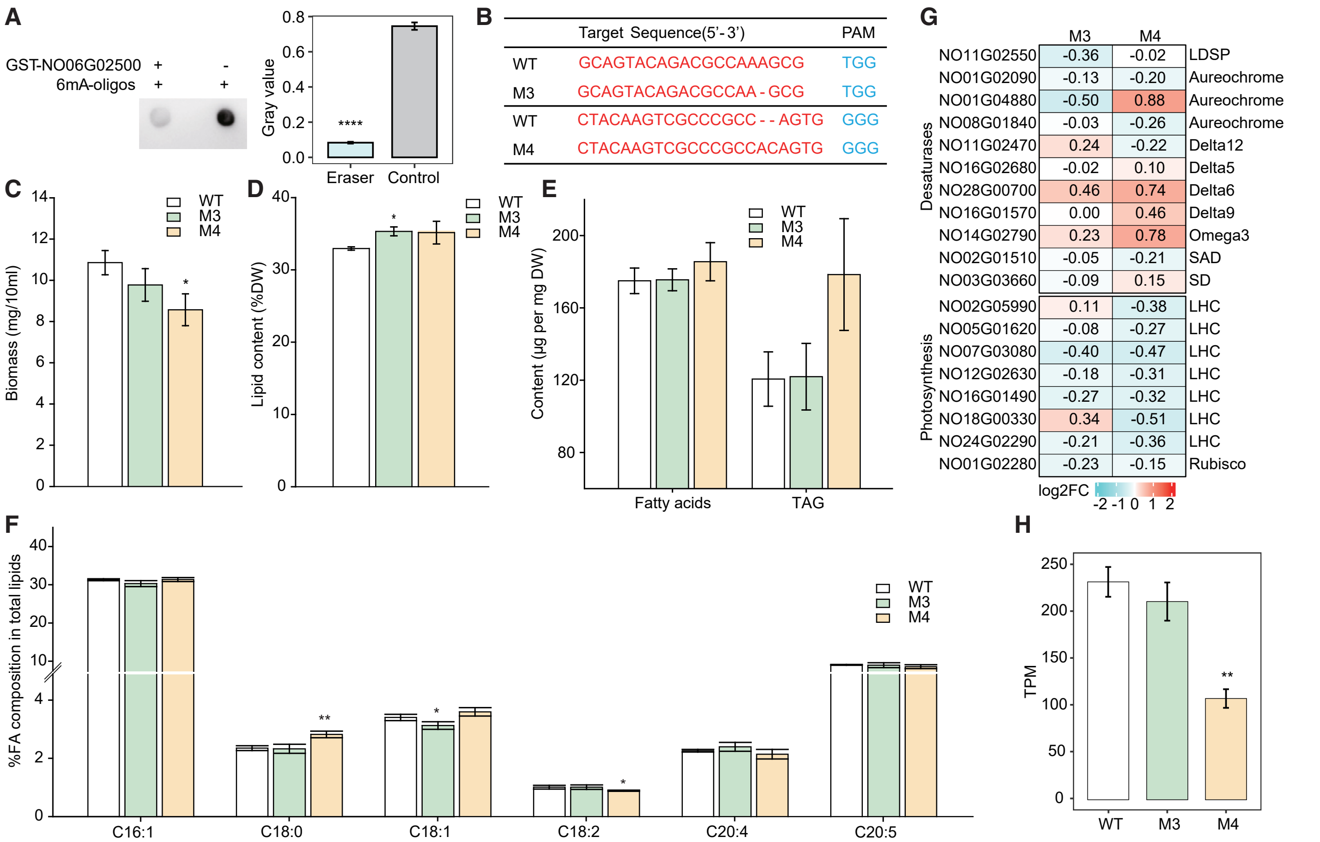

Figure 6. Changes in *N. oceanica* phenotypes resulting from knockout of NO06G002500, a putative DNA 6mA demethylase

(A) The recombinant GST-NO06G002500 protein directly and efficiently demethylated the 6mA modification in an *in vitro* demethylation reaction with 6mA-DNA oligos as the substrate. The gray values of randomly selected reaction areas were compared (Wilcoxon test; ****$p < 0.0001$).

(B) Mutant genome sequences at the gRNA target sites. Phenotypic changes under high-light conditions are shown: biomass productivity **(C)**, lipid content **(D)**, fatty acid and TAG contents **(E)**, and fatty acid composition of total lipids **(F)**.

(G) Transcription of selected genes associated with photosynthesis, CO_2 fixation, FA synthesis, desaturases, etc. (RNA-seq experiments in triplicate). Log2-fold changes in expression are shown in the heat map (red, upregulation; green, downregulation).

(H) Expression levels of selected genes in the WT and mutants (TPM values). Error bars denote mean ± SD (in triplicate). Asterisks indicate significance based on the *t*-test (*$p < 0.05$, **$p < 0.01$).

50.3%). The distribution pattern of 6mA density along genes was similar in M4 and WT, but the overall 6mA density was higher in M4 (Supplemental Figure 8).

In terms of phenotype, growth rates of M3 and M4 were 10% and 21% lower than that of the WT (Figure 6C) (*t*-test, $p < 0.05$), and lipid contents were 7.2% and 6.7% higher (Figure 6D) (*t*-test, $p < 0.05$). However, there were no significant differences between M3 or M4 and the WT in fatty acid content, TAG content, or PUFA composition (except for C18:2 which was 11.8% lower in M4) (Figure 6E and 6F). The corresponding RNA-seq data revealed that 79 genes were significantly differentially expressed in M3 or M4 compared with the WT (Supplemental File 1), 46 of which were upregulated (58.2% of DEGs).

The dynamics of 6mA modification and changes in transcript abundance were overlayed to examine their links. Expression of Rubisco and nearly all LHC genes was downregulated in M3 and M4 (Figure 6G; Supplemental File 1); consistent with this result, the 6mA levels of these genes were reduced or remained unchanged. Both 6mA level and transcript abundance were reduced for a peptide methionine sulfoxide reductase gene (NoMSR; NO21G00950) that functions in protein repair and oxidative damage protection (Tarrago et al., 2009). The downregulation of NoMSR in M3 (fold change = 0.87) and M4 (fold change = 0.47; Figure 6H) may have aggravated the level of unrepaired proteins (with changes in activity or conformation), which may have contributed to the slow growth of the mutants.

In summary, knockout of the candidate DNA 6mA demethylase NO06G02500 in *N. oceanica* resulted in a significant increase in global 6mA levels (by 21.3%), which was accompanied by growth defects. However, transcripts with changes in 6mA could be either up- or downregulated, underscoring the complexity of NO06G02500-mediated demethylation in the regulation of gene expression.

A model for epigenomic regulation of lipid accumulation in *N. oceanica*

Genome-wide profiling of 6mA sites and genetic engineering of DNA-modification machinery enabled us to propose an

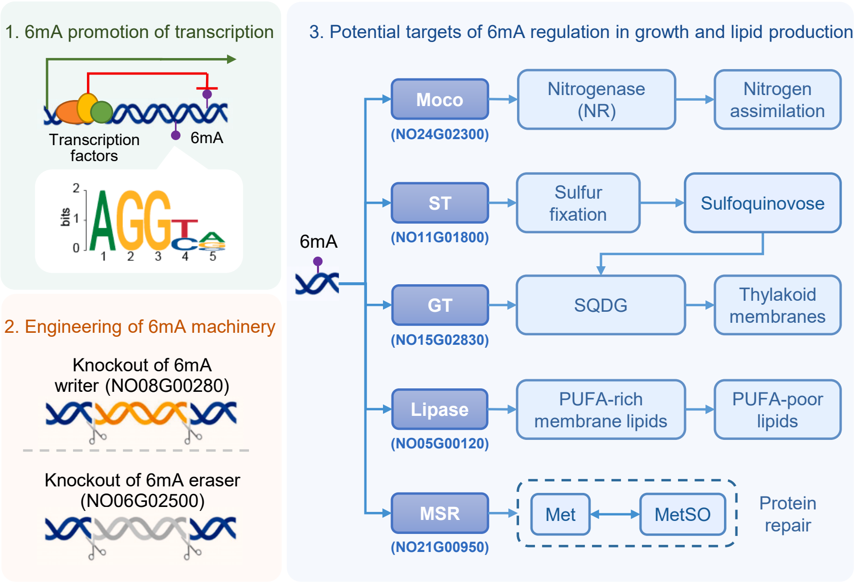

Figure 7. A mechanistic model for 6mA-mediated regulation of lipid metabolism in *Nannochloropsis* spp.
The 6mA-mediated regulation of oil production has multiple aspects, as demonstrated by the phenotypes of knockout mutants of a methyltransferase and a demethylase in the DNA 6mA machinery. These aspects include mediation of nutrient uptake and assimilation (such as nitrogen and sulfur; Moco and ST), modulation of membrane lipid composition (GT), and regulation of specific core genes (MSR). GT, glycosyl transferase; Moco, molybdenum cofactor; Met, methionine; MetSO, methionine sulfoxide; MSR, methionine sulphoxide reductase; ST, sulfate transporter.

epigenetic layer of regulation for lipid production in *N. oceanica* (Figure 7). On the basis of the positional and functional preference of 6mA localization, we showed that 6mA can promote gene transcription, perhaps by facilitating the recruitment of general transcription factors. Moreover, knockout of a methyltransferase and a demethylase in the DNA 6mA machinery disturbed the transcription of multiple genes associated with lipid production and significantly altered growth and lipid accumulation.

In this model, the epigenetic regulatory layer of oil production has three aspects: (*i*) 6mA may regulate the expression of several genes that mediate uptake and assimilation of nutrients (such as nitrogen and sulfur; NO24G02300 and NO11G01800), which not only are essential elements of the building blocks of lipids such as phospholipids, SQDGs, etc., but also affect cell growth. (*ii*) 6mA may regulate the expression of genes involved in molding the composition of membrane lipids (NO15G02830), thus further affecting the function of subcellular compartments. For example, the abundance of SQDGs in thylakoid membranes would affect cell photosynthesis, thereby linking lipid accumulation to cell growth; moreover, some lipases can mediate the conversion between membrane lipids and free neutral lipids, thus altering the properties of organelle membranes. (*iii*) 6mA may regulate certain core genes that constitute the foundation of lipid accumulation. For instance, the core gene NoMSR mediates the maintenance of protein activity, and compromised regulation by 6mA could lead to growth defects. This model therefore provides potential mechanistic links between 6mA-based transcriptional regulation and lipid production in *N. oceanica*.

DISCUSSION

A correlation between 6mA modification and gene transcription is found in eukaryotic organisms including *C. reinhardtii* (Fu et al., 2015), *A. thaliana* (Zhe et al., 2018), rice (Zhang et al., 2018; Zhou et al., 2018), and mouse (Wu et al., 2016). In *C. reinhardtii*, 6mA affects nucleosome positioning around the TSS and thus contributes to transcription initiation (Fu et al., 2015). In *N. oceanica*, after excluding the possibility of the results being skewed by bacterial contamination (Supplemental results; Supplemental Table 6), we showed that 6mA genomic distribution, enriched motifs, and association with transcription in *N. oceanica* are highly distinct from those in *C. reinhardtii* and higher plants. Enrichment of 6mA around the 5′ intron and TTS suggests roles in co-transcriptional splicing and transcription termination in *N. oceanica*. Our data support the hypothesis that 6mA regulates gene transcription by influencing the recruitment/binding of transcription-related factors (Zhe et al., 2018).

Comparison of *N. oceanica* with *C. reinhardtii* sheds light on the evolutionary conservation and the functional significance of 6mA in algae (Supplemental Table 7). In general, the 6mA level is much lower in *N. oceanica* than in *C. reinhardtii*, and their 6mA distribution patterns vary markedly. In *C. reinhardtii*, 6mA mainly resides at ApT dinucleotides around the TSS, with a bimodal distribution, and it appears to mark active genes (Fu et al., 2015). By contrast, 6mA sites in *N. oceanica* are non-canonical, mainly enriched at AGGYV motifs and not around TSSs. The most striking feature observed in *N. oceanica* was that highly expressed genes had higher 6mA levels in the gene body, a pattern distinct from that in *C. reinhardtii* but similar to that in plants. Thus, 6mA has evolved distinct functions between stramenopiles and green algae.

A comparison of 6mA in *N. oceanica* and plants also yields intriguing insights. The non-canonical localization of 6mA sites and the enrichment mostly at AGGYV motifs in *N. oceanica* are similar to those in plants (*A. thaliana* and rice). However, in contrast to observations in plants, 6mA sites are not enriched around the TSS in *N. oceanica*, and 6mA/A is higher in introns than in exons. In addition, although highly expressed genes have higher 6mA levels in the gene body in both *N. oceanica* and plants, there is no indication that 6mA in promoters marks silent genes in *N. oceanica*. Notably, despite such differences in distribution patterns among organisms, DNA 6mA is associated with active transcription for most organisms.

Knockout of the candidate methyltransferase NO08G00280 and the candidate demethylase NO06G02500 via CRISPR–Cas9 altered 6mA levels in *N. oceanica*. In the NO08G00280-knockout

mutants, the loss of 6mA was correlated with decreased gene expression, further supporting the association between 6mA and actively expressed genes. Consistent with the slow growth and altered lipid profiles of the mutants, NO08G00280 knockout led to reduced 6mA levels at the key genes NO24G02300, NO11G01800, NO15G02830, and NO14G02460, which were accompanied by reduced gene expression. Thus, 6mA plays an important role in growth and lipid metabolism of *N. oceanica*. However, the 6mA level increased only slightly (by 21.3%) in NO06G02500-knockout mutants, likely owing to the relatively short cell division cycle of microalgae (compared with higher plants). Moreover, ~65% of the DEGs still have no functional annotations (Supplemental File 1), underscoring the challenge in untangling the mechanisms behind the mutant phenotypes. The mechanistic links between 6mA and transcriptional regulation of lipid/growth-related genes, which to date derive mainly from multi-omics associations, should be investigated further by additional biochemical or genetic experiments.

Epigenetic manipulation of algal genomes holds untapped potential for increasing biofuel productivity (Steadman et al., 2020). Epigenetic proteins are druggable targets that can be addressed through small-molecule inhibitors. For example, in the microalga *Picochlorum soloecismus*, inhibition of DNA methylation using 5-aza-20-deoxycytidine increases cell size and lipid accumulation (Steadman et al., 2020). Moreover, the epigenome can be manipulated in a locus-specific manner via CRISPR–dCas9/12a (Sgro and Blancafort, 2020). Therefore, the genome-wide 6mA map and identification of key 6mA machineries reported here for the model oleaginous microalga *N. oceanica* pave the way for epigenetic engineering of superior algal feedstocks that can directly convert carbon dioxide to oils.

METHODS

Cultivation and SMRT sequencing of WT *N. oceanica* for construction of a genome-wide 6mA map

N. oceanica IMET1 was inoculated into modified f/2 liquid medium, which was prepared with 35 g L^{-1} sea salt, 1 g L^{-1} $NaNO_3$, 67 mg L^{-1} $NaH_2PO_4 \cdot H_2O$, 3.65 mg L^{-1} $FeCl_3 \cdot 6H_2O$, 4.37 mg L^{-1} $Na_2EDTA \cdot 2H_2O$, trace metal mix (0.0196 mg L^{-1} $CuSO_4 \cdot 5H_2O$, 0.0126 mg L^{-1} $NaMoO_4 \cdot 2H_2O$, 0.044 mg L^{-1} $ZnSO_4 \cdot 7H_2O$, 0.01 mg L^{-1} $CoCl_2$, and 0.36 mg L^{-1} $MnCl_2 \cdot 4H_2O$), and vitamin mix (2.5 μg L^{-1} vitamin B_{12}, 2.5 μg L^{-1} biotin, and 0.5 μg L^{-1} thiamine HCl). The algal cells were grown in liquid cultures under continuous light (~50 μmol photons m^{-2} s^{-1}) at 25°C and aerated by bubbling with a mixture of 1.5% CO_2 in air.

Cells were collected by centrifugation and used for genomic DNA isolation. Cultures were pelleted, and nucleic acids were extracted using phenol–chloroform and treated with RNase to degrade RNA. gDNA (5–10 μg) was sheared to >10 kb using g-TUBEs (Covaris). The sheared DNA was treated with DNA damage repair mix, followed by end repair and ligation of SMRT hairpin adapters using the SMRTbell Template Preparation Reagent Kit (Pacific Biosciences). Fragments without adaptors were digested with exonuclease. Libraries were sequenced on a Pacific Biosciences RS-II sequencer using standard protocols at the DOE Joint Genome Institute.

The PacBio SMRT analysis platform (version: 2.3.0.140936.p4) was used to detect DNA 6mA modifications. In brief, the subreads were filtered based on strict criteria (minimum read length of 100, read score of 0.8); the filtered reads were then aligned to the reference genome using pbalign (version: 0.2.0) with strict parameters ("–minAccuracy=0.75 –minLength=100 –concordant"); finally, kinetic analysis of the aligned subreads was used to identify DNA 6mA modifications with kineticsTools (Clark et al., 2012) in the "P_ModificationDetection" module. DNA 6mA modifications were extracted from the identified base modifications.

Computational mapping of the genome-wide distribution of DNA 6mA sites

DREME (from MEME suite v5.0.2) (Bailey, 2011) was used to find relatively enriched motifs in the two flanking 4-bp sequences of 6mA sites (9mers), with all candidate sequences (both strands) in the genome as controls and the parameters "-norc -mink 2 -maxk 5". Densely methylated adenine clusters were defined as at least 10 adjacent 6mA sites (with intervals <100 bp).

We defined the genome-wide N6-methylation level of adenine sites as the mean of 6mA sites from all adenine sites (strand specifically if not specified). The ratio of methylation for each adenine site cannot be reliably determined and was thus not taken into consideration. Analyses were strand specific unless otherwise indicated (several non-strand-specific analyses were also performed, with results shown in the Supplemental information). The percentage of methylation sites in each gene category was defined as above after considering the non-strand-specificity of intergenic regions. A fragment/gene was designated as methylated if any adenine it contained was N6-methylated. DNA 6mA distribution around the gene body, TSS, and TTS was calculated along the forward strand of the gene and drawn after smoothing (locally weighted scatterplot smoothing regression with f = 0.05). For a fair comparison, distribution of the 6mA ratio along the gene body (or intron) was consolidated into 1000 bp (or 200 bp) by uniform sampling or interpolation.

GO enrichment of *N. oceanica* genes with 6mA modifications was analyzed using the R package clusterProfiler (Wu et al., 2021) with a Q-value cutoff of 0.01. For cross-organism comparison, public datasets were retrieved from the Gene Expression Omnibus (GEO) database (*Chlamydomonas reinhardtii*, GSE 68860; *Arabidopsis thaliana*, GSM 2157793; *Oryza sativa*, GSE 103145).

Measurement of 6mA/A ratio by LC–MS/MS

N. oceanica 6mA and dA were profiled via LC–MS/MS (Allwegene Technologies Inc.). The extracted *N. oceanica* genomic DNA was first digested by the Dpn I restriction enzyme, then subjected to ultrafiltration to remove possible bacterial DNA contamination. DNA (dissolved in water) was first denatured by heating at 95°C for 5 min and then chilling on ice for 2 min. After addition of S1 nuclease buffer, alkaline phosphatase, and DNase I (Takara Biotechnology), the mixture was incubated at 37°C. After the DNA was completely digested into nucleosides, the mixture was extracted with chloroform. The resulting aqueous layer was collected, reconstituted in water, and analyzed by LC–electrospray ionization–MS/MS.

Dot blotting for DNA 6mA

Dot blotting was performed as described previously with minor modifications (Zhang et al., 2015). In brief, the synthesized oligos (Wu et al., 2016) were loaded on an HATF00010 nitrocellulose membrane (Merck Millipore) and air dried for 5 min. The membrane was baked at 80°C for 2 h and then blocked in blocking buffer (5% milk in PBST) for 2 h at room temperature. The membrane was incubated with a specific anti-6mA antibody (Synaptic systems; 1:2000) overnight at 4°C, then incubated with horseradish peroxidase-conjugated anti-rabbit IgG secondary antibody M21003 (Abmart; 1:2000) at room temperature for 1.5 h. The antibody-bound 6mA was then incubated with a high-sensitivity enhanced chemiluminescence reagent (Sangon Biotech) and detected and quantified with a FUSION Solo 6S imaging system (VILBER).

6mA methylation assays *in vitro*

In vitro methylation reactions were performed as described previously with minor modifications (Xiao et al., 2018). In brief, the reactions were

performed in a 25-μL methylation reaction buffer containing 250 pmol DNA NC-oligos, 800 ng recombinant GST-NO08G00280 protein, 50 mM Tris–HCl (pH 7.6), 50 mM KCl, 10 mM $Mg(OAc)_2$, 7 mM β-mercaptoethanol, 800 mM S-adenosylmethionine, and 100 μg/mL bovine serum albumin. Reactions were carried out overnight at 25°C. DNA was purified with a DP214 DNA purification kit (Tiangen Biotech), and the purified DNA was used for 6mA dot blotting. The NC-oligos used are listed in Supplemental Table 8.

6mA demethylation assays *in vitro*

In vitro demethylation reactions were performed as described previously with minor modifications (Wu et al., 2016). In brief, the reactions were performed in a 50-mL demethylation reaction buffer containing 50 pmol 6mA-oligos, 230 ng recombinant GST-NO06G02500 protein, 50 mM HEPES (pH 7.0), 50 mM KCl, 1 mM $MgCl_2$, 2 mM ascorbic acid, 1 mM a-KG, and 1 mM $(NH_4)_2Fe(SO_4)_2 \cdot 6H_2O$. Reactions were carried out for 1 h at 37°C, and 2 mL of reaction product was used for dot blotting. The 6mA-oligos used are listed in Supplemental Table 8.

CRISPR-based knockout of NO08G00280 and NO06G02500

For CRISPR-based targeted knockout in *N. oceanica* (Wang et al., 2016), a modular CRISPR–Cas9 toolbox system (pNOC-ARS-CRISPR) was used to construct the CRISPR plasmids (Poliner et al., 2018). Specifically, guide RNAs (gRNAs) were designed using the CHOPCHOP platform (http://chopchop.cbu.uib.no). For each gRNA, a pair of synthesized oligonucleotides named Target-3230-gRNA F and R were annealed to form a dimer with overhangs at both ends. Each dimer was ligated with the BspQI-digested plasmid pNOC-ARS-CRISPR to generate an entry clone with the full-length gRNA. The linearized vectors (containing a bleomycin resistance gene) with *AseI* digestion were introduced into the microalgae by electroporation (Li et al., 2014). For transformant selection, electroporated cells were plated onto 50% fresh seawater agar plates (1% agar) supplemented with 5 μg mL^{-1} zeocin (Invitrogen). After 2–3 weeks of incubation in white light (ca. 100 μmol m^{-2} s^{-1}) at 20°C, individual resistant colonies were inoculated into liquid f/2 medium with 5 μg mL^{-1} zeocin. The transformants were screened by checking for integration of the ShBle gene with the primers ble_fw and ble_rv.

Comparing the global 6mA maps of *N. oceanica* WT and mutants by SMRT sequencing

For both WT and mutants, microalgal cells were cultivated in a glass column (25 cm height × 3.5 cm diameter) under high light (150 ± 20 μmol photons m^{-2} s^{-1}) with an initial $OD_{750} = 0.5$ and bubbling with air. The samples were collected at 7 days for preparation of DNA and RNA libraries. *N. oceanica* cells were harvested by centrifugation for 5 min at 2500 × *g*, then immediately quenched with liquid N_2 and stored in a –80°C freezer.

DNA libraries for SMRT sequencing were constructed with an insert size of 20 kb using the SMRTbell Express Template Prep Kit 2.0, then sequenced on the PacBio Sequel II platform (Sequel II Sequencing Kit 2.0; running in CLR mode) at Novogene Biotech Co., Ltd.

PacBio SMRT Link (v10.1.0) was used for the resequencing and base modification analysis. The resequencing module was used to map subreads to the reference genome (with minimum mapped length set to 200 bp) and check variant information at the mutation sites. Then, datasets with sufficient sequencing depth, including two replicates of WT (WT_2 and WT_3), one replicate of M1 (M1_3), two replicates of M2 (M2_1 and M2_2), three replicates of M3 (M3_1, M3_2, and M3_3), and two replicates of M4 (M4_1 and M4_2), were subsampled to 100× (with a genome size of 31 Mb) from the mappable subreads. To detect DNA 6mA modification, the Base Modification Analysis module was used with a minimum mapped length of 200 bp and a minimum methylated fraction of 0.1. Finally, DNA 6mA modifications were extracted from the identified base modifications.

RNA-seq library construction for WT and mutant lines

Total algal RNA was extracted using TRIzol reagent (Tiangen). The concentration and purity of the RNA were determined spectrophotometrically (IMPLEN), and RNA integrity was assessed using the RNA Nano 6000 Assay Kit with the Agilent Bioanalyzer 2100. A total of 2 μg RNA per sample was used as input material for RNA sample preparation. Sequencing libraries were generated using the NEBNext Ultra RNA Library Prep Kit for Illumina (New England Biolabs) following the manufacturer's recommendations, and index codes were added to attribute sequences to each sample. Clustering of the index-coded samples was performed on a cBot Cluster Generation System using the HiSeq 3000/4000 PE Cluster Kit Box1 from Illumina. After cluster generation, the libraries were sequenced on the Illumina HiSeq 4000 platform, and 150-bp paired-end reads were generated.

Computational analysis of RNA-seq data

RNA-seq datasets were processed using the nfcore/rnaseq pipeline (https://nf-co.re/rnaseq), and reads were aligned using STAR with modified parameters to limit intron lengths (–alignIntronMin 20 –alignIntronMax 3000). Trinity (Grabherr et al., 2011) was then used to generate a gene expression matrix with the RSEM2 method and TMM normalization to account for variation in library size between samples. TPM values (Li and Dewey, 2011; Wagner et al., 2012) were averaged among replicates. For mutants, differentially expressed genes were identified using edgeR (Robinson et al., 2010) with an FDR of ≤0.001 and a minimum fold change of >2. Differences in expression between genes with and without 6mA modifications were determined on the basis of TPM values. A Wilcoxon test was performed to assess the statistical significance of TPMs in different categories.

Phenotyping of WT and mutant lines

For strain phenotyping, 10 mL of cells were collected by centrifugation after 7 days of culture and dried by vacuum freezing for at least 15 h before dry weight measurement. The lyophilized algal powder (5–10 mg) was stirred with 2 mL chloroform:methanol (2:1, v/v) for 1 h at 30°C. The extract was mixed with 1 mL 0.7% KCl and centrifuged at 1500 × *g* for 10 min; the lower organic layer was transferred to a new vial. The organic layer was evaporated under nitrogen gas and dried in a vacuum drying oven at 65°C for 1 h before calculation of net lipid content. Transmethylation was performed by incubating the extracted lipids with 20 μL 2 mg/mL tridecanoate, 200 μL chloroform:methanol (2:1, v/v), and 300 μL 5% HCL:methanol (v/v) at 85°C for 1 h. The fatty acid methyl esters (FAMEs) were extracted with hexane and analyzed directly by GC–MS using the Agilent 7890A GC system with an Agilent 19091-N133 column. Mixed analytical standards of FAMEs and pentadecane were used as external and internal standards, respectively. The amounts of the FA profiles were calculated on the basis of results derived from GC–MS. The chemicals used as standards were from Sigma.

TAGs were separated on a silica thin layer chromatography plate using a mixture of solvents consisting of petroleum ether, ethyl ether, and acetic acid (70:30:1 by volume), and TAG bands were scraped from the plate. FAMEs were prepared by acid-catalyzed transmethylation of the TAG bands and then analyzed by GC–MS as described previously. TAG amounts were calculated on the basis of results derived from GC–MS.

DATA AND CODE AVAILABILITY

For PacBio sequencing, raw sequencing data from the 19 runs have been deposited into the Sequence Read Archive (SRA) by the DOE Joint Genome Institute as SRR2022894 to SRR2022912. For Hi-C sequencing data, raw reads have been deposited into the SRA under accession SRR8420587. For MinION sequencing, raw signal files are available from the SRA under accession SRR8417804. The genome assembly,

annotation, and methylation analysis results can be downloaded from the NanDeSyn database (https://nandesyn.single-cell.cn/download; Gong et al., 2020). RNA-seq and 6mA profiling data from the mutants have been deposited in the GEO database with accession numbers GSE178672 and GSE212585. Accession numbers for the sequence data used in this study are provided in Supplemental Table 9. Raw sequence data reported in this paper have also been deposited in the Genome Sequence Archive at the National Genomics Data Center, China National Center for Bioinformation, under accession number PRJCA021066.

SUPPLEMENTAL INFORMATION

Supplemental information is available at *Plant Communications Online*.

FUNDING

This work was supported by the Synthetic Biology Program of the Ministry of Science and Technology of the People's Republic of China (2021YFA0909700), I201908 and E1551402 from the Qingdao Institute of Bioenergy and Bioprocess Technology, Chinese Academy of Sciences, 31900071 from the Natural Science Foundation of China, and ZR2019QC012 from the Natural Science Foundation of Shandong Province. We thank Professor Byeong-ryool Jeong for discussions.

AUTHOR CONTRIBUTIONS

J.X., Y.G., and L.W. conceived and designed the study; Q.W. and L.W. performed the experiments; Y.G. analyzed the sequencing data; W.L., L.W., N.L., X.D., J.Z., C.S., and Y.X. contributed to experiments and data interpretation; L.S. advised on manuscript writing; Y.G. and J.X. wrote the paper.

ACKNOWLEDGMENTS

No conflict of interest declared.

Received: December 28, 2022
Revised: February 9, 2023
Accepted: November 23, 2023
Published: November 24, 2023

REFERENCES

Alboresi, A., Perin, G., Vitulo, N., Diretto, G., Block, M., Jouhet, J., Meneghesso, A., Valle, G., Giuliano, G., Maréchal, E., et al. (2016). Light remodels lipid biosynthesis in *Nannochloropsis gaditana* by modulating carbon partitioning between organelles. Plant Physiol. **171**:2468–2482.

Babinger, P., Kobl, I., Mages, W., and Schmitt, R. (2001). A link between DNA methylation and epigenetic silencing in transgenic *Volvox carteri*. Nucleic Acids Res. **29**:1261–1271.

Bailey, T.L. (2011). DREME: motif discovery in transcription factor ChIP-seq data. Bioinformatics **27**:1653–1659.

Chen, M., Gao, X., Yang, W., Sun, C., Yang, J., Zhang, H., and Song, Y. (2020). Discovery and characterization of a stable lipase with preference toward long-chain fatty acids. Biotechnol. Lett. **42**:171–180.

Clark, T.A., Murray, I.A., Morgan, R.D., Kislyuk, A.O., Spittle, K.E., Boitano, M., Fomenkov, A., Roberts, R.J., and Korlach, J. (2012). Characterization of DNA methyltransferase specificities using single-molecule, real-time DNA sequencing. Nucleic Acids Res. **40**:e29.

Deniz, Ö., Frost, J.M., and Branco, M.R. (2019). Regulation of transposable elements by DNA modifications. Nat. Rev. Genet. **20**:417–431.

Dong, H.P., Williams, E., Wang, D.Z., Xie, Z.X., Hsia, R.C., Jenck, A., Halden, R., Li, J., Chen, F., and Place, A.R. (2013). Responses of *Nannochloropsis oceanica* IMET1 to long-term nitrogen starvation and recovery. Plant Physiol. **162**:1110–1126.

Dong, Y., Silbermann, M., Speiser, A., Forieri, I., Linster, E., Poschet, G., Allboje Samami, A., Wanatabe, M., Sticht, C., Teleman, A.A., et al. (2017). Sulfur availability regulates plant growth via glucose-TOR signaling. Nat. Commun. **8**:1174.

Fan, X., Han, W., Teng, L., Jiang, P., Zhang, X., Xu, D., Li, C., Pellegrini, M., Wu, C., Wang, Y., et al. (2020). Single-base methylome profiling of the giant kelp *Saccharina japonica* reveals significant differences in DNA methylation to microalgae and plants. New Phytol. **225**:234–249.

Fu, Y., Luo, G.-Z., Chen, K., Deng, X., Yu, M., Han, D., Hao, Z., Liu, J., Lu, X., Doré, L.C., et al. (2015). N^6-methyldeoxyadenosine marks active transcription start sites in *chlamydomonas*. Cell **161**:879–892.

Gong, Y., Kang, N.K., Kim, Y.U., Wang, Z., Wei, L., Xin, Y., Shen, C., Wang, Q., You, W., Lim, J.-M., et al. (2020). The NanDeSyn database for *Nannochloropsis* systems and synthetic biology. Plant J. **104**:1736–1745.

Grabherr, M.G., Haas, B.J., Yassour, M., Levin, J.Z., Thompson, D.A., Amit, I., Adiconis, X., Fan, L., Raychowdhury, R., Zeng, Q., et al. (2011). Full-length transcriptome assembly from RNA-Seq data without a reference genome. Nat. Biotechnol. **29**:644–652.

Greer, E.L., Blanco, M.A., Gu, L., Sendinc, E., Liu, J., Aristizábal-Corrales, D., Hsu, C.H., Aravind, L., He, C., and Shi, Y. (2015). DNA methylation on N^6-adenine in *C. elegans*. Cell **161**:868–878.

Han, X., Song, X., Li, F., and Lu, Y. (2020). Improving lipid productivity by engineering a control-knob gene in the oleaginous microalga *Nannochloropsis oceanica*. Metab. Eng. Commun. **11**, e00142.

Hattman, S., Kenny, C., Berger, L., and Pratt, K. (1978). Comparative study of DNA methylation in three unicellular eucaryotes. J. Bacteriol. **135**:1156–1157.

Huete-Ortega, M., Okurowska, K., Kapoore, R.V., Johnson, M.P., Gilmour, D.J., and Vaidyanathan, S. (2018). Effect of ammonium and high light intensity on the accumulation of lipids in *Nannochloropsis oceanica* (CCAP 849/10) and *Phaeodactylum tricornutum* (CCAP 1055/1). Biotechnol. Biofuels **11**:60.

Janssen, J.H., Spoelder, J., Koehorst, J.J., Schaap, P.J., Wijffels, R.H., and Barbosa, M.J. (2020). Time-dependent transcriptome profile of genes involved in triacylglycerol (TAG) and polyunsaturated fatty acid synthesis in *Nannochloropsis gaditana* during nitrogen starvation. J. Appl. Phycol. **32**:1153–1164.

Jithu Paul, J. (2020). Bioconcentration of Marine Algae Using Lipase Enzyme (IntechOpen).

Julio, S.M., Heithoff, D.M., Provenzano, D., Klose, K.E., Sinsheimer, R.L., Low, D.A., and Mahan, M.J. (2001). DNA adenine methylase is essential for viability and plays a role in the pathogenesis of *Yersinia pseudotuberculosis* and *Vibrio cholerae*. Infect. Immun. **69**:7610–7615.

Kahng, L.S., and Shapiro, L. (2001). The CcrM DNA methyltransferase of *Agrobacterium tumefaciens* is essential, and its activity is cell cycle regulated. J. Bacteriol. **183**:3065–3075.

Li, B., and Dewey, C.N. (2011). RSEM: accurate transcript quantification from RNA-Seq data with or without a reference genome. BMC Bioinf. **12**:323.

Li, J., Han, D., Wang, D., Ning, K., Jia, J., Wei, L., Jing, X., Huang, S., Chen, J., Li, Y., et al. (2014). Choreography of transcriptomes and lipidomes of *Nannochloropsis* reveals the mechanisms of oil synthesis in microalgae. Plant Cell **26**:1645–1665.

Li, Y., Zhang, X.-M., Luan, M.-W., Xing, J.-F., Chen, J., and Xie, S.-Q. (2020). Distribution patterns of DNA N6-methyladenosine modification in non-coding RNA genes. Front. Genet. **11**:268.

Liang, Z., Geng, Y., and Gu, X. (2018a). Adenine methylation: new epigenetic marker of DNA and mRNA. Mol. Plant **11**:1219–1221.

Liang, Z., Yu, G., Liu, J., Geng, Y., Mao, J., Wang, D., Zhou, J., and Gu, X. (2018b). The N6-adenine methylation in yeast genome profiled by single-molecule technology. Journal of Genetics and Genomics **45**:223–225.

Liu, F., Clark, W., Luo, G., Wang, X., Fu, Y., Wei, J., Wang, X., Hao, Z., Dai, Q., Zheng, G., et al. (2016). ALKBH1-mediated tRNA demethylation regulates translation. Cell **167**:816–828.e16.

Long, K., Ghazali, H.M., Ariff, A., Man, Y.C., and Bucke, C. (1998). Substrate preference of mycelium-bound lipase from a strain of *Aspergillus Flavus* Link. Biotechnol. Lett. **20**:369–372.

Ma, C., Niu, R., Huang, T., Shao, L.-W., Peng, Y., Ding, W., Wang, Y., Jia, G., He, C., Li, C.-Y., et al. (2018). N6-methyldeoxyadenine is a transgenerational epigenetic signal for mitochondrial stress adaptation. Nat. Cell Biol. **21**:319–327.

Ma, X.-N., Chen, T.-P., Yang, B., Liu, J., and Chen, F. (2016). Lipid production from *Nannochloropsis*. Mar. Drugs **14**:61.

Merchant, S.S., and Helmann, J.D. (2012). Chapter 2 - Elemental economy: microbial strategies for optimizing growth in the face of nutrient limitation. In Advances in Microbial Physiology, R.K. Poole, ed. (Academic Press), pp. 91–210.

Mondo, S.J., Dannebaum, R.O., Kuo, R.C., Louie, K.B., Bewick, A.J., Labutti, K., Haridas, S., Kuo, A., Salamov, A., Ahrendt, S.R., et al. (2017). Widespread adenine N6-methylation of active genes in fungi. Nat. Genet. **49**:964–968.

Poliner, E., Takeuchi, T., Du, Z.-Y., Benning, C., and Farré, E.M. (2018). Nontransgenic marker-free gene disruption by an episomal CRISPR system in the oleaginous microalga, *Nannochloropsis oceanica* CCMP1779. ACS Synth. Biol. **7**:962–968.

Reisenauer, A., and Shapiro, L. (2002). DNA methylation affects the cell cycle transcription of the CtrA global regulator in *Caulobacter*. EMBO J. **21**:4969–4977.

Robinson, M.D., Mccarthy, D.J., and Smyth, G.K. (2010). edgeR: a Bioconductor package for differential expression analysis of digital gene expression data. Bioinformatics **26**:139–140.

Sgro, A., and Blancafort, P. (2020). Epigenome engineering: new technologies for precision medicine. Nucleic Acids Res. **48**:12453–12482.

Singh, J., and Saxena, R.C. (2015). Chapter 2 - An introduction to microalgae: diversity and significance. In Handbook of Marine Microalgae, S.-K. Kim, ed. (Academic Press), pp. 11–24.

Steadman, C.R., Banerjee, S., Kunde, Y.A., Sanders, C.K., Marrone, B.L., and Twary, S.N. (2020). Inhibition of DNA methylation in *Picochlorum soloecismus* alters algae productivity. Front. Genet. **11**, 560444.

Takahashi, F., Yamagata, D., Ishikawa, M., Fukamatsu, Y., Ogura, Y., Kasahara, M., Kiyosue, T., Kikuyama, M., Wada, M., and Kataoka, H. (2007). AUREOCHROME, a photoreceptor required for photomorphogenesis in stramenopiles. Proc. Natl. Acad. Sci. USA **104**:19625–19630.

Tarrago, L., Laugier, E., and Rey, P. (2009). Protein-repairing methionine sulfoxide reductases in photosynthetic organisms: gene organization, reduction mechanisms, and physiological roles. Mol. Plant **2**:202–217.

Tejada-Jimenez, M., Chamizo-Ampudia, A., Calatrava, V., Galvan, A., Fernandez, E., and Llamas, A. (2018). From the eukaryotic molybdenum cofactor biosynthesis to the moonlighting enzyme mARC. Molecules **23**:3287.

Vieler, A., Brubaker, S.B., Vick, B., and Benning, C. (2012). A lipid droplet protein of *Nannochloropsis* with functions partially analogous to plant oleosins. Plant Physiol. **158**:1562–1569.

Wagner, G.P., Kin, K., and Lynch, V.J. (2012). Measurement of mRNA abundance using RNA-seq data: RPKM measure is inconsistent among samples. Theor. Biosci. **131**:281–285.

Wang, Q., Lu, Y., Xin, Y., Wei, L., Huang, S., and Xu, J. (2016). Genome editing of model oleaginous microalgae *Nannochloropsis* spp. by CRISPR/Cas9. Plant J. **88**:1071–1081.

Wang, Y., Chen, X., Sheng, Y., Liu, Y., and Gao, S. (2017). N6-adenine DNA methylation is associated with the linker DNA of H2A.Z-containing well-positioned nucleosomes in Pol II-transcribed genes in *Tetrahymena*. Nucleic Acids Res. **45**:11594–11606.

Wang, Y., Sheng, Y., Liu, Y., Zhang, W., Cheng, T., Duan, L., Pan, B., Qiao, Y., Liu, Y., and Gao, S. (2019). A distinct class of eukaryotic MT-A70 methyltransferases maintain symmetric DNA N6-adenine methylation at the ApT dinucleotides as an epigenetic mark associated with transcription. Nucleic Acids Res. **47**:11771–11789.

Wei, B., Liu, H., Liu, X., Xiao, Q., Wang, Y., Zhang, J., Hu, Y., Liu, Y., Yu, G., and Huang, Y. (2016). Genome-wide characterization of non-reference transposons in crops suggests non-random insertion. BMC Genom. **17**:536.

Wei, L., El Hajjami, M., Shen, C., You, W., Lu, Y., Li, J., Jing, X., Hu, Q., Zhou, W., Poetsch, A., and Xu, J. (2019). Transcriptomic and proteomic responses to very low CO_2 suggest multiple carbon concentrating mechanisms in *Nannochloropsis oceanica*. Biotechnol. Biofuels **12**:168.

Wright, R., Stephens, C., and Shapiro, L. (1997). The CcrM DNA methyltransferase is widespread in the alpha subdivision of proteobacteria, and its essential functions are conserved in *Rhizobium meliloti* and *Caulobacter crescentus*. J. Bacteriol. **179**:5869–5877.

Wu, T., Hu, E., Xu, S., Chen, M., Guo, P., Dai, Z., Feng, T., Zhou, L., Tang, W., Zhan, L., et al. (2021). clusterProfiler 4.0: a universal enrichment tool for interpreting omics data. Innovation **2**, 100141.

Wu, T.P., Wang, T., Seetin, M.G., Lai, Y., Zhu, S., Lin, K., Liu, Y., Byrum, S.D., Mackintosh, S.G., Zhong, M., et al. (2016). DNA methylation on N(6)-adenine in mammalian embryonic stem cells. Nature **532**:329–333.

Xiao, C.-L., Zhu, S., He, M., Chen, D., Zhang, Q., Chen, Y., Yu, G., Liu, J., Xie, S.-Q., Luo, F., et al. (2018). N^6-methyladenine DNA modification in the human genome. Mol. Cell **71**:306–318.e7.

Xin, Y., Lu, Y., Lee, Y.-Y., Wei, L., Jia, J., Wang, Q., Wang, D., Bai, F., Hu, H., Hu, Q., et al. (2017). Producing designer oils in industrial microalgae by rational modulation of co-evolving type-2 diacylglycerol acyltransferases. Mol. Plant **10**:1523–1539.

Xin, Y., Shen, C., She, Y., Chen, H., Wang, C., Wei, L., Yoon, K., Han, D., Hu, Q., and Xu, J. (2019). Biosynthesis of triacylglycerol molecules with a tailored PUFA profile in industrial microalgae. Mol. Plant **12**:474–488.

Yao, B., Cheng, Y., Wang, Z., Li, Y., Chen, L., Huang, L., Zhang, W., Chen, D., Wu, H., Tang, B., et al. (2017). DNA N6-methyladenine is dynamically regulated in the mouse brain following environmental stress. Nat. Commun. **8**:1122.

Zhang, G., Huang, H., Liu, D., Cheng, Y., Liu, X., Zhang, W., Yin, R., Zhang, D., Zhang, P., Liu, J., et al. (2015). N6-methyladenine DNA modification in *Drosophila*. Cell **161**:893–906.

Zhang, M., Yang, S., Nelakanti, R., Zhao, W., Liu, G., Li, Z., Liu, X., Wu, T., Xiao, A., and Li, H. (2020). Mammalian ALKBH1 serves as an N6-mA demethylase of unpairing DNA. Cell Res. **30**:197–210.

Zhang, P., Xin, Y., He, Y., Tang, X., Shen, C., Wang, Q., Lv, N., Li, Y., Hu, Q., and Xu, J. (2022). Exploring a blue-light-sensing transcription factor to double the peak productivity of oil in *Nannochloropsis oceanica*. Nat. Commun. **13**:1664.

Zhang, Q., Liang, Z., Cui, X., Ji, C., Li, Y., Zhang, P., Liu, J., Riaz, A., Yao, P., Liu, M., et al. (2018). N6-methyladenine DNA methylation in *Japonica* and *Indica* rice genomes and its association with gene expression, plant development, and stress responses. Mol. Plant **11**:1492–1508.

Liang, Z., Shen, L., Cui, X., Bao, S., Geng, Y., Yu, G., Liang, F., Xie, S., Lu, T., Gu, X., et al. (2018). DNA N6-adenine methylation in *Arabidopsis thaliana*. Dev. Cell **45**:406–416.e3.

Zhou, C., Wang, C., Liu, H., Zhou, Q., Liu, Q., Guo, Y., Peng, T., Song, J., Zhang, J., Chen, L., et al. (2018). Identification and analysis of adenine N6-methylation sites in the rice genome. Nat. Plants **4**:554–563.

Zienkiewicz, A., Zienkiewicz, K., Poliner, E., Pulman, J.A., Du, Z.-Y., Stefano, G., Tsai, C.-H., Horn, P., Feussner, I., Farre, E.M., et al. (2020). The microalga *Nannochloropsis* during transition from quiescence to autotrophy in response to nitrogen availability. Plant Physiol. **182**:819–839.

Plant Communications, Volume *5*

Supplemental information

Genome-wide adenine N6-methylation map reveals epigenomic regulation of lipid accumulation in *Nannochloropsis*

Yanhai Gong, Qintao Wang, Li Wei, Wensi Liang, Lianhong Wang, Nana Lv, Xuefeng Du, Jiashun Zhang, Chen Shen, Yi Xin, Luyang Sun, and Jian Xu

Supplemental Results

Nannochloropsis oceanica genome assembly using the PacBio and Hi-C data

The most high quality nuclear genome of *N. oceanica* IMET1 published up to date (Wang et al., 2014) is fragmented (293 scaffolds) and with significant amount of ambiguous bases (11%) which will lead to low accuracy of 6mA detection. The lack of a high-quality reference genome impedes investigations of genome-wide 6mA distribution patterns and other downstream studies. Therefore, we started by *de novo* assembly for an improved nuclear genome using ultra-high depth of PacBio and Hi-C data. We generated ~340X sequencing coverage of the *N. oceanica* IMET1 genome using 19 SMRT cells on the PacBio RS-II platform (**Methods**; **Table S1**). The resulting subreads had a read N50 length of over 9.43 Kb (**Table S1**). The longest ~120X subreads were self-corrected using all the subreads, then the longest ~50X error-corrected reads were assembled using Celera assembler (PBcR; (Berlin et al., 2015)) followed by polishing via Quiver (**Supplemental Methods**). The assembly contained 122 contigs spanning 31.54 Mb genome size with N50 length 907.6 Kb and without any gaps (ambiguous bases). Then, a repeat-aware de novo assembly upgrading tool -- FinisherSC (Lam et al., 2015) was used to resolve repeated genome structures and improve contiguity followed by another round of polishing via Quiver (**Supplemental Methods**). Finally, putative contaminations were removed. Above assembly strategy helped us to obtain 70 contigs with total length of 31.0 Mb (with N50 length of 1.08 Mb; **Table S2**). We also found 11 contigs with telomeric repeats (TTAGGG; (Corteggiani Carpinelli et al., 2014)) at both extremities and 33 contigs with only one telomere.

To further improve the continuity of nuclear genome and thoroughly resolve its chromosome architectures, we utilized a high-resolution contact map derived from ultra-high

depth of Hi-C data (**Supplemental Methods**). Hi-C interaction maps share common features which relate 3D interaction frequencies to the 1D ordering of the genome (Oddes et al., 2018), and have been used to improve draft genome assemblies to create chromosome-length scaffolds for large genomes (Bickhart et al., 2017; Dudchenko et al., 2017; Mascher et al., 2017). We obtained ultra-high (~2000X) coverage of *in situ* Hi-C data, and 319.6 million read pairs (92.16%) passed the quality filter to constitute the clean data. Data analysis showed 73.8% pairs were uniquely mapped and 72.2% were valid interaction pairs (55.6% after removing PCR duplicates). Initial scaffolding attempts using LACHESIS (Burton et al., 2013) and 3d-dna (Dudchenko et al., 2017) clustered all contigs into one chromosome. To maximize the number of ordered and oriented contigs, we manually scaffolded these contigs mainly based on the inter-contigs contact map (generated by LACHESIS). Two aberrant contigs were rejected with careful examination; then, the positions of telomeres were leveraged to resolve potential conflicts/ambiguities; in the end, the scaffoldings were rechecked by inspecting the contact map of scaffolded pseudo-chromosomes. Previous analyses of potential connections between contigs are consistent with the manual scaffolding based on Hi-C.

The final assembly (NoIMET1v2) were scaffolded into 30 pseudo-chromosomes (**Table S2**) which is consistent with the estimation for *N. gaditana* (Corteggiani Carpinelli et al., 2014; Schwartz et al., 2018) (**Fig. S1A**). The NoIMET1v2 assembly had 9.8-fold higher contiguity than NoIMET1v1, ~1,500 gaps eliminated/filled, and with average base accuracy at ~99.988% (**Fig. S1B** and **Table S2**). Besides, compared with data that were obtained using NoIMET1v1 (Wang et al., 2014) as reference, the 6mA map reveals ~12% more 6mA sites, while the average distance between adjacent 6mA sites is 9.2% lower (Wilcoxon test, $p < 0.001$; **Fig. S3**).

Genome-wide annotation of transposons and gene models

The improved genome of NoIMET1v2 enabled complete genome-wide annotation of transposons, which could potentially affect the function of DNA 6mA. For example, in rice, transposon associated genes (TEGs) with the highest expression displayed the lowest 6mA levels at the TSS, suggesting that 6mA at the TSS represses TEG expression (Zhou et al., 2018). In *Drosophila melanogaster*, 6mA sites are enriched in transposable elements and correlated with transposon expression (Zhang et al., 2015). So, we firstly identified repeat elements genome-wide through *de novo* repeat family identification, and annotated 16.2% of the assembly as repetitive element content (**Supplemental Methods**; **Fig. S1C**). Besides simple repeats (7.08%) and low complexity fragments (2.44%), DNA elements were the most abundant (3.97%), of which the DNA/CMC-EnSpm elements occupied 3.84% of the genome (**Fig. S1C**). En/Spm is the most thoroughly studied member of the CACTA TE superfamily having TIRs terminating with the sequence CACTA and the creation of 3-bp TSDs (Gbadegesin and Beeching, 2010). Apart from the main groups of DNA elements, 0.65% of the genome was annotated as long interspersed nuclear elements (LINE) and 0.12% as LTR elements, whereas the remainder was either assigned to other repeat families or could not be assigned (**Fig. S1B** and **Fig. S1C**). Among the several sequenced algae and plant species (**Table S3**), the repetitive elements identified in the *N. oceanica* genome were comparable to those in diatom (TEs contribute 6.4% of the *P. tricornutum* genome and 1.9% of the *T. pseudonana* genome, with LTR-RTs the most abundant in both genomes; (Maumus et al., 2009)). Class I elements (retrotransposons or retroelements) are the most widespread class of eukaryotic TEs (International Human Genome Sequencing et al., 2001; SanMiguel et al., 1996), but in *N. oceanica* DNA/CMC-EnSpm elements are the most

abundant TEs, which could serve as a valuable model to study the influence of DNA/CMC-EnSpm elements.

The initial set of *N. oceanica* IMET1 gene models for NoIMET1v1 genome was generated using EVidenceModeler (Haas et al., 2008) based on a limited amount of transcriptomic information (387K 454 cDNA reads; (Wang et al., 2014)) in nitrogen-repletion/starvation conditions (three time points; no replicates) and therefore involved a significant amount of *de novo* prediction. To provide diversity of transcripts, ~307 million pairs of reads, obtained from 54 RNA-Seq libraries (GEO datasets GSE42508 and GSE55861) among nitrogen-repletion/starvation conditions (six time points; three replicates) and CO_2-starvation conditions (five time points; two replicates) were used as evidence (**Supplemental Methods**). As a starting point, 10,353 protein-coding genes were predicted using BRAKER1 (Hoff et al., 2016) with hints from the alignments of these RNA-Seq data. These gene models were further improved by exploiting spliced alignments of transcript sequences using PASA2 (Haas et al., 2011) pipeline to annotate UTRs, add alternative splicing variants, merge/split genes, adjust boundaries and model novel genes (**Supplemental Methods**). Finally, 10,333 gene models (12,442 isoforms) were obtained, including 1,777 novel gene loci (**Table S4**). These protein-coding genes were further analyzed to predict biological functions by comparing protein sequences with the NR, InterPro and eggNOG databases (**Supplemental Methods**). This process allowed functional annotations to be assigned to 88.4%, 77.7% and 68.2% transcripts, respectively (**Table S4**).

Genome sequence validation using 454 and RNA-Seq data

Reads from 454 sequencing (Wang et al., 2014) were mapped to NoIMET1v1 and NoIMET1v2 respectively (**Supplemental Methods**). For NoIMET1v2, 1,834 high confidence

SNPs and 638 high confidence indels were found, which was less than half compared to NoIMET1v1 (**Fig. S1D**). RNA-Seq reads (from GSE42508 and GSE55861) were also individually mapped (**Supplemental Methods**) with 91.78% mapping ratio compared with 89.91% for NoIMET1v1. Similarly, the number of variants found was about 30% less (**Fig. S1D**).

Assessment of DNA contamination on 6mA detection based on simulated datasets

The abundance of DNA 6mA in eukaryotes may be overestimated due to prokaryotic DNA and RNA contamination in the original genomic DNA samples or in the enzymes for sample processing (Kong et al., 2022; O'Brown et al., 2019). To tackle this problem, firstly, during 6mA detection, we applied strict parameters for subread filtering and aligning (**Methods**), which greatly reduces false positives; then, based on multiple simulated datasets of PacBio sequencing data from NCBI BioProject PRJNA477598 (McIntyre et al., 2019), we assessed the degree to which the contaminated reads would interfere with the 6mA map of *N. oceanica*. We simulated the situation of DNA contamination by manually adding PacBio sequencing data from multiple microorganisms (yeast and seven bacteria) and generated nine mock datasets (**Supplemental Methods**). Detection of 6mA on these datasets (**Table S6**) revealed that: (*i*) very few such yeast and bacterial PacBio subreads (<1‰ in bases) would be aligned to the reference genome of *N. oceanica*; (*ii*) adding these contaminating DNA hardly changed the number and predicted loci of 6mA in the *N. oceanica* genome. Therefore, it is unlikely that the genome-wide 6mA map for *N. oceanica* is skewed by bacterial and fungal DNA contaminations.

Supplemental Methods

Hi-C library preparation and sequencing

Following the standard protocol described previously with certain modifications (Belton et al., 2012), we constructed Hi-C libraries using the *N. oceanica* cells as inputs (under low and high CO_2 conditions). Briefly, the microalgal cells were cross-linked by 2% formaldehyde solution at room temperature in a vacuum for 30 mins. 2.5 M glycine was added to quench the crosslinking reaction for 10 mins at room temperature. After ground with liquid nitrogen and re-suspended with 25 ml of extraction buffer I (0.4 M sucrose, 10 mM Tris-HCl, pH 8, 10 mM $MgCl_2$, 5 mM β-mercaptoethanol, 0.1 mM phenylmethylsulfonyl fluoride [PMSF], and 13 units protease inhibitor), the mixture supernatant was further centrifuged at 4000 rpm at 4 °C for 20 mins. Re-suspended pellet in extraction buffer II (0.25 M sucrose, 10 mM Tris-HCl, pH 8, 10 mM MgCl2, 1% Triton X-100, 5 mM β-mercaptoethanol, 0.1 mM PMSF, and 13 units protease inhibitor) was centrifuged at 14,000 rpm and 4 °C for 10 mins. The pellet was re-suspended in extraction buffer III (1.7 M sucrose, 10 mM Tris-HCl, pH 8, 0.15% Triton X-100, 2 mM MgCl2, 5mM β-mercaptoethanol, 0.1 mM PMSF, and 13 units protease inhibitor) and loaded on the top of an equal amount of clean extraction buffer III, which was then centrifuged at 14,000 rpm for 10 mins. The pellet was washed twice in 500 μL ice cold 1x CutSmart buffer and then centrifuged for 5 mins at 2,500 g. The nuclei were washed by 0.5 mL of restriction enzyme buffer and solubilized with dilute SDS followed by incubation at 65 °C for 10 min. After quenching the SDS by Triton X-100, an overnight digestion was applied to the samples with a 6-cutter restriction enzyme HindIII (400 units) at 37 °C on a rocking platform.

The subsequent steps involved marking the DNA ends with biotin-14-dCTP and blunt-end ligation of the cross-linked fragments. The proximal chromatin DNA was re-ligated by ligation enzyme. The nuclear complexes were revers cross-linked by incubation with the proteinase K at

65 °C. DNA was purified by the phenol-chloroform extraction. Biotin was removed from non-ligated fragment ends using T4 DNA polymerase. Ends of sheared fragments by sonication (200-600 base pairs) were repaired by the mixture of T4 DNA polymerase, T4 polynucleotide kinase and Klenow DNA polymerase. Biotin-labeled Hi-C samples were specifically enriched using streptavidin C1 magnetic beads. After adding A-tails to the fragment ends and following ligation by the illumina paired-end (PE) sequencing adapters, Hi-C sequencing libraries were amplified by PCR (10-15 cycles) and sequenced on Illumina HiSeq-2500 platform (PE 125bp).

Genome assembly based on the PacBio and Hi-C data for *N. oceanica*

PacBio reads were sequenced using 19 SMRT cells with P6-C4 chemistry on the PacBio RS-II platform (**Methods**). PacBio subreads were first *de novo* assembled using PBcR in Celera Assembler (Berlin et al., 2015)with parameters "-length 500 -partitions 200 -maxCoverage 120", then polished using Quiver tool from SMRT-Analysis package version 2.3.0. After that, FinisherSC (finishingTool-2.1; (Lam et al., 2015)) was used to resolve potential repeats and promote continuity. Finally, another round of Quiver was used to improve base qualities.

The PacBio only assembly was visualized by TAGC (Taxon-annotated Gc-Coverage) plot using Blobtools (Laetsch and Blaxter, 2017), for this, taxon annotations were generated from blasting to NCBI NT database using parameters "-culling_limit 5 -evalue 1e-25" and UniRef90 database using "diamond blastx" tool (Buchfink et al., 2015), coverages of every contig/scaffold were calculated from remapping PacBio subreads using pbalign (from SMRT-Analysis). Contigs/scaffolds without proper annotations (not alga) were manually examined and contaminated ones were removed from the assembly.

One high-resolution Hi-C library (high CO_2) was prepared and sequenced using an Illumina

HiSeq instrument with PE150 layout to yield approximately 2000X coverage of the *N. oceanica* IMET1 genome. Raw sequencing reads were filtered by removing adapter contaminated reads, low quality reads and reads with more than 5% ambiguous bases. Clean reads were mapped to the draft genome using Juicer v1.5.6 (Durand et al., 2016) and HiC-Pro v2.9.0 (Servant et al., 2015). LACHESIS (Burton et al., 2013) and 3d-dna (Dudchenko et al., 2017) were tested to scaffold PacBio assembled contigs, but both pipelines assembled all contigs into one chromosome which was unreasonable. To resolve potential conflicts, the inter-contigs contact map generated using LACHESIS (Burton et al., 2013) was used as global reference, then orders and orientations between contigs were manually examined with the assistance of Juicebox (Durand et al., 2016) for visualization of Hi-C contact matrix and revised with the assistance of external information such as position of telomeres in contigs, PacBio reads connection between contigs and synteny between other *Nannochloropsis* species. Synteny analysis between *N. oceanica* NoIMET1v1, *N. oceanica* NoIMET1v2, *N. oceanica* CCMP1779, *N. salina* CCMP1776, *N. gaditana* B-31 and *C. reinhardtii* v4.0 were performed using SyMAP ((Soderlund et al., 2011); with parameters to allow merging of synteny blocks).

Assessment of genome assembly NoIMET1v2

To evaluate the quality of NoIMET1v2, 454 clean reads (Wang et al., 2014) were mapped to the assembly using runMapping from 454 Sequencing System Off-Instrument Software Applications suite, SNP information were extracted from the output files. RNA-Seq reads from each library were mapped to the genome using HISAT2 ((Pertea et al., 2016); with parameters "--max-intronlen 2000 --dta -k 1 -X 1000 --no-mixed --no-discordant --mm"), variants were called using HaplotypeCaller from GATK ((Auwera et al., 2013); with parameters "-

dontUseSoftClippedBases -stand_call_conf 20.0 -stand_emit_conf 20.0") and filtered using VariantFiltration (with parameters: -window 35 -cluster 3 -filterName FS -filter "FS > 30.0" -filterName QD -filter "QD < 2.0").

Genome-wide annotation for NoIMET1v2

RNA-Seq data were used to assist the identification and structural annotation of genes. These data were previously sequenced and taken from GSE42508 (Li et al., 2014) and GSE55861 (Wei et al., 2019). All the raw reads were quality controlled using a script (from http://justpreprocessmyreads.sourceforge.net) with parameters "-cdna -qtrim 10 -slide_window 8 -slide_quality 20 -min_length 70". All the RNA-Seq clean reads were mapped to the genome using HISAT2 (Kim et al., 2015) (see above). A primitive reference annotation was made using BRAKER1 (Hoff et al., 2016) with evidence hints from all RNA-Seq mapping files. Then, the gene models were refined (annotating UTRs, creating alternatively spliced isoforms, etc) using PASA2 (Haas et al., 2003) served on the online platform GenSAS v5.1 (Lee et al., 2011) (https://www.gensas.org). During the refinement, the RNA-Seq data sets were in silico normalized to targeted maximum read coverage of 100 using Trinity (Grabherr et al., 2011).

The resultant protein isoforms were annotated via searching against the NCBI NR database using diamond blastp (diamond 0.8.36, evalue 1e-6, max_target_seqs 1) and predicting protein families and functional domains using InterProScan v5 (Jones et al., 2014) with parameters "--applications Pfam, PRINTS, ProDom, ProSiteProfiles, SMART, SUPERFAMILY, PANTHER -dp -iprlookup". Then, GO/KO/COG annotations were inferred based on orthology assignments from eggNOG database using eggNOG-Mapper (Huerta-Cepas et al., 2017).

Additionally, repeats were identified and masked using RepeatModeler and RepeatMasker

(http://www.repeatmasker.org), potential ncRNAs were inferred using Infernal cmscan (Nawrocki and Eddy, 2013) by searching the CM-format Rfam database with parameters "--rfam --nohmmonly --fmt 2 --cut_ga", tRNA and rRNA locus were extracted from the output and combined with the outputs from tRNAscan-SE ((Lowe and Eddy, 1997); Cove mode), barrnap (https://github.com/tseemann/barrnap; with parameters: "--kingdom euk") and RNAmmer ((Lagesen et al., 2007); with parameters: "-S euk -m lsu,ssu,tsu -multi") manually.

Detection of 6mA modifications for the datasets that simulated microbial contamination

To test the influence of DNA contamination on the calling of 6mA from our *N. oceanica* PacBio data, simulated datasets were used for comparison. These simulated datasets were synthesized by mixing *N. oceanica* PacBio reads with PacBio reads from different microbial sources (**Table S6**). Firstly, eight PacBio sequencing datasets (originated from *Bacillus subtilis*, *Enterococcus faecalis*, *Escherichia coli*, *Listeria monocytogenes*, *Pseudomonas aeruginosa*, *Saccharomyces cerevisiae*, *Salmonella enterica* and *Staphylococcus aureus*, respectively) were obtained from NCBI BioProject PRJNA477598 (McIntyre et al., 2019). Secondly, each dataset was combined with the PacBio sequencing data of *N. oceanica* to make eight simulated datasets, and a special simulated dataset ("Altogether", which represents the situation of heavy contamination) was generated by combining the eight collected datasets and *N. oceanica* data. Finally, the DNA 6mA sites for the nine simulated datasets were detected (**Methods**) and compared with the 6mA sites used in this study. Collectively, 99.8% of all 6mA sites were consistent between the two, even under the circumstance of heavy contamination (i.e., the simulated dataset of "Altogether").

References

Auwera, G.A., Carneiro, M.O., Hartl, C., Poplin, R., Angel, G.d., Levy‐Moonshine, A., Jordan, T., Shakir, K., Roazen, D., Thibault, J.*, et al.* (2013). From FastQ data to high‐confidence variant calls: the genome analysis toolkit best practices pipeline. Current Protocols in Bioinformatics *43*, 11.10.11-11.10.33.

Belton, J.-M., McCord, R.P., Gibcus, J.H., Naumova, N., Zhan, Y., and Dekker, J. (2012). Hi-C: a comprehensive technique to capture the conformation of genomes. Methods *58*, 268-276.

Berlin, K., Koren, S., Chin, C.-S., Drake, J.P., Landolin, J.M., and Phillippy, A.M. (2015). Assembling large genomes with single-molecule sequencing and locality-sensitive hashing. Nature Biotechnology *33*, 623.

Bickhart, D.M., Rosen, B.D., Koren, S., Sayre, B.L., Hastie, A.R., Chan, S., Lee, J., Lam, E.T., Liachko, I., Sullivan, S.T.*, et al.* (2017). Single-molecule sequencing and chromatin conformation capture enable de novo reference assembly of the domestic goat genome. Nature Genetics *49*, 643.

Buchfink, B., Xie, C., and Huson, D.H. (2015). Fast and sensitive protein alignment using DIAMOND. Nature Methods *12*, 59-60.

Burton, J.N., Adey, A., Patwardhan, R.P., Qiu, R., Kitzman, J.O., and Shendure, J. (2013). Chromosome-scale scaffolding of de novo genome assemblies based on chromatin interactions. Nature Biotechnology *31*, 1119.

Corteggiani Carpinelli, E., Telatin, A., Vitulo, N., Forcato, C., D'Angelo, M., Schiavon, R., Vezzi, A., Giacometti, G.M., Morosinotto, T., and Valle, G. (2014). Chromosome scale genome assembly and transcriptome profiling of *Nannochloropsis gaditana* in nitrogen depletion. Molecular Plant *7*, 323-335.

Dudchenko, O., Batra, S.S., Omer, A.D., Nyquist, S.K., Hoeger, M., Durand, N.C., Shamim, M.S., Machol, I., Lander, E.S., Aiden, A.P.*, et al.* (2017). De novo assembly of the *Aedes aegypti* genome using Hi-C yields chromosome-length scaffolds. Science *356*, 92-95.

Durand, N.C., Shamim, M.S., Machol, I., Rao, S.S.P., Huntley, M.H., Lander, E.S., and Aiden, E.L. (2016). Juicer provides a one-click system for analyzing loop-resolution Hi-C experiments. Cell Systems *3*, 95-98.

Gbadegesin, M.A., and Beeching, J.R. (2010). Enhancer/Suppressor mutator (En/Spm)-like transposable elements of cassava (*Manihot esculenta*) are transcriptionally inactive. Genetics and Molecular Research *9*, 639-650.

Grabherr, M.G., Haas, B.J., Yassour, M., Levin, J.Z., Thompson, D.A., Amit, I., Adiconis, X., Fan, L., Raychowdhury, R., Zeng, Q.*, et al.* (2011). Full-length transcriptome assembly from RNA-Seq data without a reference genome. Nature Biotechnology *29*, 644-652.

Haas, B.J., Delcher, A.L., Mount, S.M., Wortman, J.R., Smith, R.K., Jr., Hannick, L.I., Maiti, R., Ronning, C.M., Rusch, D.B., Town, C.D.*, et al.* (2003). Improving the *Arabidopsis* genome annotation using maximal transcript alignment assemblies. Nucleic Acids Research *31*, 5654-5666.

Haas, B.J., Salzberg, S.L., Zhu, W., Pertea, M., Allen, J.E., Orvis, J., White, O., Buell, C.R., and Wortman, J.R. (2008). Automated eukaryotic gene structure annotation using EVidenceModeler and the Program to Assemble Spliced Alignments. Genome Biology *9*, R7.

Haas, B.J., Zeng, Q., Pearson, M.D., Cuomo, C.A., and Wortman, J.R. (2011). Approaches to fungal genome annotation. Mycology *2*, 118-141.

Hoff, K.J., Lange, S., Lomsadze, A., Borodovsky, M., and Stanke, M. (2016). BRAKER1: unsupervised RNA-Seq-based genome annotation with GeneMark-ET and AUGUSTUS. Bioinformatics *32*, 767-769.

Huerta-Cepas, J., Forslund, K., Coelho, L.P., Szklarczyk, D., Jensen, L.J., von Mering, C., and Bork, P. (2017). Fast genome-wide functional annotation through orthology assignment by eggNOG-Mapper. Molecular Biology and Evolution *34*, 2115-2122.

International Human Genome Sequencing, C., Lander, E.S., Linton, L.M., Birren, B., Nusbaum, C., Zody, M.C., Baldwin, J., Devon, K., Dewar, K., Doyle, M.*, et al.* (2001). Initial sequencing and analysis of the human

genome. Nature *409*, 860.

Jones, P., Binns, D., Chang, H.-Y., Fraser, M., Li, W., McAnulla, C., McWilliam, H., Maslen, J., Mitchell, A., Nuka, G.*, et al.* (2014). InterProScan 5: genome-scale protein function classification. Bioinformatics *30*, 1236-1240.

Kim, D., Langmead, B., and Salzberg, S.L. (2015). HISAT: a fast spliced aligner with low memory requirements. Nature Methods *12*, 357.

Kong, Y., Cao, L., Deikus, G., Fan, Y., Mead, E.A., Lai, W., Zhang, Y., Yong, R., Sebra, R., Wang, H.*, et al.* (2022). Critical assessment of DNA adenine methylation in eukaryotes using quantitative deconvolution. Science *375*, 515-522.

Laetsch, D., and Blaxter, M. (2017). BlobTools: Interrogation of genome assemblies [version 1; referees: 1 approved with reservations]. F1000Research *6*, 1287.

Lagesen, K., Hallin, P., Rodland, E.A., Staerfeldt, H.H., Rognes, T., and Ussery, D.W. (2007). RNAmmer: consistent and rapid annotation of ribosomal RNA genes. Nucleic Acids Research *35*, 3100–3108.

Lam, K.-K., LaButti, K., Khalak, A., and Tse, D. (2015). FinisherSC: a repeat-aware tool for upgrading de novo assembly using long reads. Bioinformatics *31*, 3207-3209.

Lee, T., Peace, C., Jung, S., Zheng, P., Main, D., and Cho, I. (2011). GenSAS — An online integrated genome sequence annotation pipeline. In 2011 4th International Conference on Biomedical Engineering and Informatics (BMEI), pp. 1967-1973.

Li, J., Han, D., Wang, D., Ning, K., Jia, J., Wei, L., Jing, X., Huang, S., Chen, J., Li, Y.*, et al.* (2014). Choreography of transcriptomes and lipidomes of *Nannochloropsis* reveals the mechanisms of oil synthesis in microalgae. The Plant Cell *26*, 1645-1665.

Lowe, T.M., and Eddy, S.R. (1997). tRNAscan-SE: a program for improved detection of transfer RNA genes in genomic sequence. Nucleic Acids Research *25*, 955-964.

Mascher, M., Gundlach, H., Himmelbach, A., Beier, S., Twardziok, S.O., Wicker, T., Radchuk, V., Dockter, C., Hedley, P.E., Russell, J.*, et al.* (2017). A chromosome conformation capture ordered sequence of the barley genome. Nature *544*, 427.

Maumus, F., Allen, A.E., Mhiri, C., Hu, H., Jabbari, K., Vardi, A., Grandbastien, M.-A., and Bowler, C. (2009). Potential impact of stress activated retrotransposons on genome evolution in a marine diatom. BMC Genomics *10*, 624.

McIntyre, A.B.R., Alexander, N., Grigorev, K., Bezdan, D., Sichtig, H., Chiu, C.Y., and Mason, C.E. (2019). Single-molecule sequencing detection of N6-methyladenine in microbial reference materials. Nature Communications *10*, 579.

Nawrocki, E.P., and Eddy, S.R. (2013). Infernal 1.1: 100-fold faster RNA homology searches. Bioinformatics *29*, 2933-2935.

O'Brown, Z.K., Boulias, K., Wang, J., Wang, S.Y., O'Brown, N.M., Hao, Z., Shibuya, H., Fady, P.-E., Shi, Y., He, C.*, et al.* (2019). Sources of artifact in measurements of 6mA and 4mC abundance in eukaryotic genomic DNA. BMC Genomics *20*, 445.

Oddes, S., Zelig, A., and Kaplan, N. (2018). Three invariant Hi-C interaction patterns: Applications to genome assembly. Methods *142*, 89-99.

Pertea, M., Kim, D., Pertea, G.M., Leek, J.T., and Salzberg, S.L. (2016). Transcript-level expression analysis of RNA-seq experiments with HISAT, StringTie and Ballgown. Nature Protocols *11*, 1650-1667.

SanMiguel, P., Tikhonov, A., Jin, Y.-K., Motchoulskaia, N., Zakharov, D., Melake-Berhan, A., Springer, P.S., Edwards, K.J., Lee, M., Avramova, Z.*, et al.* (1996). Nested retrotransposons in the intergenic regions of the maize genome. Science *274*, 765-768.

Schwartz, A.S., Brown, R., Ajjawi, I., McCarren, J., Atilla, S., Bauman, N., and Richardson, T.H. (2018). Complete

genome sequence of the model oleaginous alga *Nannochloropsis gaditana* CCMP1894. Genome Announcements *6*, e01448-01417.

Servant, N., Varoquaux, N., Lajoie, B.R., Viara, E., Chen, C.-J., Vert, J.-P., Heard, E., Dekker, J., and Barillot, E. (2015). HiC-Pro: an optimized and flexible pipeline for Hi-C data processing. Genome Biology *16*, 259.

Soderlund, C., Bomhoff, M., and Nelson, W.M. (2011). SyMAP v3.4: a turnkey synteny system with application to plant genomes. Nucleic Acids Research *39*, e68-e68.

Wang, D., Ning, K., Li, J., Hu, J., Han, D., Wang, H., Zeng, X., Jing, X., Zhou, Q., Su, X., *et al.* (2014). *Nannochloropsis* genomes reveal evolution of microalgal oleaginous traits. PLOS Genetics *10*, e1004094.

Wei, L., El Hajjami, M., Shen, C., You, W., Lu, Y., Li, J., Jing, X., Hu, Q., Zhou, W., Poetsch, A., *et al.* (2019). Transcriptomic and proteomic responses to very low CO_2 suggest multiple carbon concentrating mechanisms in *Nannochloropsis oceanica*. Biotechnology for Biofuels *12*, 168.

Zhang, G., Huang, H., Liu, D., Cheng, Y., Liu, X., Zhang, W., Yin, R., Zhang, D., Zhang, P., and Liu, J. (2015). N6-methyladenine DNA modification in *Drosophila*. Cell *161*, 893-906.

Zhou, C., Wang, C., Liu, H., Zhou, Q., Liu, Q., Guo, Y., Peng, T., Song, J., Zhang, J., Chen, L., *et al.* (2018). Identification and analysis of adenine N6-methylation sites in the rice genome. Nature Plants *4*, 554-563.

Supplemental Tables

Table S1. Statistics of reads from the PacBio RS-II sequencing. Whole genomic DNA of *N. oceanica* IMET1 was sequenced by 19 SMRT cells from PacBio RS-II system and P6-C4 chemistry on a size selected 20kb library. After base calling, 1.13 million polymerase reads, which account for ~340X sequencing depth, were obtained. After adapter trimming and quality filtering, 1.49 million PacBio subreads remained for genome assembly and base modification analysis with subread N50 length of 9.43 kb.

Metrics	Pre-filter	Post-filter	PacBio subreads
Number of bases	11.667 Gb	10.786 Gb	10.766 Gb
Number of reads	2,855,548	1,127,196	1,489,782
Read N50	12,628	13,070	7,226
Mean read length	4,085	9,568	9,431
Mean read quality	0.355	0.815	-

Table S2. Genome assembly metrics of *N. oceanica*. The chromosome resolved assembly NoIMET1v2 had 9.8-fold higher contiguity than NoIMET1v1 and used as reference for calling DNA 6mA modifications.

Metrics	NoIMET1v1	IMET1pb	NoIMET1v2
# contigs	294	70	30*
Total bases	31.5 Mb	31.0 Mb	31.0 Mb
Avr. length	107.2 Kb	443 Kb	1.034 Mb
N25 length	1.171 Mb	1.332 Mb	1.471 Mb
N50 length	935.2 Kb	1.080 Mb	1.168 Mb
N90 length	150.5 Kb	248.9 Kb	685.9 Kb
GC %	47.79%	54.33%	54.33%
Ns	11.05%	0.00%	0.01%

Table S3. Comparison of TEs among multiple species. For algae and plants, super families from Class II TEs (DNA transposons) rarely become the most abundant TE type.

Species	TEs	Top TE super family	CMC-EnSpm
A. thaliana	19.5%	RC/Helitron (6.3%)	0.98%
C. reinhardtii	12.5%	LINEs (3.84%)	-
V. carteri	20.4%	LTR (3.68%)	-
P. tricornutum	6.4%	Ty1/Copia (~5.7%)	-
T. pseudonana	1.9%	Ty1/Copia (~0.9%)	-
O. sativa	19.9%	Ty3/gypsy (7.3%)	-
Z. mays	64.0%	LTR/RLG (34.88%)	-
S. cerevisiae	3.4%	LTR/Ty1-Ty5	-
human	44.0%	ALU	-
zebrafish	51.2%	DNA (19.31%)	2.19%
N. oceanica	16.2%	DNA/CMC-EnSpm	3.84%

Table S4. Statistics for genome annotation of *N. oceanica*. NoIMET1v2 was selected as reference sequence for the annotation. The annotation showed that 90.5% bases are covered by genes and 2,442 genes are overlapped.

Items		Number/Percent
**Structural prediction**		
Number of genes		10,333
Number of mRNAs		12,442
Number of exons		51,592
Number of introns		39,150
Number of CDS		12,442
Overlapping genes		2,442
Contained genes		628
Mean gene length		2,715
Mean mRNA length		2,890
Mean exon length		554
Mean intron length		190
Mean CDS length		1,639
Covered by genes		90.5%
Covered by CDS		65.8%
Mean mRNAs per gene		1
Mean exons per mRNA		4
Mean introns per mRNA		3
**Functional annotation**		
NR		10,994 (88.4%)
InterProScan		9,673 (77.7%)
EggNOG	GO	5,087 (40.9%)
EggNOG	KO	5,728 (46.0%)
EggNOG	COG	7,972 (64.1%)

Table S5. List of DNA 6mA associated genes in *N. oceanica*. In human, N6AMT1 was functionally validated as DNA 6mA methyltransferase. The putative DNA 6mA methyltransferases were selected based on homologous analysis.

Type	Organism	Gene id	Protein length
DNA 6mA methyltransferase	*Arabidopsis*	AT3G13440.1	278
	Rice	XP_015631415.1	285
	Chlamydomonas	Cre01.g036750.t1.2	223
	P. sojae	XP_009527935.1	209
	Human	N6AMT1	217
	Mouse	NP_080642.1	214
	Nannochloropsis	NO08G00280.1	234
	L. transversale	XP_021883074.1	215
	Drosophila	NP_001027221.1	224
	Tetrahymena	XP_001015373.1	257
DNA 6mA demethylase	*Nannochloropsis*	NO06G02500.1	506
	Chlamydomonas	Cre06.g278198.t1.1	558
	Arabidopsis	AT1G11780.1	344
	Rice	LOC_Os03g60190	370
	Human	ALKBH1	388

Table S6. Statistics of 6mA detection in the mock dataset for accessing potential bacterial and fungal contamination. The penultimate column records the number of 6mA sites detected from the mock datasets which augmented PacBio sequencing data of *N. oceanica* in this study. The last row represents a mock dataset which combines all the PacBio sequencing data from the eight microorganisms on the list. 6mA sites were detected based on the reference genome of *N. oceanica* IMET1 in this study. Based on only *N. oceanica* data, 24,450 6mA sites were detected.

**Species**	**SRA accession**	**Subread bases (G)**	**Mapped bases**	**# 6mA**	**# 6mA***	**Shared 6mA**
Bacillus subtilis	SRR7498042	0.94	0.08‰	4	24,450	100.0%
Enterococcus faecalis	SRR7415622	1.13	0.07‰	2	24,447	100.0%
Escherichia coli	SRR8154667<br>SRR8154668<br>SRR8154669<br>SRR8154675<br>SRR7498041<br>SRR7498044	4.43	0.01‰	4	24,449	100.0%
Listeria monocytogenes	SRR7415624	0.71	0.05‰	0	24,449	100.0%
Pseudomonas aeruginosa	SRR7498043	0.70	0.01‰	0	24,452	100.0%
Saccharomyces cerevisiae	SRR7498045<br>SRR7498046<br>SRR7498048	1.07	0.72‰	41	24,463	99.9%
Salmonella enterica	SRR7415626	0.56	0.01‰	0	24,453	100.0%
Staphylococcus aureus	SRR7415627	0.60	0.10‰	1	24,452	100.0%
Altogether	-	-	-	-	24,466	99.8%

Table S7. Characteristics of DNA 6mA between the *N. oceanica* and *C. reinhardtii* genomes.

	N. oceanica **(stramenopiles)**	***C. reinhardtii*** **(green algae)**
**Global 6mA level (6mA/A)**	~0.1%	~0.4%
**6mA preference (motif)**	ApT	AGGYV, GAGWG, GAVGT, etc.
**Enrichment in specific gene structure**	Splicing donor site (1-2 bp upstream) and 3'UTR	TSS
**Function in gene transcription**	Positive correlation with gene transcription	Marking active TSS

Table S8. Oligonucleotides used in the *in vitro* methylation and demethylation assays.

**6mA-oligo**	5’-CATGATACCTTATGGAA*(6mA)AGCATGCTTGTATTTCTTATGAACCATGA<br>TACCTTATGGAAAGCATGCTTGTATTTCTTATGAAC-3’
**NC-oligo**	5’-CATGATACCTTATGGAAAGCATGCTTGTATTTCTTATGAACCATGA<br>TACCTTATGGAAAGCATGCTTGTATTTCTTATGAAC-3’

Table S9. List of accession numbers for the sequence data used in this article. All the sequencing data were shared using public databases according to the listed accession numbers.

No.	Accession numbers	Description
1	SRR2022894-SRR2022912	19 PacBio sequencing runs
2	SRR8420587	Hi-C sequencing data
4	SRP017310	N+/N- mRNA-seq datasets
5	GSE55861	C+/C- mRNA-seq datasets
6	GSE178672	RNA-seq datasets for wild-type and mutants under high light
7	GSE212585	PacBio Sequel II sequencing data for the wild-type and mutants under high light
8	https://nandesyn.single-cell.cn/download	All the assemblies and other omics resources

Supplemental Figures

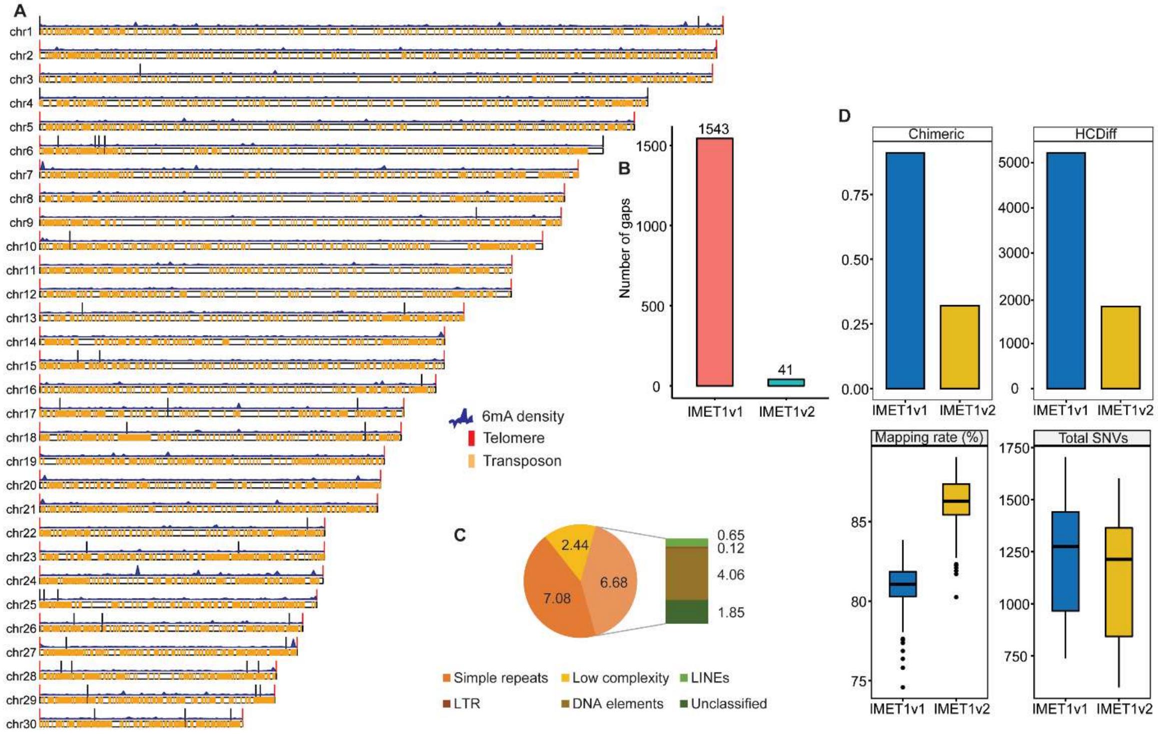

Figure S1. The improved genome sequence of *N. oceanica*. (**A**) The thirty chromosomes of NoIMET1v2. Notable features are shown above each bar with telomeres as red stripes, gaps as black stripes, and 6mA densities as blue curves (in 5 kb resolution). Transposons are shown inside each bar as orange regions. (**B**) Comparison between NoIMET1v1 and NoIMET1v2 in the number of gaps. (**C**) Repeat statistics for NoIMET1v2. DNA elements exceed retrotransposons (e.g., LTR and LINES) to become the most abundant transposons. (**D**) Evaluation of NoIMET1v2 using 454 and RNA-Seq data. Genomic (top) and transcriptomic (bottom) analysis reveal that the new assembly NoIMET1v2 is more complete than NoIMET1v1 and possesses bases with higher quality.

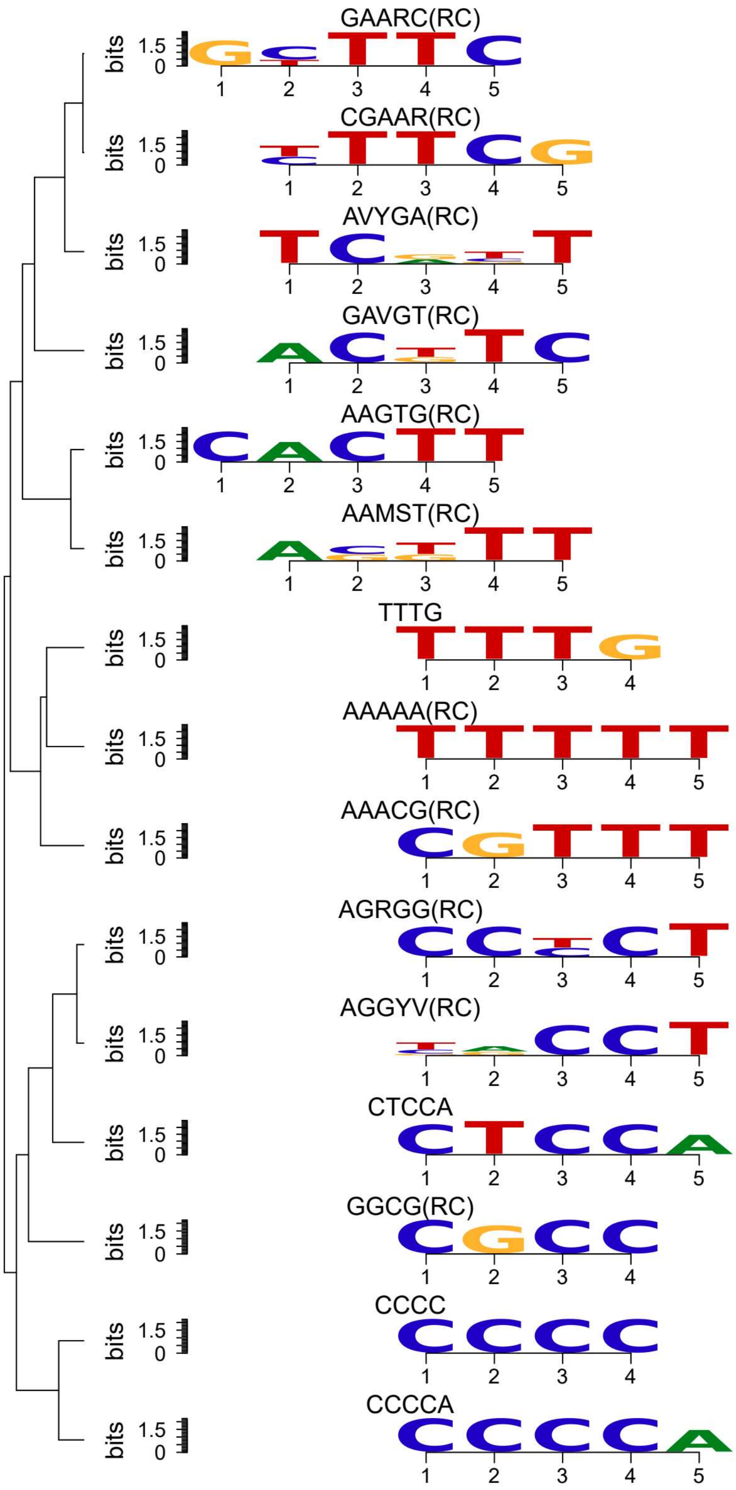

Figure S2. DNA 6mA enriched motifs in the *N. oceanica* genome. Totally 15 motifs were enriched in 6mA modified DNA fragments.

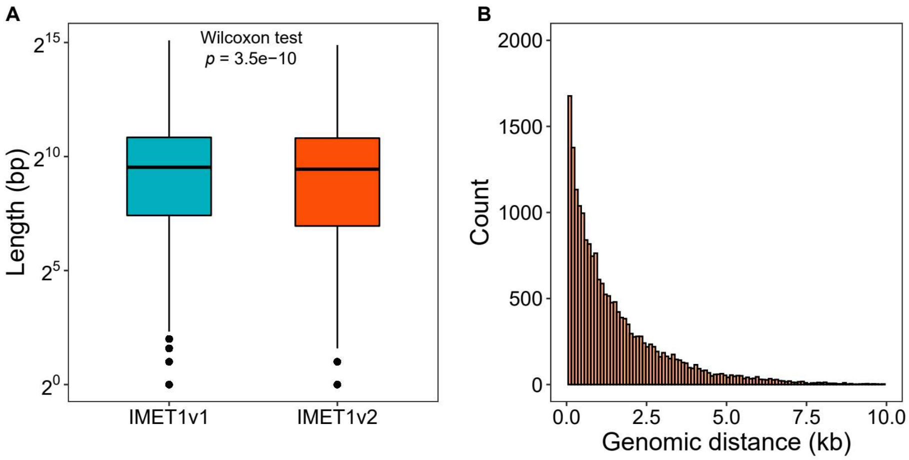

Figure S3. Distribution of 6mA events in the *N. oceanica* genome. (**A**) Distances of nearby 6mA sites detected using NoIMET1v1 and NoIMET1v2 as references. (**B**) Distribution of 6mA interval distances. Over 78% 6mA sites were with distance to nearest 6mA site within 2 kb which will affect the enrichment of 6mA-antibody.

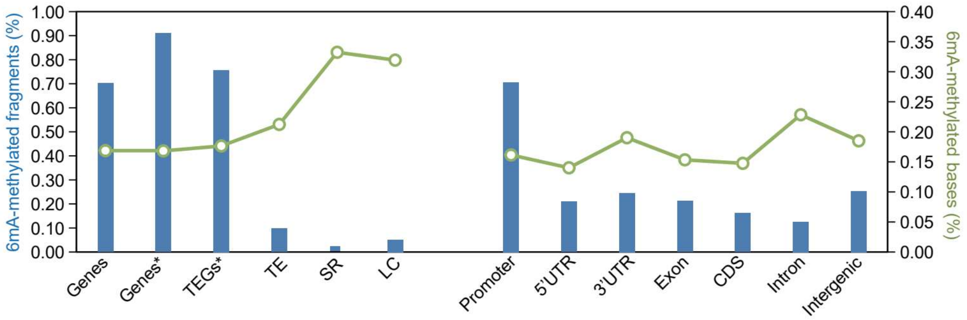

Figure S4. Global features of the 6mA events in the *N. oceanica* genome. The proportions and base proportions of 6mA events for different genomic regions (both strands). Genes: gene body; Genes*: gene body and 2kb promoter regions; TEGs*: Genes* overlapped with TEs; SR: simple repeats. LC: low complexity fragments; UTR: untranslated region.

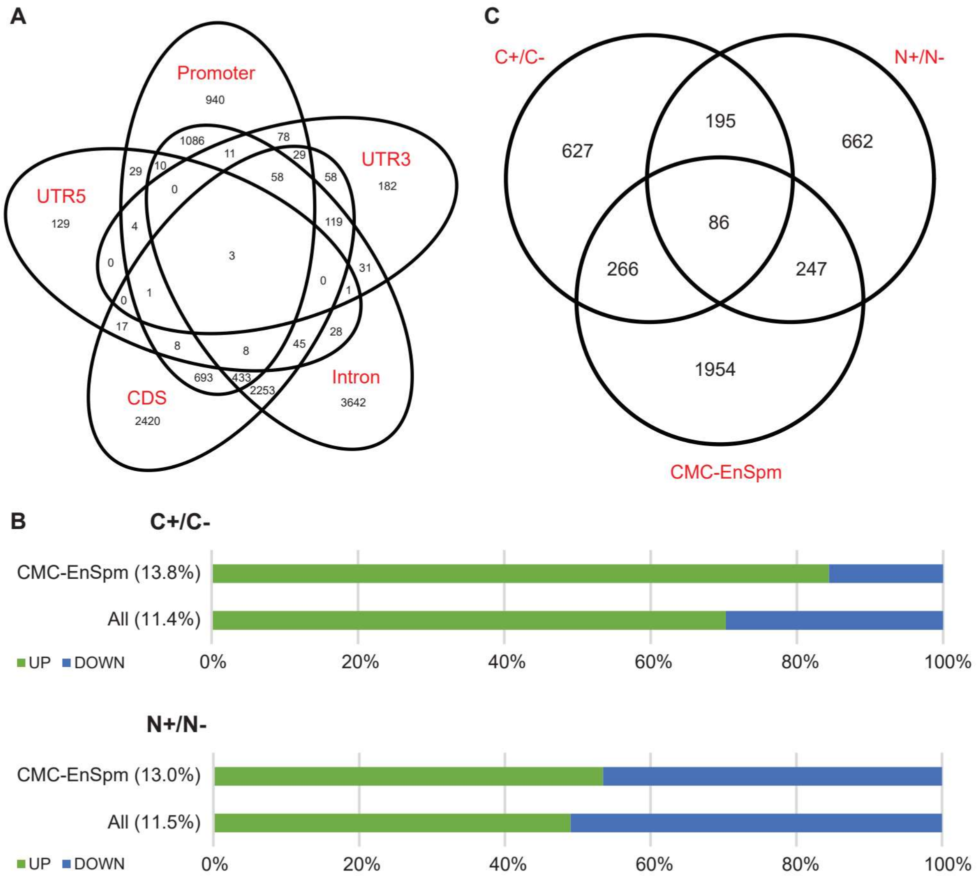

Figure S5. Transposable elements repress gene transcription in the *N. oceanica* genome. (**A**) Position statistics of TEs. (**B**) Many EnSpm-TEGs were differentially expressed with response to nitrate or CO_2 limitation. (**C**) Venn diagrams show relationships between nitrogen-depletion induced DEGs, CO_2-depletion induced DEGs and EnSpm-TEGs.

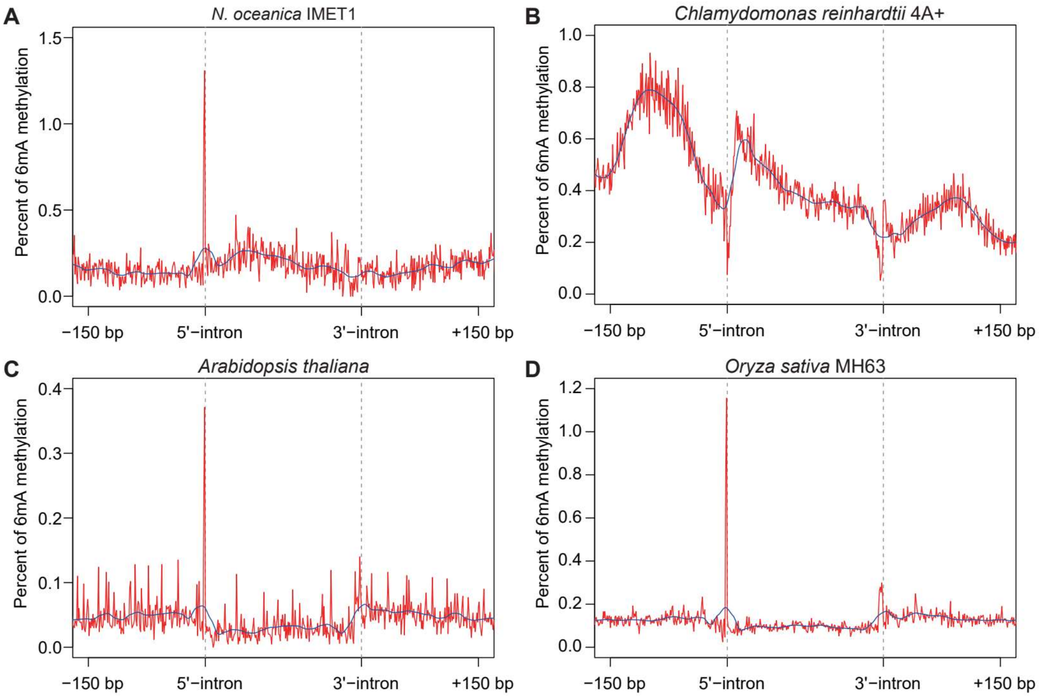

Figure S6. 6mA occupancy around intron (strand-specific) for *N. oceanica* (A), *Chlamydomonas reinhardtii* (B), *Arabidopsis thaliana* (C), and *Oryza sativa* (D). The strand of intron was defined as the strand of the same gene, each intron was consolidated into 200 bp. The 6mA occupancy data around intron was plotted with and without smoothing (blue and red).

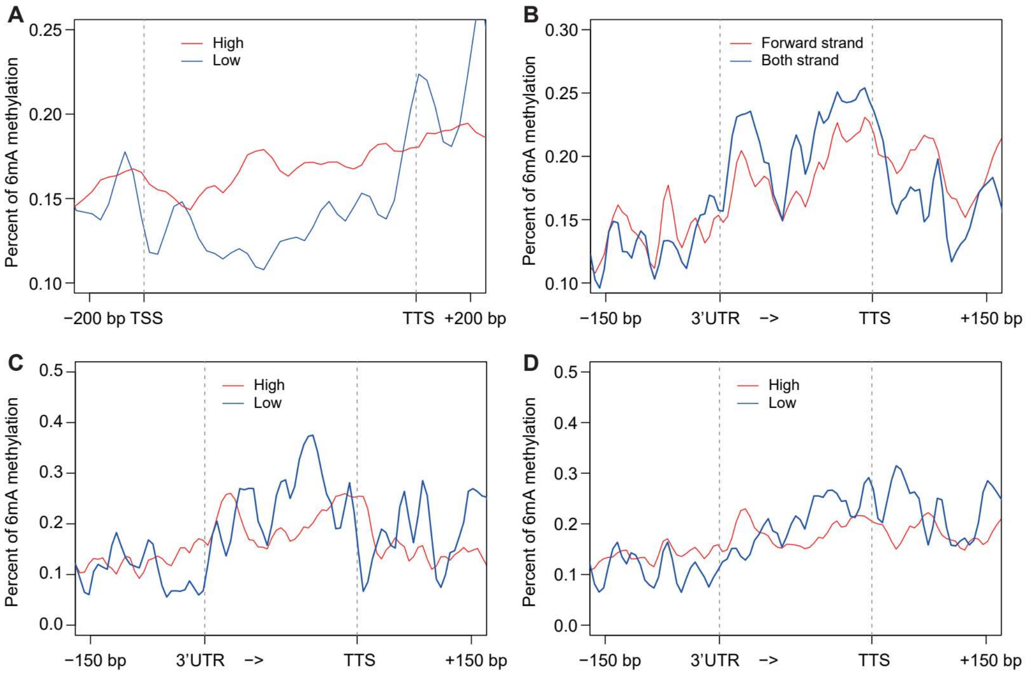

Figure S7. DNA 6mA occupancy along the genes and the TSSs in the *N. oceanica* genome. (**A**) 6mA occupancy along genes (non-strand-specific) for highly expressed genes (High: TPM>100) and poorly expressed genes (Low: TPM⩽100). (**B**) 6mA occupancy along 3'UTRs (strand-specific: red line; non-strand-specific: blue line). (**C**) 6mA occupancy along 3'UTRs (strand-specific) for highly expressed genes (High: TPM>100) and poorly expressed genes (Low: TPM⩽100). (**D**) 6mA occupancy along 3'UTRs (non-strand-specific) for highly expressed genes (High: TPM>100) and poorly expressed genes (Low: TPM⩽100).

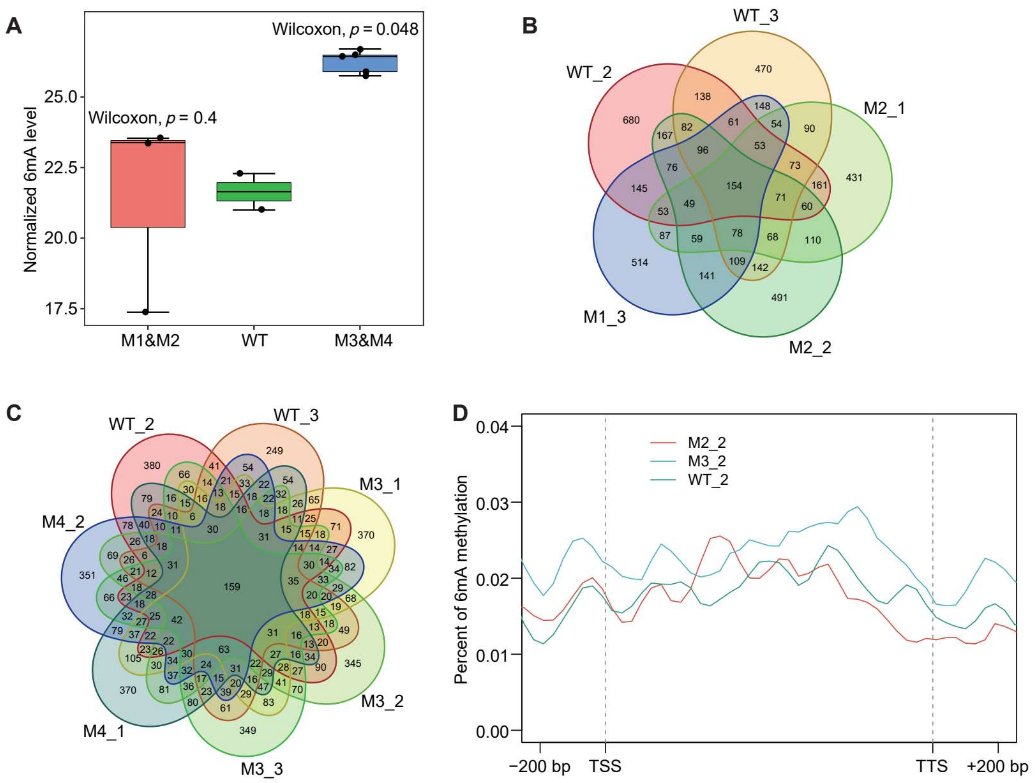

Figure S8. Characteristics of 6mA distribution in the wild-type versus the mutants of *N. oceanica*. (**A**) Comparison of 6mA levels in wild-type versus mutants. M1&M2 group includes Mutant 1 and 2; M3&M4 group contains Mutant 3 and 4. PacBio sequencing data for all the samples are subsampled to ~100X mappable subreads, then the 6mA levels are normalized (divide by) according to the final average base coverage. One-tailed Wilcoxon test is used to compare wild-type and mutant groups. (**B**) Overlap of 6mA-marked genes in M1, M2 and WT. (**C**) Overlap of 6mA-marked genes in M3, M4 and WT. (**D**) 6mA/A along protein-coding genes for M2, M3 and WT. One replicate for each group is shown.

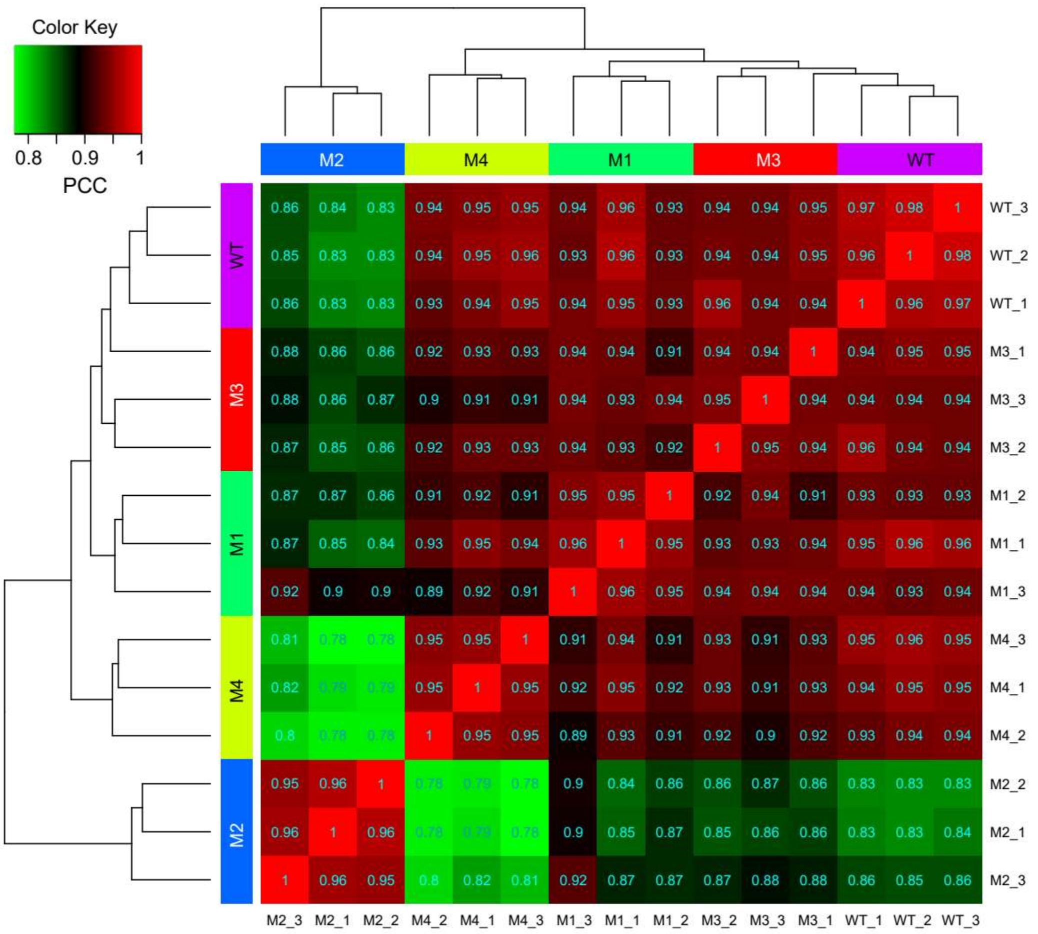

Figure S9. Sample correlation matrix for the wild-type and the knockout mutants of *N. oceanica* based on RNA-seq data. PCC: Pearson correlation coefficient. Algal cells were cultivated under high light and collected at 7 days. Mutants M1 and M2 are NO08G00280-knockout mutants; M3 and M4 are NO06G02500-knockout mutants. Expression values of differentially expressed genes were used to calculate the similarity between samples. All PCCs between replicates are > 0.94, which revealed excellent reproducibility among the biological replicates.
